# *TERT* promoter hotspot mutations and gene amplification in metaplastic breast cancer

**DOI:** 10.1038/s41523-021-00250-8

**Published:** 2021-04-16

**Authors:** Edaise M. da Silva, Pier Selenica, Mahsa Vahdatinia, Fresia Pareja, Arnaud Da Cruz Paula, Lorenzo Ferrando, Andrea M. Gazzo, Higinio Dopeso, Dara S. Ross, Ariya Bakhteri, Nadeem Riaz, Sarat Chandarlapaty, Pedram Razavi, Larry Norton, Hannah Y. Wen, Edi Brogi, Britta Weigelt, Hong Zhang, Jorge S. Reis-Filho

**Affiliations:** 1 Department of Pathology, Memorial Sloan Kettering Cancer Center, New York, NY USA; 2 Department of Surgery, Memorial Sloan Kettering Cancer Center, New York, NY USA; 3 Department of Internal Medicine, University of Genoa, Genova, Italy; 4 Department of Radiation Oncology, Memorial Sloan Kettering Cancer Center, New York, NY USA; 5 Department of Medicine, Memorial Sloan Kettering Cancer Center, New York, NY USA

**Keywords:** Cancer genomics, Cancer genetics, Breast cancer

## Abstract

Metaplastic breast cancers (MBCs) are characterized by complex genomes, which seem to vary according to their histologic subtype. *TERT* promoter hotspot mutations and gene amplification are rare in common forms of breast cancer, but present in a subset of phyllodes tumors. Here, we sought to determine the frequency of genetic alterations affecting *TERT* in a cohort of 60 MBCs with distinct predominant metaplastic components (squamous, 23%; spindle, 27%; osseous, 8%; chondroid, 42%), and to compare the repertoire of genetic alterations of MBCs according to the presence of *TERT* promoter hotspot mutations or gene amplification. Forty-four MBCs were subjected to: whole-exome sequencing (WES; *n* = 27) or targeted sequencing of 341-468 cancer-related genes (*n* = 17); 16 MBCs were subjected to Sanger sequencing of the *TERT* promoter, *TP53* and selected exons of *PIK3CA*, *HRAS*, and *BRAF*. *TERT* promoter hotspot mutations (*n* = 9) and *TERT* gene amplification (*n* = 1) were found in 10 of the 60 MBCs analyzed, respectively. These *TERT* alterations were less frequently found in MBCs with predominant chondroid differentiation than in other MBC subtypes (*p* = 0.01, Fisher’s exact test) and were mutually exclusive with *TP53* mutations (*p* < 0.001, CoMEt). In addition, a comparative analysis of the MBCs subjected to WES or targeted cancer gene sequencing (*n* = 44) revealed that MBCs harboring *TERT* promoter hotspot mutations or gene amplification (*n* = 6) more frequently harbored *PIK3CA* than *TERT* wild-type MBCs (*n* = 38; *p* = 0.001; Fisher’s exact test). In conclusion, *TERT* somatic genetic alterations are found in a subset of *TP53* wild-type MBCs with squamous/spindle differentiation, highlighting the genetic diversity of these cancers.

## Introduction

Metaplastic breast cancer (MBC) is a rare (0.2–1%)^1,2^, aggressive histologic subtype of breast cancer, characterized histologically by neoplastic epithelium displaying differentiation towards squamous or mesenchymal elements, including spindle, chondroid, osseous, or rhabdoid cells. MBCs can present one (monophasic) or two or more (biphasic) components. These components can both display metaplastic histology or can be one metaplastic component and one adenocarcinoma component, which is most commonly in the form of invasive ductal carcinoma of no special type (IDC-NST). MBCs are most often of high histologic grade and display a triple-negative phenotype^1,3^.

The histologic diversity of MBCs is associated with distinct genomic and transcriptomic profiles^4–12^. From a genetic standpoint, MBCs are shown to frequently harbor mutations affecting *TP53* and genes related to the PI3K/AKT/mTOR pathways. While *TP53* mutations are found to be less frequent in MBCs with prominent spindle cell component, *PIK3CA* mutations are vanishingly rare in MBCs with chondroid metaplasia^7,12,13^. The transcriptomic features of MBCs also vary according to the predominant histologic component; for instance, MBCs with a predominant spindle cell component are preferentially classified as of claudin-low subtype, whereas MBCs with squamous or chondroid metaplasia are more frequently classified as of basal-like or even normal breast-like subtypes than as of claudin-low subtype^8,9^.

Somatic *TERT* promoter mutations (C228T and C250T), associated with telomerase activation, have been reported at a relatively high frequency in human cancers (12% overall)^14^ and are associated with disease progression and recurrences^15–17^. Although thought to be absent or extremely rare in common forms of breast cancer^18–20^, *TERT* promoter mutations and *TERT* gene amplifications have been reported in up to 68% of malignant phyllodes tumors of the breast, a potential differential diagnosis of MBCs, and may have a role in the malignant progression in fibroepithelial lesions^15,21,22^. *TERT* gene amplification has been reported in 13% of adenomyoepitheliomas of the breast, tumors of uncertain malignant potential which have been reported to progress to spindle cell MBCs in a minority of cases^23,24^. Interestingly, in one adenomyoepithelioma progressing to a triple-negative spindle cell MBC, the submodal clone that most likely gave rise to the invasive carcinoma harbored a *TERT* promoter hotspot mutation^24^. In the context of MBCs, *TERT* promoter hotspot mutations have been reported in up to 25% of cases, and to be associated with MBCs with spindle and/or squamous differentiation^12^.

Here, we sought to determine the frequency of genetic alterations affecting *TERT*, including *TERT* promoter hotspot mutations and *TERT* gene amplifications in MBCs with distinct types of predominant metaplastic component (e.g. squamous, spindle cell, osseous and chondroid). We have also compared the repertoire of somatic genetic alterations of MBCs harboring *TERT* promoter mutations or gene amplification to MBCs lacking genetic alterations targeting *TERT*. These analyses have revealed that 17% (10 out of 60) of MBCs harbor *TERT* somatic genetic alterations, and that these are associated with specific predominant metaplastic components and are seemingly mutually exclusive with *TP53* mutations.

## Results

### Clinicopathologic characteristics

**Fig. 1:**
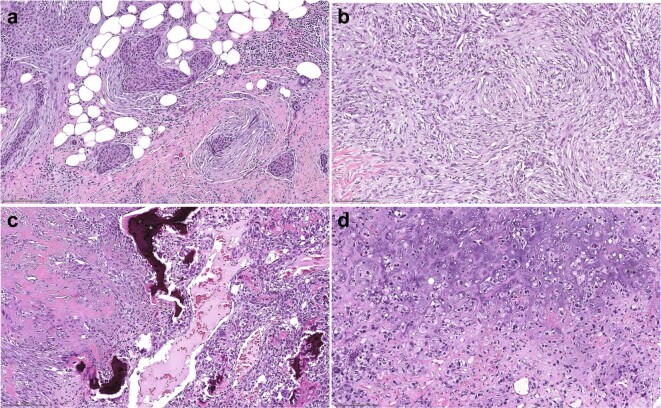
Histologic features of the metaplastic breast cancers included in this study. Representative hematoxylin-and-eosin photomicrographs of metaplastic breast cancers (MBCs) with predominant **a** squamous cell carcinoma component (MBC103T), **b** spindle cell component (MBC118T), **c** osseous metaplasia component (MBC120T), and **d** chondroid metaplasia component (MBC108T). Scale bars, 200 μM.

Sixty primary MBCs were included in this study (Table 1 and Supplementary Table S1), including 4 biopsies and 56 resection specimens; of these specimens, 6 (5 resections and 1 biopsy) were obtained post neoadjuvant therapy. The median age at diagnosis was 57 years old (range 34–85). Most (92%, 55/60) MBCs were of histologic grade 3 and 95% (57/60) of the MBCs analyzed were of triple-negative phenotype (Table 1). Upon central histopathological review, the MBCs included in this study were classified according to their predominant histologic type into squamous (23%; 14/60), spindle cell (27%; 16/60), osseous (8%; 5/60), or chondroid (42%, 25/60) MBCs (Fig. 1 and Supplementary Table S1). Forty-seven percent (28/60) of the MBCs were matrix-producing, including MBCs with predominant chondroid (*n* = 24) and osseous (*n* = 4; Table 1 and Supplementary Table S1) histologic components.

**Table 1:** Clinicopathologic features of 60 metaplastic breast carcinomas included in this study.

			Predominant histologic component			
		MBCs	SQUAMOUS	SPINDLE	OSSEOUS	CHONDROID
		(*n* = 60)	(*n* = 14)	(*n* = 16)	(*n* = 5)	(*n* = 25)
Histologic grade^a^	2	5 (8%)	1 (7%)	1 (6%)	0	3 (12%)
	3	55 (92%)	13 (93%)	15 (94%)	5	22 (88%)
Matrix producing	No	32 (53%)	14 (100%)	16 (100%)	1 (20%)	1 (4%)
	Yes	28 (47%)	0	0	4 (80%)	24 (96%)
ER status	Negative	59 (98%)	13 (93%)	16 (100%)	5 (100%)	25 (100%)
	Positive	0	0	0	0	0
	Not available	1 (2%)	1 (7%)	0	0	0
PR status	Negative	59 (98%)	13 (93%)	16 (100%)	5 (100%)	25 (100%)
	Positive	0	0	0	0	0
	Not available	1 (2%)	1 (7%)	0	0	0
HER2 status	Negative	57 (95%)	11 (79%)	16 (100%)	5 (100%)	25 (100%)
	Positive	2 (3%)	2 (14%)	0	0	0
	Not available	1 (2%)	1 (7%)	0	0	0
Triple-negative phenotype	*n* (%)	57 (95%)	11 (79%)	16 (100%)	5 (100%)	25 (100%)

*ER,* estrogen receptor*; PR,* progesterone receptor.

^a^Nottingham grading system.

### *TERT* genetic alterations in MBCs

**Fig. 2:**
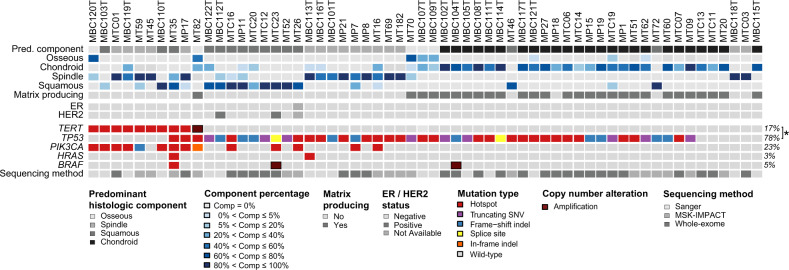
Recurrent somatic *TERT, TP53, PIK3CA*, *HRAS*, and *BRAF* genetic alterations in distinct histologic subtypes of 60 metaplastic breast cancers. Heatmap depicting the proportion of the histologic component, frequency of *TERT* genetic alterations, *TP53* mutations, *PIK3CA* mutations, *HRAS* mutations and *BRAF* genetic alterations in 60 MBCs. Mutation types are color-coded according to the legend. Cases are shown in columns, the percentage of the histological components, matrix producing, ER and HER2 status, and sequencing methods are depicted in phenobars. *Mutual exclusivity analysis, CoMEt, *p* < 0.001.

We first sought to determine the frequency of *TERT* genetic alterations in 60 MBCs included in this study. Genetic alterations affecting *TERT* were identified in 17% (10/60) of the MBCs, including a recurrent hotspot mutation affecting the *TERT* promoter hotspot locus (C228T; 15%, 9/60) and *TERT* gene amplification (2%, 1/60; Fig. 2). All MBCs harboring *TERT* promoter alterations were of triple-negative phenotype (Fig. 2). We next performed a hypothesis-generating, exploratory analysis of the associations between the presence of *TERT* somatic genetic alterations and the phenotype of MBCs (Fig. 2 and Supplementary Fig. S1). This analysis revealed that *TERT* genetic alterations were significantly less frequently found in MBCs with a predominant chondroid component (0/25) than in the remaining MBCs (10/35; *p* = 0.005, Fisher’s exact test, Table 2 and Fig. 2). Nonetheless, two of these 10 MBCs harboring *TERT* genetic alterations including a predominant spindle cell MBC (MBC119T, *TERT* promoter mutation) and a predominantly osseous MBC (MT82, *TERT* gene amplification) displayed focal areas of chondroid differentiation (Fig. 2).

**Table 2:** Frequency of genetic alterations affecting *TERT* gene in 60 metaplastic breast cancers according to their predominant histologic component.

		Predominant histologic component				
	MBCs	SQUAMOUS	SPINDLE	OSSEOUS	CHONDROID	*p* value^a^
	(*n* = 60)	(*n* = 14)	(*n* = 16)	(*n* = 5)	(*n* = 25)	
*TERT* genetic alteration	10 (17%)	3 (21%)	5 (31%)	2 (40%)	0	0.005
*TERT* wild-type	50 (83%)	11 (79%)	11 (69%)	3 (60%)	25 (50%)	

^a^Statistical significance was evaluated by Fisher’s exact test.

### Comparative analysis between *TERT* altered MBCs and *TERT* wild-type MBCs

**Fig. 3:**
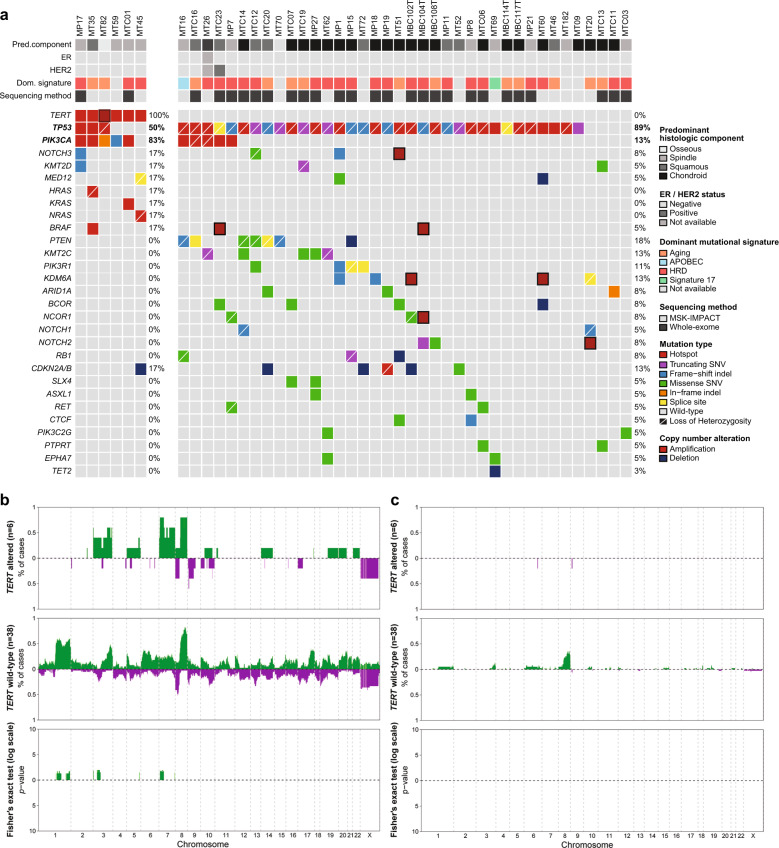
Repertoire of non-synonymous somatic mutations identified in metaplastic breast cancers. **a** Comparison of the most frequent genetic alterations affecting cancer genes identified in metaplastic breast cancers harboring *TERT* genetic alterations (*TERT* promoter hotspot mutations, *n* = 5; *TERT* gene amplification, *n* = 1; left) and *TERT* wild-type (*n* = 38, right), by whole-exome sequencing or MSK-IMPACT targeted sequencing. Cases are shown in columns and genes in rows. Clinicopathologic characteristics are shown on the top. Mutations are color coded according to the legend. Frequency plots and Fisher’s exact test comparison corrected for multiple testing of (**b**) copy number gains and losses, and (**c**) amplifications and homozygous deletions between *TERT* altered (*n* = 6) and *TERT* promoter wild-type (n = 38) MBCs. Frequency (y-axis) of gains and losses and amplifications and homozygous deletions is shown for each genomic region (x-axis). Inverse Log_10_ values of the two-sided Fisher’s exact test p-values are plotted according to the genomic region (lower panel). Gains and amplifications are colored in green. Losses and homozygous deletions are colored in purple. *Statistical significance was evaluated by the Fisher’s exact test (*p* < 0.05). **Mutual exclusivity analysis was performed using combinations of mutually exclusive alterations (CoMEt, *p* < 0.01). HRD, homologous recombination DNA repair defect; SNV, single nucleotide variants.

We next sought to define whether the six MBCs harboring *TERT* gene promoter hotspot mutations or gene amplification displayed a distinct repertoire of somatic genetic alterations as compared to the 38 *TERT* wild-type MBCs (Fig. 3, Supplementary Table S2) subjected to whole-exome sequencing (WES) or MSK-IMPACT targeted sequencing of 341–468 cancer-related genes. In our study, MBCs harboring *TERT* genetic alterations had a tumor mutation burden (median 3.1 mutations/Mb; range 0.8–6.1) comparable to that of MBCs lacking genetic alterations affecting *TERT* (median 3.5 mutations/Mb; range 0.8–8.7; *p* = 0.72, Mann–Whitney *U* test; Supplementary Fig. S2a). Our exploratory analysis revealed that, despite having similar tumor mutation burden, MBCs harboring *TERT* genetic alterations were significantly enriched for *PIK3CA* clonal mutations preferentially affecting hotspots (5/6, 83% *TERT* altered *vs* 5/38, 13% *TERT* WT; *p* = 0.001, Fisher’s exact test). Four of the 5 MBCs harbored clonal *TERT* promoter hotspot mutations co-occurring with *PIK3CA* mutations, and one MBC (MT45) that lacked mutations affecting *PIK3CA* harbored a subclonal *TERT* promoter mutation (Fig. 3a and Supplementary Fig. S3). *TP53* mutations were significantly more frequently detected in MBCs lacking genetic alterations affecting *TERT* (34/38, 89% *TERT* wild-type *vs* 3/6, 50% *TERT* altered; *p* = 0.04, Fisher’s exact test; Fig. 3a). A formal mutually exclusivity analysis based on CoMEt^25^ in these 44 MBCs demonstrated that *TP53* mutations were significantly mutually exclusive with *TERT* genetic alterations (*p* < 0.01, CoMEt). This observation was further confirmed when the entire cohort (*n* = 60) of MBCs was analyzed (*p* < 0.001, CoMEt; Fig. 2).

Although no other gene was significantly differentially altered between *TERT* altered vs wild-type MBCs (*p* > 0.05; Fisher’s exact test, Fig. 3a), mutations affecting *PTEN*, *PIK3R1*, chromatin remodeling genes (*ARID1A*, *KMT2C*) and tumor suppressor genes (*RB1*, *NOTCH1*, and *NOTCH2*) were only identified in MBCs lacking *TERT* genetic alterations (Fig. 3a). In addition, activating mutations affecting Ras pathway genes were detected in three MBCs harboring *TERT* promoter hotspot mutations, including hotspot mutations affecting *KRAS* (MTC01, subclonal A59G, *n* = 1), *NRAS* (MT45, clonal Q61L, *n* = 1) and *HRAS* Q61R (MT35, clonal, *n* = 1), which coexisted with mutations affecting *TP53* (subclonal V173L and clonal E204Vfs*4) and a subclonal *BRAF* D594N hotspot mutation (Fig. 3a; Supplementary Fig. S3). Two *TERT* wild-type MBCs harbored *BRAF* amplification (MTC23 and MBC104T; Fig. 3a). Similar observations were made when the 16 additional MBCs were subjected to Sanger sequencing of *HRAS* and *BRAF* hotspot loci (Fig. 2). This additional analysis revealed another MBC (MBC113T) harboring an *HRAS* hotspot mutation (Q61K, Fig. 2) co-occurring with a *TP53* hotspot mutation (D281E) but did not identify mutations affecting *TERT* promoter or *PIK3CA* hotspot locus.

Given the previous observation that *HRAS* Q61R hotspot mutations co-occurring with *TERT* promoter mutations were preferentially found in adenomyoepitheliomas^24^, we sought to define whether the MBCs harboring *HRAS* Q61 hotspot mutations would be associated with or originate from adenomyoepitheliomas. Not surprisingly, the *HRAS* Q61 mutant MBCs identified in our study lacked histologic features of adenomyoepithelioma, unlike TNBCs originating from adenomyoepithelioma, which have been shown to lack *TP53* mutations and consistently harbor *PIK3CA* or *PIK3R1* mutations^24^. Upon re-review of the MBCs, including all diagnostic slides available per case harboring *HRAS* Q61 mutations, only one MBC (MBC103T; Supplementary Fig. S4) was found to display features consistent with the presence of a breast adenomyoepithelioma. This MBC contained a biphasic proliferation of epithelial and myoepithelial cells (Supplementary Fig. S4), where the abluminal layer expressed p63 and calponin by immunohistochemical analysis, consistent with a diagnosis of MBC developing in the context of an adenomyoepithelioma. Sanger sequencing analysis of this case revealed mutations affecting *TERT* promoter (C228T) and *PIK3CA* hotspot locus (H1047R), but no *TP53* mutations or alterations in *HRAS* codon Q61 (Fig. 2).

Here we demonstrate that, thirty-eight of the 44 MBCs subjected to WES or MSK-IMPACT sequencing had sufficient single nucleotide variants (SNVs) to infer accurate mutational signature (Fig. 3a, Supplementary Table S1). Based on SigMA analysis, an algorithm previously validated for the analysis of formalin-fixed paraffin-embedded (FFPE) samples subjected to the FDA-approved MSK-IMPACT sequencing assay, 23 MBCs (60%, 23/38) displayed dominant COSMIC mutational signatures 3 and 8 (associated with homologous recombination DNA repair defect; HRD; Supplementary Table S3)^26,27^. The aging signatures 1 and 5 were dominant in 34% (13/38) of MBCs, one case displayed a dominant APOBEC signature 2 (3%, 1/38) and one harbored a dominant signature 17 of unknown etiology (3%, 1/38). No statistically significant differences were observed in the frequency of mutational signatures between *TERT* altered and *TERT* wild-type MBCs (*p* > 0.05; Fisher’s exact test; Fig. 3a; Supplementary Table S3). MBCs harboring *TERT* genetic alterations (*n* = 5) displayed a lower fraction of the genome altered (FGA, median 22%; 9–51%) than MBCs lacking genetic alterations affecting *TERT* (median = 54%; range, 20–86%, *p* = 0.002, Mann–Whitney *U* test; Supplementary Table S1 and Supplementary Fig. S2b). Nonetheless, the patterns of gene copy number profiles of both groups were comparable (Fig. 3b, c).

## Discussion

The genomic and transcriptomic diversity of MBCs has been documented by our group and others^4–11^. In fact, the repertoire of genetic alterations and transcriptomic features of MBCs appear to vary according to the predominant metaplastic component, consistent with the notion of likely genotypic–phenotypic correlations in these cancers. Here we demonstrate that in agreement with the results by Krings and Chen^12^ at variance with other forms of triple-negative breast cancer, *TERT* promoter hotspot mutations and gene amplification are found in substantial subset of MBCs (17%), and that these alterations are less frequently found in MBCs with a predominant chondroid component.

Previous studies^7–9,12,13^ have shown that *TP53* and *PIK3CA* genes are the two most frequently mutated known cancer genes in MBC and that these mutations, however, vary in frequency according to the predominant metaplastic component. Consistent with previous observations^7–9,13^, the MBCs with predominant chondroid metaplasia included in this study lacked mutations affecting *PIK3CA* and Ras pathway genes, whereas *TP53* mutations were found to be less frequent in MBCs with predominant spindle cell component compared to squamous and chondroid MBCs. These findings support the notion that a subset of MBCs harboring *PIK3CA* mutations may benefit from therapies targeting the PI3K/AKT/mTOR pathway. Recent studies have investigated the addition of PI3K/mTOR inhibitors to standard chemotherapy^28–31^, and found that patients with PI3K pathway-altered advanced triple-negative MBCs had significantly higher response rates when treated with mTOR inhibitors (temsirolimus or everolimus) in combination with liposomal doxorubicin and bevacizumab than patients with MBCs lacking PI3K/mTOR pathway alterations^31^. Given the enrichment of *PIK3CA* mutations in non-chondroid MBCs, these findings have further implications for the targeted treatment of specific histological subtypes of MBCs.

The *TERT* promoter hotspot mutations and *TER*T gene amplification described here were inversely correlated with *TP53* mutations in a subset of MBCs analyzed, and significantly associated with *PIK3CA* hotspot mutations. It should be noted that pathogenic mutations affecting *TP53* and *TERT* promoter hotspot mutations have also been found to be inversely correlated in other cancer types^16,32^, whereas *TERT* promoter and *PIK3CA* hotspot mutations have been shown to be mutually exclusive in ovarian cancers^33^, but to co-occur in other cancer types^34,35^, including breast cancer^19^. Whether these associations reflect epistatic interactions between *TERT, TP53*, and *PIK3CA* or whether they would result from the different prevalence of *TERT* alterations in different subtypes of MBC warrant further investigation.

*TERT* promoter hotspot mutations and *TERT* gene amplification have been reported in phyllodes tumors of the breast, suggesting that these genetic alterations might be the drivers of the progression from benign to malignant lesions in a subset of patients^15,22,36^. In addition, we have previously demonstrated that *TERT* somatic genetic alterations in 13% of breast adenomyoepitheliomas and in the carcinomas originating in association with or from these tumors^24^. The *TERT* promoter mutations detected in the present study are the result of an exchange of a single cytosine to a thymine at chromosome 5 base position 1,295,228 (C228T, c.-124 C > T), which results in a new binding motif for ETS transcription factors and leads to an increased transcriptional activity of the *TERT* promoter^37,38^. These mutations have been shown to constitute a mechanism of upregulated telomerase and to result in increased proliferative capacity and other oncogenic properties^39^. The frequency of *TERT* somatic alterations (i.e. in 17% of MBCs) reported here is consistent with that reported by Krings and Chen^12^ (i.e. 25% of MBCs), who observed an enrichment of *TERT* promoter mutations in MBCs with predominant spindle cell and/or squamous components. In contrast to the observations by Krings and Chen^12^, who reported the absence of *TERT* promoter mutations in chondroid matrix-producing carcinomas, in our study, *TERT* genetic alterations were identified in two cases displaying minor areas of chondroid differentiation, including one MBC with predominant spindle cell component and another MBC with predominant osseous component. It is possible that these discrepancies might be related to the fact that our series included MBCs with mixed histologic subtypes, in contrast to Krings and Chen^12^, who included only 3 MBCs with mixed components. Of these 3 cases, two had only one of the components (osseous) subjected to sequencing. The remaining cases included in their study^12^ were categorized as pure matrix-producing, spindle, squamous, or spindle/squamous MBCs that did not display differentiation along other metaplastic lineages.

The observations reported here as well as those made by others^12^ have diagnostic and taxonomic implications. First, given that *TERT* promoter hotspot mutations and gene amplification can also be found in MBCs, their detection should be used with caution in the differential diagnosis between MBC and malignant phyllodes tumor of the breast. Second, *TERT* and *HRAS* mutations have been shown to be vanishingly rare in primary breast cancers, including those of triple-negative phenotype; however, these alterations can be found in adenomyoepitheliomas and in a subset of MBCs, suggesting a tantalizing hypothesis that a subset of MBCs may evolve through similar genetic pathways or be etiologically related to adenomyoepithelial tumors. Further studies to investigate whether a subset of MBCs may constitute malignant myoepithelial tumors are warranted.

Our study has important limitations. Given the rarity of these tumors, the small sample size of the study and the limited amounts of DNA available for sequencing analysis in some cases, not all samples could be subjected to WES or MSK-IMPACT sequencing. Due to this limitation, our estimation of the frequency of *TERT* gene amplification is conservative as we cannot rule out the presence of *TERT* gene amplification in the 12 of the 16 MBCs subjected to Sanger sequencing that were *TERT* wild-type. Second, despite the multi-institutional cohort included in this study, we currently cannot define whether the mutually exclusive nature of *TERT* somatic genetic alterations and *TP53* mutations in MBCs are derived from epistatic interactions between these genes in the context of MBC or if this mutual exclusivity is solely the result of the different frequencies of alterations affecting these genes in MBCs with distinct types of predominant metaplastic components. Hence, these observations should be interpreted with caution and warrant further investigation in larger series of MBCs. Furthermore, the multi-institutional nature of our study precludes a definitive survival analysis due to the lack of clinical follow-up information in a large subset of cases in this series. Further studies to assess survival correlations with *TERT* genetic alterations in MBCs patients are warranted. Despite these limitations, our study provides evidence suggesting that *TERT* genetic alterations may play a role in MBCs and that these are likely associated with specific subsets of the disease, emphasizing the diversity and molecular heterogeneity of MBCs.

## Methods

### Subjects and samples

Following approval by the Institutional Review Board (IRB) of Memorial Sloan Kettering Cancer Center (MSKCC), a retrospective series of 60 primary MBCs was selected to be included in this study. Patient consents were obtained according to the approved IRB protocol. Cases were reviewed by at least two of four pathologists (MV, FP, HZ, and/or JSR-F) following the criteria put forward by the World Health Organization^3^. Clinicopathologic characteristics, including age, tumor size and hormone-receptor status, were retrieved from the medical records (Supplementary Table S1). Tumors were graded according to the Nottingham grading system^40^. The tumor cell content and composition of the metaplastic elements were estimated (i.e., squamous cell, spindle cell, chondroid and osseous) and in each case, the metaplastic component most abundantly present was defined as described^8^ (Supplementary Table S1). All samples were anonymized prior to tissue processing.

### Tissue preparation and DNA extraction

Ten-to-15 8-µm-thick sections from representative formalin-fixed paraffin embedded (FFPE) tumor and matched normal tissue blocks of 21 MBCs (21/60) were stained with nuclear fast red and microdissected using a sterile needle under a stereomicroscope (Olympus SZ61) to ensure a tumor content >80%, as previously described^41^. Genomic DNA was extracted from tumor and matched normal tissues using the DNeasy Blood and Tissue Kit (Qiagen) according to manufacturers’ instructions.

### Whole-exome sequencing and MSK-IMPACT sequencing

Out of 60 MBCs included in our cohort, 44 (73%) were subjected to WES (*n* = 27) or to massively parallel sequencing targeting all coding regions of 341 to 468 cancer-related genes using the FDA-approved MSK Integrated Mutation Profiling of Actionable Cancer Targets assay (MSK-IMPACT, *n* = 17, Supplementary Table S2)^42^. Of the 27 MBCs subjected to WES, five MBCs were microdissected and subjected to WES at MSK’s Integrated Genomics Operations (IGO) using validated protocols, as previously described^7,43^, and for 22 MBCs the raw sequencing data (FASTQ files) reported in Ng et al.^7^ were retrieved and reanalyzed (see below). Of the 17 MBCs subjected to MSK-IMPACT sequencing, three were previously reported in Zehir et al.^14^. Hence, in this manuscript, we include massively parallel sequencing data from 19 previously unreported MBCs (5 subjected to WES and 14 to targeted MSK-IMPACT sequencing) as well as Sanger sequencing data from 16 previously unreported MBCs (see below). WES and MSK-IMPACT sequencing data were processed using our validated bioinformatics pipeline^16,43,44^. In brief, sequence reads were aligned to the reference human genome GRCh37 using the Burrows-Wheeler Aligner (BWA v0.7.15)^45^. Somatic single nucleotide variants (SNVs) were detected with MuTect (v1.0)^46^. Insertion and deletions (indels) were detected using Strelka (v2.0.15)^47^, VarScan2 (v2.3.7)^48^, Platypus (v0.8.1)^49^, Lancet (v1.0.0)^50^, and Scalpel (v0.5.3)^51^. Cancer cell fractions (CCFs) of each somatic mutation were computed using ABSOLUTE (v1.0.6)^52^, as previously described^43,53^. Copy number alterations (CNAs) and loss of heterozygosity were determined using FACETS^54^. Somatic mutations in tumor suppressor genes that were deleterious/loss-of-function or targeting a mutational hotspot in oncogenes were considered pathogenic. Mutations targeting hotspot loci were annotated using cancerhotspots.org^55^. Mutational signatures were defined using Signature Multivariate Analysis (SigMA) tool^56^, for cases with at least 5 SNVs, as previously described^57^. Exposure-based dominant mutational signatures obtained by SigMA^56^ (Supplementary Table S3) were comparable to the mutational signatures reported by Ng et al.^7^, which were inferred using DeconstructSigs^58^, in 68% (15/22) of the MBCs.

Tumour mutation burden (TMB) was calculated as the total number of non-synonymous mutations divided by the number of bases analyzed, per megabase. The fraction of genome altered (FGA) was defined as the cumulative size of copy number segments which are not copy neutral divided by the cumulative size of all copy number segments, as previously described^14,59^.

As part of an exploratory, hypothesis generating analysis, the repertoire of non-synonymous somatic mutations, mutational frequencies, and copy number alterations of MBCs harboring *TERT* somatic genetic alterations, including promoter mutations and gene amplification, were compared to MBCs lacking *TERT* genetic alterations. For the comparative analyses of the repertoire of non-synonymous somatic mutations, mutational frequencies, and copy number alterations of MBCs subjected to either WES or MSK-IMPACT, genes were restricted to the 341 genes included in MSK-IMPACT.

### Assessment of somatic mutations by Sanger sequencing

We conducted the assessment of *TERT* promoter hotspot loci, *TP53* (exons 2 to 11), *PIK3CA* (exons 9 and 20), *HRAS* (exon 3), and *BRAF* (exons 11 and 15) hotspot mutations in 16 MBCs with insufficient DNA yield by Sanger sequencing. In addition, as *TERT* promoter region is usually poorly covered by exome sequencing, *TERT* promoter hotspot mutations were assessed by Sanger sequencing in the 27 MBCs subjected to WES. PCR amplification of the selected genes was performed using the AmpliTaq Gold 360 Master Mix kit (Life Technologies, ThermoFisher Scientific) using previously described primers^16,24,60^ (Supplementary Table S4). PCR fragments were cleaned using ExoSAP It (ThermoFisher Scientific) and Sanger sequenced as previously described^15,16^.

### Statistical analysis

Fisher’s exact test and Chi-Square test were used for comparison of categorical variables, and Mann–Whitney *U* test for continuous variables. All tests were two-tailed and *p* values <0.05 were considered statistically significant. A mutual exclusivity analysis was performed using combinations of mutually exclusive alterations (CoMEt) with the use of a pair-wise Fisher’s exact test to detect the presence of significant pairs of genes^25^.

### Reporting summary

Further information on research design is available in the Nature Research Reporting Summary linked to this article.

## Supplementary information

Supplementary Information

Reporting Summary

## Data Availability

“The data generated and analysed during this study are described in the following data record: 10.6084/m9.figshare.14160482^61^. The whole-exome sequencing data supporting Figs. 2, 3, Supplementary Figs. S2 and S3, and Supplementary Tables S1, S2, and S3 are openly available in the Sequence Read Archive via the following accession: https://identifiers.org/ncbi/insdc.sra:SRP073692^62^. These data were first described in the original publication by Ng et al.^7^ MSK-IMPACT sequencing data of 3 samples included in the MSK-IMPACT Clinical Sequencing Cohort supporting Figs. 2, 3, Supplementary Figs. S2, S3, and Supplementary Tables S1 and S2 are publicly available at cBioPortal (https://identifiers.org/cbioportal:msk_impact_2017^63^). These data were first described in the original publication by Zehir et al.^14^ Sequencing data of 19 previously unreported MBCs (5 subjected to whole-exome sequencing and 14 to MSK-IMPACT sequencing) are available at cBioPortal (https://identifiers.org/cbioportal:mbc_msk_2021^64^). Additionally, the following data are available upon request from the corresponding authors: Histologic images supporting Fig. 1 and Supplementary Fig. S1; Sanger sequencing electropherograms supporting Fig. 2, Table 2, and Supplementary Figs. S1 and S4; Clinicopathologic data supporting Figs. 2 and 3, Table 1, and Supplementary Table S1.”
