# Supplementary material for: TERT promoter hotspot mutations and gene amplification in metaplastic breast cancer
Source: NPJ Breast Cancer. 2021 Apr 16;7:43. doi: 10.1038/s41523-021-00250-8 (PMC8052452; doi:10.1038/s41523-021-00250-8)
Supplement: Supplementary file 1 — Supplementary Information [file 41523_2021_250_MOESM1_ESM.pdf]

SUPPLEMENTARY MATERIALS

TERT promoter hotspot mutations and gene amplification in metaplastic breast cancer

da Silva et al.

Supplementary Figures S1 – S4

Supplementary Tables S1 – S4

Supplementary Figure S1

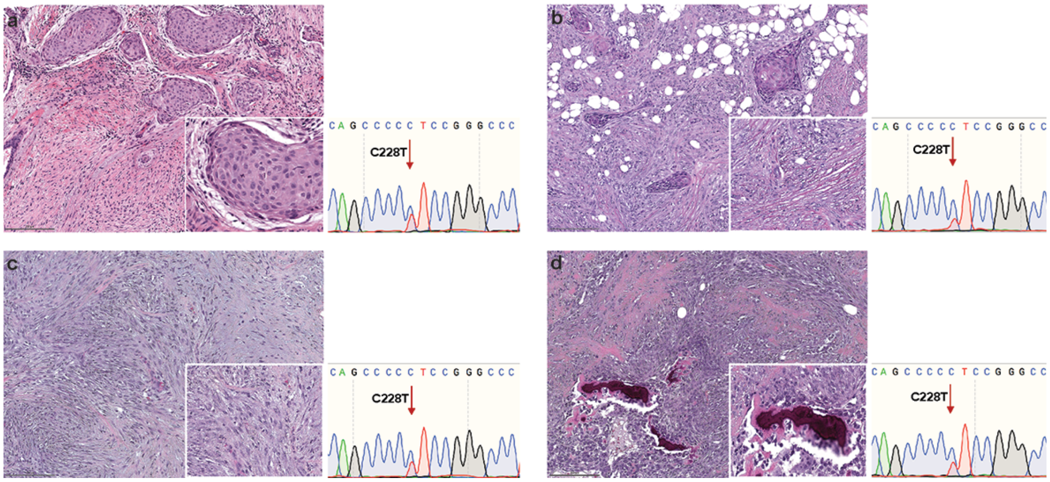

Supplementary Figure S1. *TERT* promoter mutations identified by Sanger sequencing in metaplastic breast cancers. Representative hematoxylin-and-eosin photomicrographs of metaplastic breast cancers and corresponding representative Sanger sequencing electropherograms of *TERT* promoter hotspot loci. (**a**) Metaplastic squamous cell carcinoma (MBC110T), (**b-c**) Metaplastic spindle cell carcinoma (MT59 and MBC119T, respectively), and (**d**) Metaplastic carcinoma with osseous differentiation (MBC120T). Scale bars, 200 μM.

Supplementary Figure S2

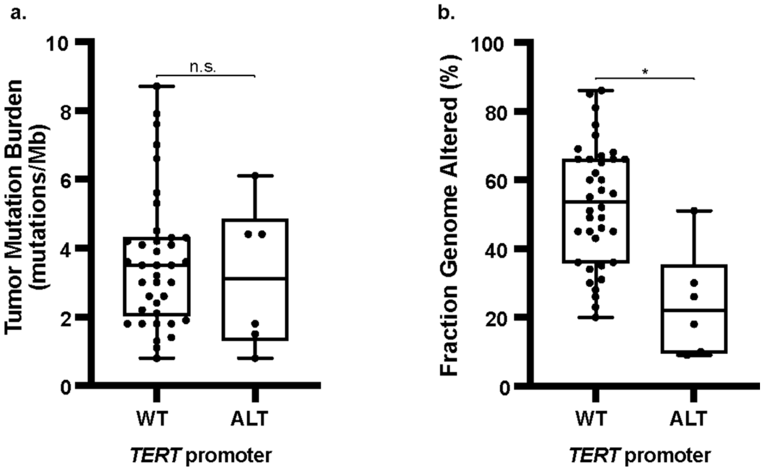

Supplementary Figure S2. Tumor mutation burden (TMB) and fraction of genome altered (FGA) in metaplastic breast carcinomas harboring *TERT* genetic alterations (promoter mutation and gene amplification) and *TERT* promoter wild-type metaplastic breast carcinomas. The box-and-whisker plots compare (**a**) tumor mutation burden and (**b**) fraction of genome altered (FGA) frequency between *TERT* promoter altered (ALT; n=6) and *TERT* wild-type (WT; n=38) metaplastic breast cancers. Minimum, maximum and median values, with the 75th and 25th percentiles are displayed. n.s., not significant; **p*=0.002, Mann-Whitney *U* Test.

Supplementary Figure S3

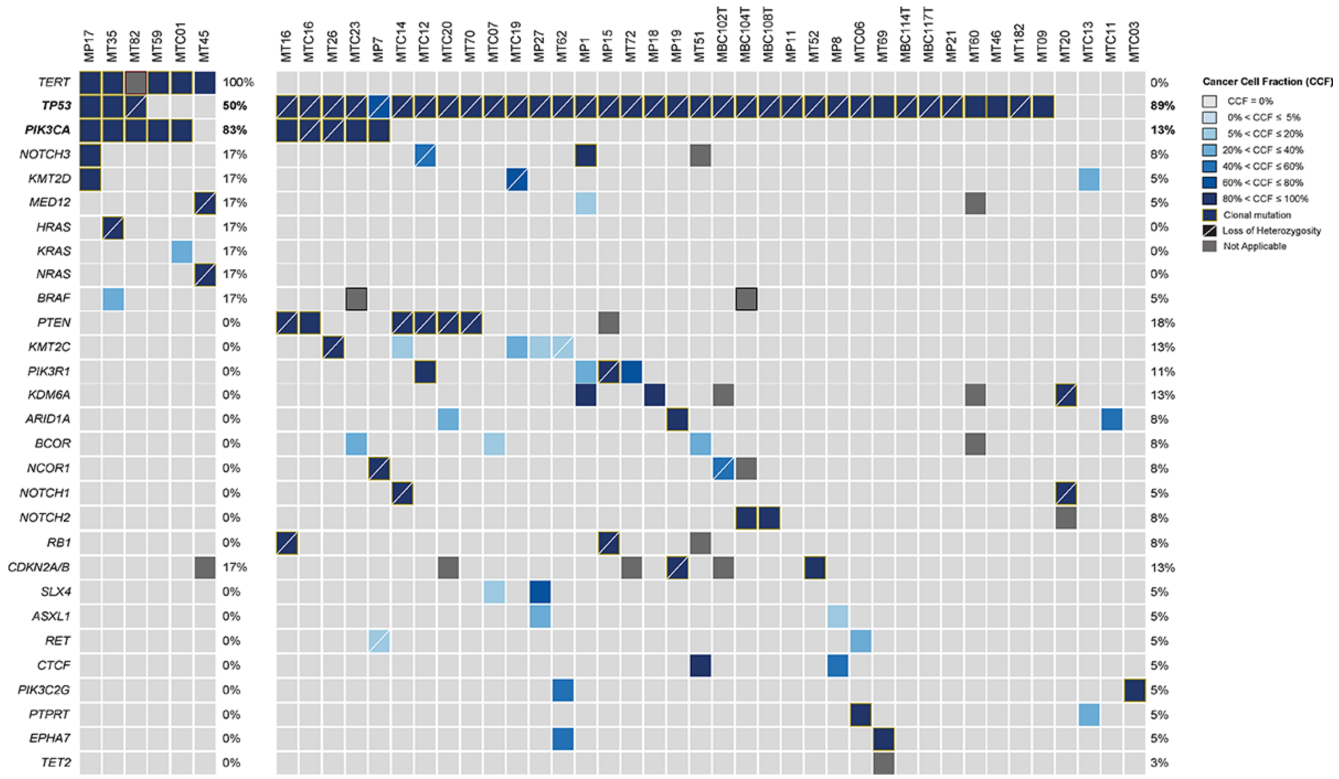

Supplementary Figure S3. Cancer cell fractions of mutations identified in metaplastic breast cancers. Cancer cell fractions and clonality of non-synonymous somatic mutations identified in metaplastic breast cancers harboring *TERT* genetic alterations (*TERT* promoter hotspot mutations, n=5; *TERT* gene amplification, n=1; left) and *TERT* wild-type (n=38, right), by whole-exome sequencing or MSK-IMPACT targeted sequencing. Cancer cell fractions are color-coded according to the legend. Clonal mutations are depicted by a yellow box.

Supplementary Figure S4

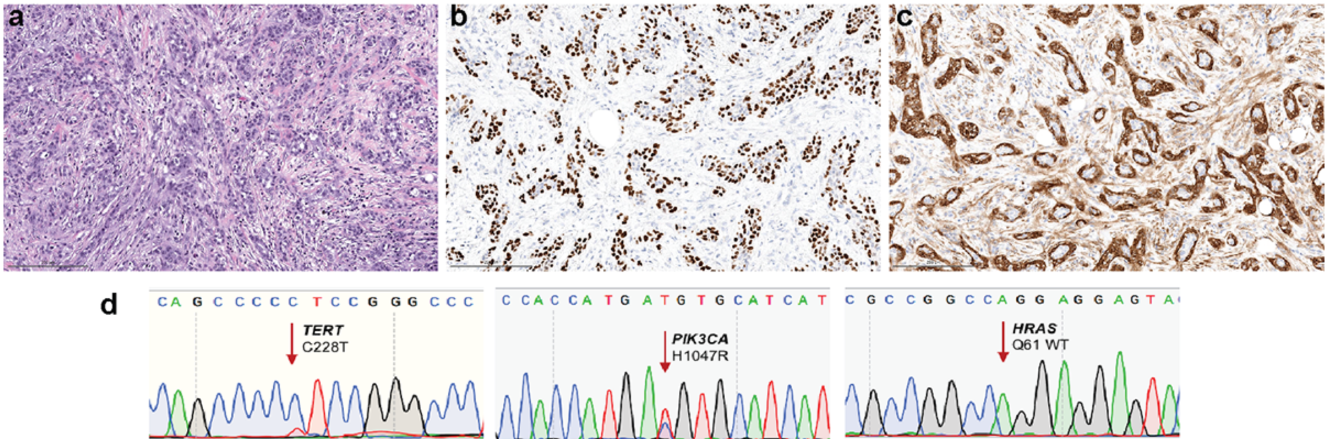

Supplementary Figure S4. Metaplastic breast carcinoma displaying features consistent with the presence of an adenomyoepithelioma. Metaplastic squamous cell carcinoma with focal adenosquamous morphology (MBC103T) displaying foci with features consistent with a diagnosis of adenomyoepithelioma in the adjacent areas. (**a**) Representative hematoxylin-and-eosin photomicrographs, and positive staining of (**b**) p63 and (**c**) calponin, by immunohistochemistry. (**d**) Corresponding sequencing electropherograms of hotspot mutations targeting *TERT* promoter loci (C228T), *PIK3CA* (H1047R) and *HRAS* (WT), identified by Sanger sequencing. Scale bars, 200 μM. WT, wild-type.

Supplementary Table S1. Clinicopathologic features and mutation status of *TERT* promoter, *TP53, PIK3CA, HRAS* and *BRAF* in 60 metaplastic carcinomas of the breast.

Sample ID	Sequencing method	Diagnosis	Matrix Producing	Predominant Histologic Component	Squamous Component (%)	Spindle Component (%)	Osseous Component (%)	Chondroid Componen t (%)	Epithelial Component (%)	DCIS	ER	PR	HER2	Age at diagnosis (years)	Tumor size (cm)	Tubule Formation	Nuclear Grade	Mitosis	Histologic Grade	*TERT* genetic alteration (promoter mutation and amplification)	*TP53* mutations (exons 2 to 11)	*TP53* Allele Frequency	Clonality	LOH	Cancer Cell Fraction	Fraction Genome Altered	*PIK3CA* mutation (exon 9 and 20)	*HRAS* mutation (exon 3)	*BRAF* alteration (exons 11 and 15 mutation and amplification)	Tumor mutation burden (mutations/Mb)	Tumor purity %	Dominant mutational signature (SigMA)	Samples (primary or metastatic tumor)	Biopsy (B) or resection (R) specimen	Neoadjuvant treatment
MP1	WES / Sanger	Metaplastic carcinoma with mesenchymal differentiation	Y	Chondroid	0	0	0	60	40	Present	Neg	Neg	Neg	50	1.4	3	3	3	3	WT	R110P	0.32	clonal	LOH	0.9	0.307	WT	WT	WT	4.2	52	HRD	Primary	R	No
MP11	WES / Sanger	Carcinoma with squamous metaplasia	N	Squamous	85	10	0	5	0	Present	Neg	Neg	Neg	37	2.2	3	3	3	3	WT	C229Yfs*10	0.35	clonal	LOH	1.0	0.690	WT	WT	WT	7.9	40	HRD	Primary	R	No
MP15	WES / Sanger	Metaplastic carcinoma with mesenchymal differentiation	Y	Chondroid	0	0	0	30	70	Present	Neg	Neg	Neg	63	2.7	2	3	3	3	WT	G112Afs*11	0.43	clonal	LOH	1.0	0.507	WT	WT	WT	4.1	40	HRD	Primary	R	No
MP17	WES / Sanger	Metaplastic spindle cell carcinoma	N	Spindle	5	95	0	0	0	Present	Neg	Neg	Neg	66	14.0	3	3	3	3	C228T	V173L<br>X225_splice	0.27<br>0.14	clonal<br>subclonal	-	0.87<br>0.47	0.09	H1047R	WT	WT	1.5	25	HRD	Primary	R	No
MP18	WES / Sanger	Metaplastic carcinoma with mesenchymal differentiation	N	Chondroid	0	0	0	30	70	Present	Neg	Neg	Neg	39	1.7	3	3	3	3	WT	I195F	0.40	clonal	LOH	1.0	0.523	WT	WT	WT	3.0	90	HRD	Primary	R	No
MP19	WES / Sanger	Metaplastic carcinoma with mesenchymal differentiation	Y	Chondroid	0	0	0	80	20	Present	Neg	Neg	Neg	52	1.3	3	3	3	3	WT	T123Dfs*26	0.52	clonal	LOH	1.0	0.573	WT	WT	WT	4.3	36	HRD	Primary	R	No
MP21	WES / Sanger	Metaplastic spindle cell carcinoma	N	Spindle	0	90	0	0	10	Present	Neg	Neg	Neg	37	2.4	3	3	3	3	WT	V173G	0.73	subclonal	LOH	1.0	0.847	WT	WT	WT	2.4	67	HRD	Primary	R	No
MP27	WES / Sanger	Metaplastic carcinoma with mesenchymal differentiation	Y	Chondroid	0	0	0	70	30	N/I	Neg	Neg	Neg	57	1.1	2	3	3	3	WT	C176F	0.52	clonal	LOH	1.0	0.858	WT	WT	WT	3.6	51	HRD	Primary	R	No
MP7 *	WES / Sanger	Metaplastic spindle cell carcinoma	N	Spindle	30	60	0	0	0	Present	Neg	Neg	Neg	51	2.5	3	3	3	3	WT	C135Afs*35	0.49	subclonal	LOH	0.8	0.597	H1047R	WT	WT	3.0	79	HRD	Primary	R	No
MP8	WES / Sanger	Metaplastic spindle cell carcinoma	N	Spindle	0	90	0	0	10	N/I	Neg	Neg	Neg	34	2.9	3	3	2	3	WT	R248Q	0.31	clonal	LOH	0.9	0.663	WT	WT	WT	4.1	17	HRD	Primary	R	No
MTC01	WES / Sanger	Metaplastic spindle cell carcinoma	N	Spindle	0	100	0	0	0	Present	Neg	Neg	Neg	67	3.6	3	3	2	3	C228T	WT	-	-	-	-	0.10	H1047R	WT	WT	0.8	42	HRD	Primary	R	No
MTC03	WES / Sanger	Metaplastic spindle cell carcinoma	N	Spindle	0	100	0	0	0	Present	Neg	Neg	Neg	43	3.2	3	3	3	3	WT	WT	-	-	LOH	-	0.618	WT	WT	WT	8.7	54	HRD	Primary	R	No
MTC06	WES / Sanger	Metaplastic carcinoma with mesenchymal differentiation	Y	Chondroid	0	0	0	50	50	Present	Neg	Neg	Neg	53	1.0	2	3	2	2	WT	R273H	0.61	clonal	LOH	1.0	0.664	WT	WT	WT	6.6	59	HRD	Primary	R	No
MTC07	WES / Sanger	Metaplastic carcinoma with mesenchymal differentiation	Y	Chondroid	0	0	0	30	70	Present	Neg	Neg	Neg	60	2.2	3	3	3	3	WT	H193L<br>I195T	H193L= 0.42<br>I195T= 0.04	clonal<br>subclonal	LOH	1<br>0.19	0.449	WT	WT	WT	7.6	48	Aging	Primary	R	No
MTC11	WES / Sanger	Metaplastic carcinoma with mesenchymal differentiation	Y	Chondroid	0	0	0	30	70	N/I	Neg	Neg	Neg	63	4.0	3	2	2	3	WT	WT	-	-	LOH	-	0.809	WT	WT	WT	3.2	28	HRD	Primary	R	No
MTC12	WES / Sanger	Metaplastic squamous cell carcinoma	N	Squamous	80	0	0	0	20	Present	Neg	Neg	Neg	62	3.5	3	3	1	2	WT	R306*	0.35	clonal	LOH	1.0	0.342	WT	WT	WT	2.1	40	HRD	Primary	R	No
MTC13	WES / Sanger	Metaplastic carcinoma with mesenchymal differentiation	Y	Chondroid	0	0	0	50	50	Present	Neg	Neg	Neg	47	4.0	3	3	1	2	WT	WT	-	-	LOH	-	0.458	WT	WT	WT	1.4	71	Aging	Primary	R	No
MTC14	WES / Sanger	Metaplastic carcinoma with mesenchymal differentiation	Y	Chondroid	0	0	0	15	85	Present	Neg	Neg	Neg	35	3.1	3	3	3	3	WT	I195T	0.79	clonal	LOH	1.0	0.553	WT	WT	WT	4.2	47	HRD	Primary	R	No
MTC16	WES / Sanger	Metaplastic squamous cell carcinoma	N	Squamous	90	5	0	0	5	N/I	Neg	Neg	Neg	54	3.2	3	3	3	3	WT	V274A	0.27	clonal	LOH	1.0	0.664	C420R	WT	WT	0.8	37	Aging	Primary	R	No
MTC19	WES / Sanger	Metaplastic carcinoma with mesenchymal differentiation	Y	Chondroid	10	0	20	60	10	N/I	Neg	Neg	Neg	64	3.0	3	3	3	3	WT	W146*	0.55	clonal	LOH	1.0	0.493	WT	WT	WT	1.3	59	Aging	Primary	R	No
MTC20	WES / Sanger	Metaplastic squamous cell carcinoma	N	Squamous	40	0	0	20	40	Present	Neg	Neg	Neg	78	3.0	3	3	2	3	WT	P190Lfs*57	0.40	clonal	LOH	1.0	0.731	WT	WT	WT	3.0	44	Aging	Primary	R	No
MTC23	WES / Sanger	Metaplastic squamous cell carcinoma	N	Squamous	100	0	0	0	0	Present	Neg	Neg	Pos	49	12.0	3	3	3	3	WT	X332_splice	0.61	clonal	LOH	1.0	0.650	E542K	WT	Ampl	1.9	65	HRD	Primary	R	No
MBC102T	WES / Sanger	Metaplastic carcinoma with mesenchymal differentiation	Y	Chondroid	0	0	0	90	10	Present	Neg	Neg	Neg	48	3.0	3	3	3	3	WT	Q192*	0.45	clonal	LOH	1.0	0.661	WT	WT	Ampl	2.2	60	HRD	Primary	R	No
MBC104T	WES / Sanger	Metaplastic carcinoma with mesenchymal differentiation	Y	Chondroid	0	0	0	70	30	Present	Neg	Neg	Neg	52	1.1	3	3	3	3	WT	G108fs	0.4	clonal	LOH	1.0	0.677	WT	WT	WT	3.6	50	HRD	Primary	R	No
MBC108T	WES / Sanger	Metaplastic carcinoma with mesenchymal differentiation	Y	Chondroid	0	0	0	90	10	N/I	Neg	Neg	Neg	75	3.0	3	3	2	3	WT	R248Q	0.63	clonal	LOH	1.0	0.760	WT	WT	WT	3.9	40	Aging	Primary	R	No
MBC114T	WES / Sanger	Metaplastic carcinoma with mesenchymal differentiation	Y	Chondroid	0	0	0	90	10	Present	Neg	Neg	Neg	49	1.4	3	3	3	3	WT	X187_splice	0.28	clonal	LOH	1.0	0.449	WT	WT	WT	1.8	70	Aging	Primary	R	No
MBC117T	WES / Sanger	Metaplastic carcinoma with mesenchymal differentiation	Y	Chondroid	0	0	0	70	30	Present	Neg	Neg	Neg	57	1.6	3	3	3	3	WT	C242G	0.91	clonal	LOH	1.0	0.667	WT	WT	WT	4.3	80	Aging	Primary	R	No
MT82	MSK-IMPACT	Metaplastic carcinoma with mesenchymal differentiation	Y	Osseous	0	0	50	30	20	Present	Neg	Neg	Neg	62	2.1	3	3	3	3	AMPL	R248Q	0.63	clonal	LOH	1.0	0.509	P104_V105delinsL	WT	WT	4.4	60	Aging	Primary	R	No
MT70	MSK-IMPACT	Metaplastic carcinoma with mesenchymal differentiation	Y	Osseous	0	0	70	5	25	N/I	Neg	Neg	Neg	68	2.9	3	3	3	3	WT	S94*	0.75	clonal	LOH	1.0	0.359	WT	WT	WT	2.6	70	-	Primary	R	Yes
MT62	MSK-IMPACT	Metaplastic carcinoma with mesenchymal differentiation	Y	Chondroid	0	0	5	60	35	N/I	Neg	Neg	Neg	64	2.5	3	3	3	3	WT	R342*	0.47	clonal	LOH	1.0	0.448	WT	WT	WT	5.3	30	HRD	Primary	R	No
MT20	MSK-IMPACT	Metaplastic carcinoma with mesenchymal differentiation	Y	Chondroid	0	0	0	80	20	Present	Neg	Neg	Neg	62	5.8	3	2	1	2	WT	WT	-	-	-	-	0.353	WT	WT	WT	3.5	70	Aging	Primary	R	No
MT60	MSK-IMPACT	Metaplastic carcinoma with mesenchymal differentiation	Y	Chondroid	0	0	0	80	20	Present	Neg	Neg	Neg	46	3.2	3	3	3	3	WT	I232Sfs*15	0.16	clonal	-	0.9	0.555	WT	WT	WT	4.5	40	HRD	Primary	R	No
MT182	MSK-IMPACT	Metaplastic spindle cell carcinoma	N	Spindle	0	90	0	0	10	Present	Neg	Neg	Neg	66	1.8	3	3	2	3	WT	R175H	0.46	clonal	LOH	1.0	0.261	WT	WT	WT	1.1	35	-	Primary	R	No
MT16	MSK-IMPACT	Metaplastic spindle cell carcinoma	N	Spindle	15	45	0	5	35	Present	Neg	Neg	Neg	70	5.2	3	3	3	3	WT	C238Y	0.74	clonal	LOH	1.0	0.601	H1047R	WT	WT	7.0	70	APOBEC	Primary	R	Yes
MT52	MSK-IMPACT	Metaplastic squamous cell carcinoma	N	Squamous	95	0	0	0	5	Present	Neg	Neg	Neg	40	6.5	3	3	3	3	WT	Y220*	0.27	clonal	LOH	1.0	0.201	WT	WT	WT	5.6	20	-	Primary	R	No
MT35	MSK-IMPACT	Metaplastic squamous cell carcinoma	N	Squamous	70	30	0	0	0	N/I	Neg	Neg	Neg	46	2.3	3	3	3	3	C228T	V173L<br>E204Vfs*4	0.10<br>0.25	subclonal<br>clonal	-	0.4<br>0.95	0.26	H1047R / E542Q	Q61R	D594N	6.1	40	Aging	Primary	R	No
MT26	MSK-IMPACT	Metaplastic squamous cell carcinoma	N	Squamous	80	0	0	0	20	N/A	N/A	N/A	N/A	52	N/A	3	3	3	3	WT	V274D	0.85		LOH		0.485	E545K	WT	WT	3.5	40	HRD	Primary	B	N/A
MT59	MSK-IMPACT	Metaplastic spindle cell carcinoma	N	Spindle	10	85	0	0	5	Present	Neg	Neg	Neg	75	1.7	3	3	3	3	C228T	WT	-	-	-	-	0.18	H1065Lfs*8	WT		1.8	20	-	Primary	R	No
MT72	MSK-IMPACT	Metaplastic squamous cell carcinoma	N	Squamous	90	0	0	0	10	Present	Neg	Neg	Neg	35	4.5	3	3	3	3	WT	P80Lfs*43	0.51	clonal	LOH	1.0	0.295	WT	WT	WT	1.8	50	-	Primary	R	Yes
MT69	MSK-IMPACT	Metaplastic spindle cell carcinoma	N	Spindle	0	100	0	0	0	N/I	Neg	Neg	Neg	43	3.9	3	3	3	3	WT	R248Q	0.48	clonal	-	2.0	0.431	WT	WT	WT	3.5	60	Signature 17	Primary	R	Yes
MT46	MSK-IMPACT	Metaplastic squamous cell carcinoma	N	Squamous	80	0	0	0	20	Present	Neg	Neg	Neg	47	3.1	3	3	3	3	WT	G266E	0.52		-		0.362	WT	WT	WT	1.8	40	Aging	Primary	R	Yes
MT09	MSK-IMPACT	Metaplastic carcinoma with mesenchymal differentiation	Y	Chondroid	0	0	0	90	10	Present	Neg	Neg	Neg	73	2.2	3	3	3	3	WT	E298*<br>X307_splice	E298*= 0.28<br>X307_splice= 0.29	clonal<br>clonal	-	E298*= 0.98<br>X307_splice= 1.0	0.225	WT	WT	WT	1.8	30	-	Primary	B	No
MT45	MSK-IMPACT	Metaplastic spindle cell carcinoma	N	Spindle	0	100	0	0	0	N/A	Neg	Neg	Neg	54	N/A	3	2	3	3	C228T	WT	-	-	-	-	0.30	WT	WT	WT	4.4	70	HRD	Primary	B	No
MT51	MSK-IMPACT	Metaplastic carcinoma with mesenchymal differentiation	Y	Chondroid	0	0	0	80	20	Present	Neg	Neg	Neg	75	3	3	3	3	3	WT	R273H	0.41				0.281	WT	WT	WT	2.6	40	Aging	Primary	B	Yes
MBC101T	Sanger	Metaplastic spindle cell carcinoma	N	Spindle	0	60	0	0	40	Present	Neg	Neg	Neg	60	1.3	3	3	3	3	WT	P47fs	-	-		-	-	WT	WT	WT	-	-	-	Primary	R	No
MBC103T	Sanger	High grade adenosquamous/Metaplastic Squamous cell carcinoma	N	Squamous	20	0	0	0	80	Present	Neg	Neg	Neg	70	3.2	3	3	2	3	C228T	WT	-	-	-	-	-	H1047R	WT	WT	-	-	-	Primary	R	No
MBC105T	Sanger	Metaplastic carcinoma with mesenchymal differentiation	Y	Chondroid	0	0	0	50	50	Present	Neg	Neg	Neg	59	1.2	3	3	3	3	WT	R213*	-	-		-	-	WT	WT	WT	-	-	-	Primary	R	No
MBC107T	Sanger	Metaplastic carcinoma with mesenchymal differentiation	Y	Osseous	0	0	40	30	30	N/I	Neg	Neg	Neg	45	4.6	3	3	3	3	WT	R273C	-	-		-	-	WT	WT	WT	-	-	-	Primary	R	No
MBC109T	Sanger	Metaplastic carcinoma with mesenchymal differentiation	Y	Osseous	0	0	30	10	60	Present	Neg	Neg	Neg	85	3.9	3	3	3	3	WT	R282W	-	-		-	-	WT	WT	WT	-	-	-	Primary	R	No
MBC110T	Sanger	Metaplastic squamous cell carcinoma	N	Squamous	90	0	0	0	10	N/I	Neg	Neg	Neg	72	2.5	3	3	2	3	C228T	WT	-	-	-	-	-	H1047R	WT	WT	-	-	-	Primary	R	No
MBC111T	Sanger	Metaplastic carcinoma with mesenchymal differentiation	Y	Chondroid	0	0	0	60	40	Present	Neg	Neg	Neg	82	1.7	3	3	2	3	WT	R280G	-	-		-	-	WT	WT	WT	-	-	-	Primary	R	No
MBC112T	Sanger	Metaplastic squamous cell carcinoma	N	Squamous	70	20	0	0	10	N/I	Neg	Neg	Pos	41	19.0	3	3	3	3	WT	P72fs	-	-		-	-	WT	WT	WT	-	-	-	Primary	R	No
MBC113T	Sanger	Metaplastic spindle cell carcinoma	N	Spindle	0	85	0	5	10	Present	Neg	Neg	Neg	79	1.5	3	3	3	3	WT	D281E	-	-		-	-	WT	Q61K	WT	-	-	-	Primary	R	No
MBC115T	Sanger	Metaplastic carcinoma with mesenchymal differentiation	Y	Chondroid	0	0	0	70	30	Present	Neg	Neg	Neg	82	1.8	3	3	3	3	WT	WT	-	-		-	-	WT	WT	WT	-	-	-	Primary	R	No
MBC116T	Sanger	Metaplastic spindle cell carcinoma	N	Spindle	0	60	0	5	35	Present	Neg	Neg	Neg	65	4.4	3	3	3	3	WT	R273H	-	-		-	-	WT	WT	WT	-	-	-	Primary	R	No
MBC118T	Sanger	Spindle cell carcinoma/Fibromatosis-like metaplastic carcinoma	N	Spindle	0	100	0	0	0	N/I	Neg	Neg	Neg	44	6.8	3	2	2	2	WT	WT	-	-		-	-	WT	WT	WT	-	-	-	Primary	R	No
MBC119T	Sanger	Metaplastic spindle cell carcinoma	N	Spindle	0	60	0	40	0	Present	Neg	Neg	Neg	83	5.9	3	3	3	3	C228T	WT	-	-	-	-	-	H1047R	WT	WT	-	-	-	Primary	R	No
MBC120T	Sanger	Metaplastic carcinoma with mesenchymal differentiation	N	Osseous	0	20	70	0	10	N/I	Neg	Neg	Neg	56	4.7	3	3	2	3	C228T	WT	-	-	-	-	-	H1047R	WT	WT	-	-	-	Primary	R	No
MBC121T	Sanger	Metaplastic carcinoma with mesenchymal differentiation	Y	Chondroid	0	0	5	60	35	N/I	Neg	Neg	Neg	72	4.8	3	3	3	3	WT	R175H	-	-		-	-	WT	WT	WT	-	-	-	Primary	R	No
MBC122T	Sanger	Metaplastic squamous cell carcinoma	N	Squamous	60	0	0	30	10	Present	Neg	Neg	Neg	47	1.8	3	3	3	3	WT	R213*	-	-		-	-	WT	WT	WT	-	-	-	Primary	R	No

N/I, not identified; N/A, not available; Neg, negative; Pos, positive; WT, wild-type

** MP7: displayed 10% rhabdoid cell component*

Supplementary Table S2: Non-synonymous somatic mutations identified in 44 metaplastic breast cancers subjected to MSK-IMPACT or whole-exome sequencing.

Sample ID	Chromo-some	Genomic Position	Reference Allele	Alternate Allele	Gene Symbol	Variant	Amino Acid Change	Tumor MAF	Normal MAF	Tumor Depth (x)	Normal Depth (x)	Cancer Cell Fraction	Clonal/ Subclonal Mutation	Loss of Heterozygosity	Hotspot mutation
MT52	9	21974723	C	A	CDKN2A	Missense_Mutation	p.G35V	0.13	0.00	664	386	0.79	Subclonal	-	FALSE
MT52	17	7578189	A	C	TP53	Nonsense_Mutation	p.Y220*	0.27	0.00	904	505	1	Clonal	LOH	FALSE
MT52	17	29557369	G	T	NF1	Missense_Mutation	p.D1028Y	0.23	0.00	756	233	1	Clonal	-	FALSE
MT52	9	21971162	GGAGCAGCAGC	AG	CDKN2A	In_Frame_Del	p.L63_H66delinsY	0.21	0.00	179	74	1	Clonal	-	FALSE
MT45	5	1295228	G	A	TERT	Promoter		0.27	0.00	524	338	0.83	Clonal	-	TRUE
MT45	1	115256529	T	A	NRAS	Missense_Mutation	p.Q61L	0.54	0.00	984	598	1	Clonal	LOH	TRUE
MT45	12	120784077	C	T	MSI1	Missense_Mutation	p.R303H	0.28	0.00	857	791	0.85	Subclonal	-	FALSE
MT45	7	55224232	A	G	EGFR	Missense_Mutation	p.N338S	0.35	0.00	762	474	1	Clonal	-	FALSE
MT45	23	70339169	CCCTTCCCCCTTCCCCTAAGGAAAAAACAACTAAACGCCGCTTTCCTGCCTCAGGATGAACTGACGGCCTTGAATGTAAAACAAGGTT	-	MED12	Splice_Site	p.X34_splice	0.34	0.00	492	396	1	Clonal	LOH	FALSE
MT09	17	7577046	C	A	TP53	Nonsense_Mutation	p.E298*	0.28	0.00	749	865	0.98	Clonal	-	FALSE
MT09	17	7577018	C	G	TP53	Splice_Site	p.X307_splice	0.29	0.00	675	806	1	Clonal	-	FALSE
MT51	17	7577120	C	T	TP53	Missense_Mutation	p.R273H	0.41	0.00	903	787	1	Clonal	LOH	TRUE
MT51	16	67654646	C	A	CTCF	Missense_Mutation	p.P378Q	0.34	0.00	595	507	0.82	Subclonal	-	FALSE
MT51	23	39922202	T	C	BCOR	Missense_Mutation	p.I1324V	0.13	0.00	636	514	0.32	Subclonal	-	FALSE
MT26	3	178936091	G	A	PIK3CA	Missense_Mutation	p.E545K	0.89	0.00	939	806	1	Clonal	LOH	TRUE
MT26	17	7577117	A	T	TP53	Missense_Mutation	p.V274D	0.85	0.00	392	926	1	Clonal	LOH	TRUE
MT26	7	151878887	G	A	KMT2C	Nonsense_Mutation	p.Q2020*	0.74	0.00	90	922	1	Clonal	LOH	FALSE
MT26	23	66937439	T	-	AR	Frame_Shift_Del	p.F765Sfs*24	0.43	0.00	343	955	1	Clonal	-	FALSE
MT46	17	7577141	C	T	TP53	Missense_Mutation	p.G266E	0.52	0.00	642	545	1	Clonal	-	TRUE
MT46	6	26197075	C	A	HIST1H3D	Missense_Mutation	p.R135M	0.06	0.00	640	349	0.18	Subclonal	LOH	FALSE
MT69	17	7577538	C	T	TP53	Missense_Mutation	p.R248Q	0.48	0.00	585	801	1	Clonal	-	TRUE
MT69	6	94068121	G	C	EPHA7	Missense_Mutation	p.R281G	0.30	0.00	294	205	1	Clonal	-	FALSE
MT69	8	55372044	G	C	SOX17	Missense_Mutation	p.C245S	0.27	0.00	208	246	0.77	Subclonal	LOH	FALSE
MT69	9	137293628	G	T	RXRA	Missense_Mutation	p.G60V	0.39	0.00	630	1025	1	Clonal	-	FALSE
MT16	3	178952085	A	G	PIK3CA	Missense_Mutation	p.H1047R	0.38	0.00	698	468	1	Clonal	-	TRUE
MT16	17	7577568	C	T	TP53	Missense_Mutation	p.C238Y	0.74	0.00	786	794	1	Clonal	LOH	TRUE
MT16	10	89720799	TACT	-	PTEN	Frame_Shift_Del	p.T319*	0.64	0.00	190	268	1	Clonal	LOH	FALSE
MT16	13	48941685	A	G	RB1	Missense_Mutation	p.D332G	0.70	0.00	181	225	1	Clonal	LOH	FALSE
MT16	17	12043157	-	T	MAP2K4	Frame_Shift_Ins	p.T349Yfs*4	0.68	0.00	270	247	1	Clonal	LOH	FALSE
MT16	2	25457191	C	G	DNMT3A	Missense_Mutation	p.R899P	0.16	0.00	926	711	0.59	Subclonal	-	FALSE
MT16	22	30069351	G	A	NF2	Missense_Mutation	p.E406K	0.12	0.00	761	754	0.32	Subclonal	-	FALSE
MT16	6	152265419	T	G	ESR1	Missense_Mutation	p.L291R	0.08	0.00	848	535	0.39	Subclonal	-	FALSE
MT72	17	7579448	G	-	TP53	Frame_Shift_Del	p.P60Lfs*43	0.51	0.00	424	585	1	Clonal	LOH	FALSE
MT72	5	67591147	CTTGATGTAAGTATTTGAAATGGAATCCTATACATGAATAATTGGTGATTGCTACAATTCAGGATGAGTTAATGCGTTCTCTTTTCAAAACTGTTTTTCAGGTGGTTGACTCAAAAGGTGTTCGGCAAAAGAAGTTGAACGAGTGGTTGGG	-	PIK3R1	Splice_Site	p.X581_splice	0.26	0.00	445	465	0.98	Clonal	-	FALSE
MT59	5	1295228	G	A	TERT	Promoter		0.39	0.00	139	103	1	Clonal	-	TRUE
MT59	3	178952138	-	T	PIK3CA	Frame_Shift_Ins	p.H1065Lfs*8	0.20	0.00	397	283	1	Clonal	-	FALSE
MT62	17	7574003	G	A	TP53	Nonsense_Mutation	p.R342*	0.47	0.00	713	651	1	Clonal	LOH	FALSE
MT62	12	18691234	C	A	PIK3C2G	Missense_Mutation	p.N1115K	0.14	0.00	348	328	0.53	Subclonal	-	FALSE
MT62	16	89849440	G	C	FANCA	Missense_Mutation	p.A514G	0.29	0.00	1104	486	1	Clonal	-	FALSE
MT62	6	94120846	G	C	EPHA7	Missense_Mutation	p.R69G	0.12	0.00	579	508	0.46	Subclonal	-	FALSE
MT62	6	137540458	G	A	IFNGR1	Missense_Mutation	p.L3F	0.30	0.01	763	291	1	Clonal	-	FALSE
MT62	7	151945055	T	A	KMT2C	Nonsense_Mutation	p.K822*	0.06	0.00	425	566	0.17	Subclonal	LOH	FALSE
MT82	17	7577538	C	T	TP53	Missense_Mutation	p.R248Q	0.63	0.00	509	867	1	Clonal	LOH	TRUE
MT82	3	178916924	CAG	-	PIK3CA	In_Frame_Del	p.P104_V105delinsL	0.37	0.00	630	744	1	Clonal	-	FALSE
MT82	12	46244338	G	A	ARID2	Missense_Mutation	p.C811Y	0.62	0.00	402	609	1	Clonal	LOH	FALSE
MT82	20	39726757	A	G	TOP1	Missense_Mutation	p.H346R	0.13	0.00	643	749	0.7	Subclonal	-	FALSE
MT82	22	41513447	G	C	EP300	Missense_Mutation	p.L117F	0.39	0.00	740	760	1	Clonal	-	FALSE
MT20	16	3788651	C	A	CREBBP	Missense_Mutation	p.D1435Y	0.45	0.00	402	705	1	Clonal	LOH	FALSE
MT20	2	190738308	C	T	PMS1	Missense_Mutation	p.L854F	0.07	0.00	695	652	0.33	Subclonal	-	FALSE
MT20	9	139391330	-	CCCA	NOTCH1	Frame_Shift_Ins	p.S2288Gfs*67	0.68	0.00	560	999	1	Clonal	LOH	FALSE
MT20	23	44942703	A	G	KDM6A	Splice_Site	p.X1095_splice	0.44	0.00	299	578	1	Clonal	LOH	FALSE
MT70	17	7579406	G	C	TP53	Nonsense_Mutation	p.S94*	0.75	0.00	849	809	1	Clonal	LOH	FALSE
MT70	10	89720676	AT	-	PTEN	Frame_Shift_Del	p.T277Ifs*20	0.73	0.00	221	263	1	Clonal	LOH	FALSE
MT70	2	202141621	T	A	CASP8	Missense_Mutation	p.F303L	0.70	0.00	533	491	1	Clonal	LOH	FALSE
MT35	5	1295228	G	A	TERT	Promoter		0.38	0.00	47	609	1	Clonal	-	TRUE
MT35	3	178952085	A	G	PIK3CA	Missense_Mutation	p.H1047R	0.43	0.00	96	445	1	Clonal	-	TRUE
MT35	11	533874	T	C	HRAS	Missense_Mutation	p.Q61R	0.60	0.00	115	851	1	Clonal	LOH	TRUE
MT35	7	140453155	C	T	BRAF	Missense_Mutation	p.D594N	0.05	0.00	146	487	0.27	Subclonal	-	TRUE
MT35	17	7578413	C	A	TP53	Missense_Mutation	p.V173L	0.10	0.00	116	748	0.4	Subclonal	-	TRUE
MT35	3	178936082	G	C	PIK3CA	Missense_Mutation	p.E542Q	0.49	0.00	107	609	1	Clonal	-	TRUE
MT35	17	7578237	CT	-	TP53	Frame_Shift_Del	p.E204Vfs*4	0.25	0.00	206	773	0.95	Clonal	-	FALSE
MT60	4	153249358	A	C	FBXW7	Splice_Site	p.X473_splice	0.20	0.00	295	290	0.9	Clonal	-	FALSE
MT60	5	180045881	C	A	FLT4	Missense_Mutation	p.V964L	0.10	0.00	100	347	0.36	Subclonal	-	FALSE
MT60	23	47038526	C	G	RBM10	Missense_Mutation	p.R153G	0.14	0.00	205	439	0.49	Subclonal	LOH	FALSE
MT60	17	7577587	T	-	TP53	Frame_Shift_Del	p.I232Sfs*15	0.16	0.00	269	252	0.87	Clonal	-	TRUE
MT182	17	7578406	C	T	TP53	Missense_Mutation	p.R175H	0.46	0.00	198	428	0.98	Clonal	LOH	TRUE
MP1	1	32264146	G	C	SPOCD1	Missense_Mutation	p.A642G	0.13	0.00	608	148	0.66	Subclonal	-	FALSE
MP1	1	43296171	C	G	ERMAP	Missense_Mutation	p.L18V	0.19	0.00	825	329	0.73	Subclonal	-	FALSE
MP1	1	44433319	G	C	IPO13	Missense_Mutation	p.G953R	0.40	0.00	762	232	1	Clonal	-	FALSE
MP1	1	55118706	A	C	MROH7	Missense_Mutation	p.Q36P	0.37	0.00	407	153	1	Clonal	LOH	FALSE
MP1	1	160518056	A	G	CD84	Missense_Mutation	p.I343T	0.15	0.00	764	105	1	Clonal	LOH	FALSE
MP1	1	161821590	TTGTCAGCCCC	T	ATF6	Frame_Shift_Del	p.C467*	0.07	0.00	529	95	0.4	Subclonal	-	FALSE
MP1	1	169494136	C	T	F5	Missense_Mutation	p.M1909I	0.12	0.00	256	48	0.57	Subclonal	LOH	FALSE
MP1	1	169775167	C	G	C1orf112	Missense_Mutation	p.I167M	0.03	0.00	541	118	0.16	Subclonal	LOH	FALSE
MP1	1	182569624	C	T	RGS16	Missense_Mutation	p.E138K	0.43	0.00	281	60	1	Clonal	-	FALSE
MP1	1	186114630	C	A	HMCN1	Missense_Mutation	p.P4788T	0.06	0.01	507	110	0.21	Subclonal	-	FALSE
MP1	1	240656578	C	G	GREM2	Missense_Mutation	p.K66N	0.16	0.00	805	145	0.91	Clonal	LOH	FALSE
MP1	10	23244791	G	T	ARMC3	Missense_Mutation	p.K74N	0.04	0.00	399	75	0.16	Subclonal	-	FALSE
MP1	10	72297621	T	G	PALD1	Missense_Mutation	p.Y428D	0.21	0.00	154	39	0.83	Clonal	-	FALSE
MP1	10	90681076	G	T	STAMBPL1	Missense_Mutation	p.D360Y	0.11	0.00	760	446	0.33	Subclonal	-	FALSE
MP1	10	93389818	C	A	PPP1R3C	Missense_Mutation	p.D274Y	0.28	0.00	662	227	1	Clonal	-	FALSE
MP1	10	97607413	C	T	ENTPD1	Nonsense_Mutation	p.Q354*	0.23	0.00	572	186	0.88	Subclonal	-	FALSE
MP1	10	124758086	A	T	IKZF5	Missense_Mutation	p.L19Q	0.26	0.00	671	207	1	Clonal	-	FALSE
MP1	11	376778	G	T	B4GALNT4	Missense_Mutation	p.G552V	0.30	0.00	60	10	0.86	Clonal	-	FALSE
MP1	11	1018390	C	T	MUC6	Missense_Mutation	p.A1471T	0.10	0.00	73	36	0.28	Subclonal	-	FALSE
MP1	11	9202472	A	C	DENND5A	Missense_Mutation	p.S433A	0.24	0.00	538	138	0.69	Subclonal	-	FALSE
MP1	11	95075046	G	C	MAML2	Missense_Mutation	p.A5G	0.37	0.00	602	201	1	Clonal	LOH	FALSE
MP1	12	53668684	G	C	ESPL1	Missense_Mutation	p.W530C	0.26	0.00	378	83	0.74	Subclonal	-	FALSE
MP1	12	112613580	T	C	HECTD4	Missense_Mutation	p.R3706G	0.24	0.00	312	101	0.94	Clonal	-	FALSE
MP1	13	32915166	CAG	C	BRCA2	Frame_Shift_Del	p.E2226Sfs*6	0.19	0.00	507	150	0.74	Subclonal	-	FALSE
MP1	14	24724433	C	G	TGM1	Missense_Mutation	p.E558Q	0.29	0.00	551	204	0.84	Subclonal	-	FALSE
MP1	14	24798295	C	A	ADCY4	Missense_Mutation	p.S499I	0.24	0.00	460	147	0.69	Subclonal	-	FALSE
MP1	14	78374228	A	G	ADCK1	Missense_Mutation	p.D275G	0.35	0.00	325	85	1	Clonal	LOH	FALSE
MP1	14	89041164	G	T	ZC3H14	Missense_Mutation	p.S330I	0.37	0.00	366	134	1	Clonal	LOH	FALSE
MP1	14	100758831	C	A	SLC25A29	Missense_Mutation	p.C234F	0.36	0.00	396	165	1	Clonal	LOH	FALSE
MP1	15	31294755	G	C	TRPM1	Nonsense_Mutation	p.S1400*	0.05	0.00	635	192	0.15	Subclonal	-	FALSE
MP1	15	40703840	CTGGCTGCTG	C	IVD	In_Frame_Del	p.A217_V219del	0.13	0.00	463	143	0.52	Subclonal	-	FALSE
MP1	15	43122276	C	A	TTBK2	Missense_Mutation	p.G96C	0.09	0.00	182	47	0.25	Subclonal	-	FALSE
MP1	15	73562686	G	C	NEO1	Missense_Mutation	p.L916F	0.20	0.00	434	172	0.58	Subclonal	-	FALSE
MP1	15	74494504	C	G	STRA6	Missense_Mutation	p.Q74H	0.29	0.01	292	116	0.83	Subclonal	-	FALSE
MP1	15	74573131	C	T	CCDC33	Missense_Mutation	p.L338F	0.24	0.00	180	52	0.69	Subclonal	-	FALSE
MP1	15	83328653	T	G	AP3B2	Missense_Mutation	p.K1014N	0.27	0.00	403	126	1	Clonal	-	FALSE
MP1	16	1270560	G	C	CACNA1H	Missense_Mutation	p.E2210Q	0.27	0.00	173	87	0.78	Subclonal	-	FALSE
MP1	16	3613383	C	T	NLRC3	Missense_Mutation	p.G566S	0.22	0.00	334	66	0.83	Subclonal	-	FALSE
MP1	16	66421000	G	T	CDH5	Missense_Mutation	p.G167W	0.25	0.00	187	71	0.72	Subclonal	-	FALSE
MP1	16	84034351	G	T	NECAB2	Missense_Mutation	p.D329Y	0.04	0.00	285	75	0.16	Subclonal	-	FALSE
MP1	16	88600914	G	C	ZFPM1	Missense_Mutation	p.G850R	0.29	0.02	174	50	1	Clonal	-	FALSE
MP1	17	650608	A	C	GEMIN4	Missense_Mutation	p.F159V	0.37	0.00	664	323	NA	Subclonal	LOH	FALSE
MP1	17	7579358	C	G	TP53	Missense_Mutation	p.R110P	0.32	0.00	340	126	0.92	Clonal	LOH	TRUE
MP1	17	39723554	C	T	KRT9	Missense_Mutation	p.G615R	0.27	0.01	478	115	1	Clonal	-	FALSE
MP1	17	40947744	C	G	WNK4	Missense_Mutation	p.P1042A	0.03	0.00	735	259	0.07	Subclonal	-	FALSE
MP1	17	46019105	C	G	PNPO	Missense_Mutation	p.L22V	0.29	0.00	780	186	1	Clonal	-	FALSE
MP1	17	72613528	C	G	CD300E	Missense_Mutation	p.E39D	0.12	0.00	763	142	0.7	Subclonal	-	FALSE
MP1	17	76046211	T	A	TNRC6C	Missense_Mutation	p.N356K	0.19	0.01	680	185	0.91	Clonal	-	FALSE
MP1	18	21752398	G	GAT	OSBPL1A	Frame_Shift_Ins	p.I716Sfs*17	0.21	0.00	442	106	0.62	Subclonal	-	FALSE
MP1	18	47810374	G	C	CXXC1	Missense_Mutation	p.R439G	0.25	0.00	780	201	0.98	Clonal	-	FALSE
MP1	19	6832129	G	C	VAV1	Missense_Mutation	p.D476H	0.14	0.02	287	50	0.7	Subclonal	-	FALSE
MP1	19	8368811	C	A	CD320	Missense_Mutation	p.D144Y	0.23	0.00	663	118	0.88	Subclonal	-	FALSE
MP1	19	12089901	G	A	ZNF763	Missense_Mutation	p.A391T	0.42	0.00	416	99	1	Clonal	-	FALSE
MP1	19	15271721	TGGGAACCCGCAGGAAGC	T	NOTCH3	Frame_Shift_Del	p.R2234Qfs*2	0.23	0.00	447	90	1	Clonal	-	FALSE
MP1	19	16643848	C	T	CHERP	Missense_Mutation	p.D166N	0.20	0.00	754	142	0.99	Clonal	-	FALSE
MP1	19	19338879	G	T	NCAN	Missense_Mutation	p.W817L	0.21	0.00	454	118	1	Clonal	-	FALSE
MP1	19	36135647	G	C	ETV2	Missense_Mutation	p.V308L	0.26	0.00	479	123	1	Clonal	-	FALSE
MP1	19	44117294	C	G	SRRM5	Missense_Mutation	p.Q341E	0.30	0.00	579	186	1	Clonal	-	FALSE
MP1	19	48898714	G	C	C19orf68	Missense_Mutation	p.E465Q	0.08	0.00	89	15	0.3	Subclonal	-	FALSE
MP1	19	51135686	C	G	SYT3	Missense_Mutation	p.E191Q	0.25	0.00	481	105	0.97	Clonal	-	FALSE
MP1	19	55814734	C	G	BRSK1	Missense_Mutation	p.P362A	0.19	0.00	375	152	0.74	Subclonal	-	FALSE
MP1	2	74719523	G	C	TTC31	Missense_Mutation	p.G371A	0.29	0.00	771	195	1	Clonal	-	FALSE
MP1	2	84806703	TC	T	DNAH6	Frame_Shift_Del	p.F711Lfs*43	0.25	0.00	472	115	0.95	Clonal	-	FALSE
MP1	2	179426285	C	T	TTN	Missense_Mutation	p.E28192K	0.23	0.00	804	245	0.88	Subclonal	-	FALSE
MP1	2	179467102	T	A	TTN	Missense_Mutation	p.R18343W	0.28	0.00	771	233	1	Clonal	-	FALSE
MP1	2	222298999	G	T	EPHA4	Missense_Mutation	p.P787T	0.25	0.00	191	78	0.97	Clonal	-	FALSE
MP1	20	76645	G	A	DEFB125	Splice_Site	p.X20_splice	0.29	0.00	254	70	1	Clonal	-	FALSE
MP1	20	37356198	G	T	SLC32A1	Missense_Mutation	p.K164N	0.25	0.00	740	186	0.97	Clonal	-	FALSE
MP1	20	39316597	G	C	MAFB	Missense_Mutation	p.C298W	0.23	0.00	420	173	0.88	Clonal	-	FALSE
MP1	20	49508043	C	T	ADNP	Missense_Mutation	p.D1070N	0.27	0.00	775	249	0.78	Subclonal	-	FALSE
MP1	20	49508589	C	G	ADNP	Missense_Mutation	p.E888Q	0.26	0.00	801	257	0.75	Subclonal	-	FALSE
MP1	20	49508863	C	T	ADNP	Nonsense_Mutation	p.W796*	0.26	0.00	615	242	0.74	Subclonal	-	FALSE
MP1	20	49508892	C	T	ADNP	Missense_Mutation	p.E787K	0.25	0.00	575	225	0.72	Subclonal	-	FALSE
MP1	20	49508898	C	T	ADNP	Missense_Mutation	p.E785K	0.25	0.00	565	226	0.73	Subclonal	-	FALSE
MP1	20	49509012	C	T	ADNP	Missense_Mutation	p.E747K	0.30	0.00	663	250	0.85	Subclonal	-	FALSE
MP1	21	27102114	T	C	ATP5J	Splice_Site	p.X6_splice	0.27	0.00	64	19	0.76	Subclonal	-	FALSE
MP1	21	46876042	AGGCCCTGGGCACCACTCACGG	A	COL18A1	In_Frame_Del	p.W202_P208del	0.09	0.00	637	259	0.35	Subclonal	-	FALSE
MP1	22	23610617	G	A	BCR	Missense_Mutation	p.R592Q	0.28	0.00	192	70	1	Clonal	-	FALSE
MP1	22	29130432	C	G	CHEK2	Missense_Mutation	p.W93S	0.26	0.00	562	164	0.75	Subclonal	-	FALSE
MP1	22	33402577	T	C	SYN3	Missense_Mutation	p.D24G	0.23	0.00	329	91	0.91	Clonal	-	FALSE
MP1	3	11372820	A	G	ATG7	Missense_Mutation	p.I229V	0.28	0.00	368	151	0.7	Subclonal	-	FALSE
MP1	3	98531285	A	T	DCBLD2	Missense_Mutation	p.N418K	0.07	0.00	440	130	0.26	Subclonal	-	FALSE
MP1	3	108818312	C	T	MORC1	Missense_Mutation	p.G106R	0.13	0.00	192	43	0.73	Subclonal	-	FALSE
MP1	3	147113934	C	A	ZIC4	Missense_Mutation	p.K181N	0.20	0.00	882	102	1	Clonal	LOH	FALSE
MP1	3	148545800	T	G	CPB1	Missense_Mutation	p.F28C	0.14	0.01	802	139	0.83	Subclonal	-	FALSE
MP1	3	154860084	C	T	MME	Nonsense_Mutation	p.R365*	0.12	0.00	699	108	0.71	Subclonal	-	FALSE
MP1	3	157841669	C	A	RSRC1	Nonsense_Mutation	p.S70*	0.51	0.00	763	129	1	Clonal	-	FALSE
MP1	4	1962873	G	C	WHSC1	Missense_Mutation	p.D1129H	0.38	0.00	277	128	1	Clonal	LOH	FALSE
MP1	4	8995450	C	T	KIAA0232	Missense_Mutation	p.S1114F	0.34	0.00	422	217	0.97	Clonal	LOH	FALSE
MP1	4	100574955	G	A	RP11-766F14.2	Missense_Mutation	p.S284F	0.12	0.00	722	205	0.47	Subclonal	-	FALSE
MP1	4	111397773	C	G	ENPEP	Nonsense_Mutation	p.S68*	0.25	0.00	447	125	0.96	Clonal	-	FALSE
MP1	5	67592135	AGCAGTAAACAGGGCTGCTAT	A	PIK3R1	Frame_Shift_Del	p.S652Lfs*28	0.08	0.00	519	191	0.23	Subclonal	-	FALSE
MP1	5	95728749	G	C	PCSK1	Missense_Mutation	p.R740G	0.28	0.00	645	154	0.94	Clonal	-	FALSE
MP1	5	149227030	C	T	PPARGC1B	Missense_Mutation	p.S994L	0.27	0.00	778	220	1	Clonal	-	FALSE
MP1	5	173376969	C	T	CPEB4	Missense_Mutation	p.A518V	0.38	0.00	286	96	1	Clonal	LOH	FALSE
MP1	6	31122293	C	G	CCHCR1	Missense_Mutation	p.Q261E	0.24	0.01	833	511	0.7	Subclonal	-	FALSE
MP1	6	43748554	C	G	VEGFA	Missense_Mutation	p.R170G	0.25	0.00	236	68	0.71	Subclonal	-	FALSE
MP1	6	44081938	C	T	MRPL14	Missense_Mutation	p.R37Q	0.27	0.00	667	228	0.78	Subclonal	-	FALSE
MP1	6	79679568	G	C	PHIP	Nonsense_Mutation	p.Y1063*	0.22	0.00	65	36	0.83	Clonal	-	FALSE
MP1	6	87725456	C	T	HTR1E	Missense_Mutation	p.T135M	0.27	0.00	672	120	1	Clonal	-	FALSE
MP1	6	130363937	T	A	L3MBTL3	Missense_Mutation	p.S31R	0.22	0.00	722	228	0.86	Subclonal	-	FALSE
MP1	6	136599186	C	T	BCLAF1	Missense_Mutation	p.G278E	0.13	0.00	389	109	0.52	Subclonal	-	FALSE
MP1	6	169620369	GTAGGTTTGGTCA	G	THBS2	In_Frame_Del	p.D1142_Y1145del	0.16	0.00	252	80	0.47	Subclonal	-	FALSE
MP1	7	6656890	G	T	ZNF853	Nonsense_Mutation	p.E28*	0.23	0.00	314	105	0.88	Clonal	-	FALSE
MP1	7	100815599	C	A	NAT16	Missense_Mutation	p.D291Y	0.05	0.00	295	67	0.18	Subclonal	-	FALSE
MP1	7	138555958	ACGGGAGGTGC	A	KIAA1549	Frame_Shift_Del	p.A1496Sfs*52	0.25	0.00	669	116	1	Clonal	-	FALSE
MP1	7	141673468	G	A	TAS2R38	Missense_Mutation	p.R8C	0.34	0.00	516	158	1	Clonal	-	FALSE
MP1	8	21977644	C	T	HR	Missense_Mutation	p.R940Q	0.35	0.00	742	216	1	Clonal	-	FALSE
MP1	8	97797435	C	A	CPQ	Missense_Mutation	p.H104N	0.27	0.00	519	135	1	Clonal	-	FALSE
MP1	8	144895492	G	T	SCRIB	Missense_Mutation	p.L186M	0.28	0.01	262	97	1	Clonal	-	FALSE
MP1	9	463545	C	T	DOCK8	Missense_Mutation	p.R2033C	0.18	0.00	544	123	0.69	Subclonal	-	FALSE
MP1	9	37541330	C	A	FBXO10	Nonsense_Mutation	p.E146*	0.24	0.00	765	230	0.94	Clonal	-	FALSE
MP1	9	95099859	G	A	CENPP	Missense_Mutation	p.G109E	0.23	0.00	351	118	0.65	Subclonal	-	FALSE
MP1	9	130479996	T	C	TTC16	Missense_Mutation	p.L124S	0.39	0.00	317	127	1	Clonal	LOH	FALSE
MP1	23	13681520	G	C	TCEANC	Missense_Mutation	p.R328T	0.34	0.00	609	246	0.98	Clonal	-	FALSE
MP1	23	44922693	AC	A	KDM6A	Frame_Shift_Del	p.R519Dfs*29	0.21	0.00	1102	319	0.8	Subclonal	-	FALSE
MP1	23	47308469	C	A	ZNF41	Nonsense_Mutation	p.E234*	0.24	0.01	517	102	1	Clonal	-	FALSE
MP1	23	49645301	C	T	USP27X	Missense_Mutation	p.R131W	0.13	0.01	839	323	0.62	Subclonal	-	FALSE
MP1	23	70342191	C	A	MED12	Missense_Mutation	p.Q415K	0.03	0.00	626	217	0.12	Subclonal	-	FALSE
MP1	23	102979223	G	A	GLRA4	Missense_Mutation	p.R93W	0.08	0.00	156	50	0.3	Subclonal	-	FALSE
MP1	23	107979101	C	G	IRS4	Missense_Mutation	p.Q158H	0.15	0.01	499	102	0.6	Subclonal	-	FALSE
MP1	23	125298898	C	T	DCAF12L2	Missense_Mutation	p.R337H	0.32	0.00	699	134	1	Clonal	-	FALSE
MP1	23	152686791	C	G	ZFP92	Missense_Mutation	p.P319R	0.19	0.00	458	134	0.73	Subclonal	-	FALSE
MP1	23	153674848	G	C	FAM50A	Missense_Mutation	p.E128Q	0.49	0.00	99	36	1	Clonal	-	FALSE
MP1	23	154721241	T	A	TMLHE	Missense_Mutation	p.R407S	0.17	0.00	47	20	0.66	Subclonal	-	FALSE
MP11	1	3407502	TCGGGGAG	T	MEGF6	Frame_Shift_Del	p.L1511Rfs*5	0.19	0.00	532	138	0.78	Subclonal	-	FALSE
MP11	1	3683813	A	G	CCDC27	Missense_Mutation	p.E516G	0.03	0.00	634	110	0.14	Subclonal	-	FALSE
MP11	1	7848188	G	C	PER3	Missense_Mutation	p.K158N	0.11	0.00	800	220	0.56	Subclonal	-	FALSE
MP11	1	7880537	A	T	PER3	Missense_Mutation	p.Q590H	0.37	0.00	806	140	1	Clonal	-	FALSE
MP11	1	8925413	C	A	ENO1	Missense_Mutation	p.D266Y	0.09	0.00	742	87	0.45	Subclonal	-	FALSE
MP11	1	16056383	C	G	PLEKHM2	Missense_Mutation	p.L723V	0.04	0.00	341	64	0.18	Subclonal	-	FALSE
MP11	1	23519740	G	A	HTR1D	Missense_Mutation	p.P325S	0.24	0.00	825	146	0.95	Subclonal	-	FALSE
MP11	1	27216371	G	C	GPN2	Missense_Mutation	p.L73V	0.02	0.00	776	194	0.1	Subclonal	-	FALSE
MP11	1	35454216	G	C	ZMYM6	Missense_Mutation	p.L829V	0.15	0.00	505	112	0.62	Subclonal	-	FALSE
MP11	1	36024790	A	C	NCDN	Missense_Mutation	p.Y39S	0.05	0.00	518	88	0.21	Subclonal	-	FALSE
MP11	1	43774759	G	A	TIE1	Missense_Mutation	p.R382Q	0.20	0.00	438	88	0.98	Clonal	-	FALSE
MP11	1	45190981	G	C	C1orf228	Missense_Mutation	p.S410T	0.23	0.00	778	207	1	Clonal	-	FALSE
MP11	1	47025921	G	A	MKNK1	Missense_Mutation	p.P386L	0.21	0.00	449	83	1	Clonal	-	FALSE
MP11	1	53925217	C	T	DMRTB1	Missense_Mutation	p.R31C	0.23	0.00	627	124	1	Clonal	-	FALSE
MP11	1	55282720	G	A	C1orf177	Missense_Mutation	p.R370Q	0.09	0.00	674	100	0.45	Subclonal	-	FALSE
MP11	1	55529225	C	A	PCSK9	Missense_Mutation	p.H683N	0.06	0.00	341	76	0.32	Subclonal	-	FALSE
MP11	1	110923652	G	C	SLC16A4	Missense_Mutation	p.L160V	0.03	0.00	600	132	0.13	Subclonal	LOH	FALSE
MP11	1	150444924	A	G	RPRD2	Missense_Mutation	p.Y1167C	0.04	0.00	833	216	0.19	Subclonal	-	FALSE
MP11	1	154744829	G	C	KCNN3	Missense_Mutation	p.A357G	0.05	0.00	489	74	0.24	Subclonal	-	FALSE
MP11	1	158587344	G	C	SPTA1	Missense_Mutation	p.Q2195E	0.04	0.00	523	73	0.22	Subclonal	-	FALSE
MP11	1	159907539	G	T	IGSF9	Missense_Mutation	p.L113M	0.06	0.00	693	115	0.39	Subclonal	-	FALSE
MP11	1	160146034	A	G	ATP1A4	Missense_Mutation	p.M822V	0.05	0.00	774	181	0.28	Subclonal	-	FALSE
MP11	1	161137785	G	T	PPOX	Missense_Mutation	p.R113S	0.14	0.00	376	56	0.86	Subclonal	-	FALSE
MP11	1	167814860	C	T	ADCY10	Missense_Mutation	p.R983Q	0.04	0.00	745	135	0.18	Subclonal	-	FALSE
MP11	1	179079589	C	G	ABL2	Missense_Mutation	p.E551D	0.04	0.00	716	117	0.22	Subclonal	-	FALSE
MP11	1	181701861	A	G	CACNA1E	Missense_Mutation	p.E880G	0.05	0.00	752	188	0.25	Subclonal	-	FALSE
MP11	1	209964083	GCTCCAGGCTGACGGGACCAAAGAGCTC	G	IRF6	In_Frame_Del	p.E264_E272del	0.11	0.01	975	145	0.58	Subclonal	-	FALSE
MP11	1	210977394	T	C	KCNH1	Missense_Mutation	p.Y526C	0.05	0.00	693	110	0.24	Subclonal	-	FALSE
MP11	1	216371837	C	G	USH2A	Missense_Mutation	p.G1301R	0.06	0.00	492	48	0.3	Subclonal	-	FALSE
MP11	1	226027005	G	C	EPHX1	Missense_Mutation	p.W227S	0.06	0.00	423	69	0.3	Subclonal	-	FALSE
MP11	1	228335367	C	T	GUK1	Missense_Mutation	p.R169W	0.28	0.00	250	76	1	Subclonal	-	FALSE
MP11	1	228560418	C	G	OBSCN	Nonsense_Mutation	p.Y8270*	0.07	0.00	645	183	0.33	Subclonal	-	FALSE
MP11	1	240341278	T	C	FMN2	Missense_Mutation	p.S614P	0.03	0.00	752	68	0.23	Subclonal	-	FALSE
MP11	1	240370244	T	C	FMN2	Missense_Mutation	p.L711P	0.09	0.00	853	152	0.72	Subclonal	-	FALSE
MP11	1	247875276	C	A	OR6F1	Missense_Mutation	p.R261L	0.03	0.00	872	139	0.23	Subclonal	-	FALSE
MP11	10	48413690	C	G	GDF2	Missense_Mutation	p.C393S	0.31	0.00	688	124	1	Clonal	-	FALSE
MP11	10	92672721	G	A	ANKRD1	Missense_Mutation	p.P288S	0.15	0.00	818	139	0.76	Subclonal	-	FALSE
MP11	10	124266258	G	C	HTRA1	Missense_Mutation	p.E277Q	0.09	0.00	852	247	0.52	Subclonal	-	FALSE
MP11	10	127350546	A	T	TEX36	Missense_Mutation	p.F18I	0.06	0.01	493	101	0.24	Subclonal	-	FALSE
MP11	11	2906301	G	T	CDKN1C	Missense_Mutation	p.P140Q	0.07	0.00	81	25	0.3	Subclonal	-	FALSE
MP11	11	10649537	G	A	MRVI1	Missense_Mutation	p.P293S	0.23	0.00	240	52	0.93	Subclonal	-	FALSE
MP11	11	12315462	A	G	MICALCL	Missense_Mutation	p.R162G	0.03	0.00	717	147	0.14	Subclonal	-	FALSE
MP11	11	17580708	A	C	OTOG	Missense_Mutation	p.T417P	0.05	0.00	314	62	0.24	Subclonal	-	FALSE
MP11	11	18111772	T	C	SAAL1	Missense_Mutation	p.H180R	0.03	0.00	608	107	0.16	Subclonal	-	FALSE
MP11	11	59378052	C	G	OSBP	Missense_Mutation	p.E125Q	0.05	0.00	807	156	0.23	Subclonal	-	FALSE
MP11	11	65363090	C	G	KCNK7	Missense_Mutation	p.A52P	0.05	0.00	309	77	0.19	Subclonal	-	FALSE
MP11	11	67817972	G	A	TCIRG1	Nonsense_Mutation	p.W752*	0.05	0.00	177	42	0.18	Subclonal	-	FALSE
MP11	11	71940252	A	G	INPPL1	Missense_Mutation	p.T213A	0.03	0.00	727	153	0.17	Subclonal	-	FALSE
MP11	11	72013356	G	A	CLPB	Nonsense_Mutation	p.R417*	0.17	0.00	596	108	0.83	Subclonal	-	FALSE
MP11	11	72292424	G	C	PDE2A	Missense_Mutation	p.N674K	0.03	0.00	230	40	0.17	Subclonal	-	FALSE
MP11	11	76919498	C	A	MYO7A	Missense_Mutation	p.D1960E	0.05	0.00	548	129	0.23	Subclonal	-	FALSE
MP11	11	78423611	T	C	TENM4	Missense_Mutation	p.T1324A	0.05	0.00	776	99	0.23	Subclonal	-	FALSE
MP11	11	85742652	G	C	PICALM	Nonsense_Mutation	p.Y44*	0.21	0.00	418	46	1	Clonal	-	FALSE
MP11	11	92534346	A	G	FAT3	Missense_Mutation	p.I2723V	0.06	0.00	812	171	0.31	Subclonal	-	FALSE
MP11	11	106880788	T	G	GUCY1A2	Missense_Mutation	p.Q541H	0.05	0.00	381	51	0.25	Subclonal	-	FALSE
MP11	11	111745880	C	G	FDXACB1	Missense_Mutation	p.K547N	0.23	0.00	826	178	0.91	Subclonal	-	FALSE
MP11	11	117651500	G	C	DSCAML1	Missense_Mutation	p.S84R	0.03	0.00	391	61	0.11	Subclonal	-	FALSE
MP11	11	118455271	T	G	ARCN1	Missense_Mutation	p.S244A	0.17	0.00	426	91	0.69	Subclonal	-	FALSE
MP11	11	119059793	G	A	PDZD3	Missense_Mutation	p.G456E	0.14	0.00	768	225	0.58	Subclonal	-	FALSE
MP11	12	2932953	G	C	ITFG2	Missense_Mutation	p.E362Q	0.08	0.00	346	48	0.3	Subclonal	LOH	FALSE
MP11	12	8610511	T	A	CLEC6A	Missense_Mutation	p.S17T	0.03	0.00	304	60	0.16	Subclonal	-	FALSE
MP11	12	32137980	TGAAACTCAAATCA	T	KIAA1551	Frame_Shift_Del	p.K1365Lfs*9	0.13	0.01	635	115	0.64	Subclonal	-	FALSE
MP11	12	48374750	C	G	COL2A1	Missense_Mutation	p.G822A	0.10	0.00	155	35	0.48	Subclonal	-	FALSE
MP11	12	57317625	T	A	SDR9C7	Missense_Mutation	p.S312C	0.05	0.00	209	25	0.19	Subclonal	LOH	FALSE
MP11	12	71977815	A	C	LGR5	Missense_Mutation	p.K675N	0.05	0.00	781	84	0.26	Subclonal	-	FALSE
MP11	12	91497974	G	C	LUM	Missense_Mutation	p.L329V	0.07	0.00	501	94	0.28	Subclonal	LOH	FALSE
MP11	12	93170651	C	G	EEA1	Missense_Mutation	p.C1361S	0.06	0.00	754	156	0.26	Subclonal	LOH	FALSE
MP11	12	117513105	CTTAAATCTCCGATGGAG	C	TESC	Frame_Shift_Del	p.L28Afs*48	0.29	0.00	265	59	1	Subclonal	-	FALSE
MP11	12	130184788	C	A	TMEM132D	Missense_Mutation	p.V179L	0.07	0.00	793	111	0.28	Subclonal	LOH	FALSE
MP11	13	21212630	T	A	IFT88	Missense_Mutation	p.S525T	0.03	0.00	432	97	0.17	Subclonal	-	FALSE
MP11	13	23906391	C	T	SACS	Missense_Mutation	p.R3875H	0.05	0.00	775	172	0.27	Subclonal	-	FALSE
MP11	13	53617304	T	G	OLFM4	Missense_Mutation	p.I212S	0.04	0.00	615	62	0.24	Subclonal	-	FALSE
MP11	13	88328086	C	G	SLITRK5	Missense_Mutation	p.T148S	0.07	0.00	823	113	0.4	Subclonal	-	FALSE
MP11	14	20665640	G	C	OR11G2	Missense_Mutation	p.G49A	0.28	0.00	640	104	1	Clonal	-	FALSE
MP11	14	20824766	G	C	PARP2	Missense_Mutation	p.G429A	0.05	0.00	548	109	0.25	Subclonal	-	FALSE
MP11	14	23342591	C	G	LRP10	Missense_Mutation	p.R51G	0.26	0.00	812	190	1	Clonal	-	FALSE
MP11	14	45605733	G	A	FANCM	Missense_Mutation	p.E167K	0.29	0.00	770	112	1	Clonal	-	FALSE
MP11	14	45715966	G	C	MIS18BP1	Nonsense_Mutation	p.S175*	0.05	0.00	275	42	0.22	Subclonal	-	FALSE
MP11	15	25924530	G	T	ATP10A	Missense_Mutation	p.D1486E	0.16	0.00	657	122	0.82	Subclonal	-	FALSE
MP11	15	40660547	C	A	DISP2	Missense_Mutation	p.P745H	0.23	0.00	318	86	0.92	Subclonal	LOH	FALSE
MP11	15	59185539	A	C	SLTM	Missense_Mutation	p.V558G	0.31	0.00	718	158	1	Clonal	LOH	FALSE
MP11	15	65040643	C	C	RBPMS2	Missense_Mutation	p.A181G	0.05	0.00	168	40	0.19	Subclonal	LOH	FALSE
MP11	15	89659671	C	G	ABHD2	Missense_Mutation	p.P38R	0.08	0.00	759	107	0.41	Subclonal	LOH	FALSE
MP11	15	91795651	C	G	SV2B	Missense_Mutation	p.R229G	0.06	0.00	467	81	0.3	Subclonal	LOH	FALSE
MP11	16	360012	G	C	AXIN1	Missense_Mutation	p.S359R	0.18	0.00	257	71	0.73	Subclonal	-	FALSE
MP11	16	2868966	T	G	PRSS21	Missense_Mutation	p.D182E	0.07	0.00	206	69	0.29	Subclonal	-	FALSE
MP11	16	4438629	T	A	CORO7	Missense_Mutation	p.T207S	0.13	0.00	296	92	0.53	Subclonal	-	FALSE
MP11	16	4920545	A	T	UBN1	Missense_Mutation	p.K447M	0.16	0.00	612	131	0.63	Subclonal	-	FALSE
MP11	16	27519974	C	T	GTF3C1	Missense_Mutation	p.E377K	0.25	0.00	211	57	0.99	Subclonal	-	FALSE
MP11	16	29705988	T	A	QPRT	Missense_Mutation	p.L6Q	0.25	0.00	736	154	1	Clonal	-	FALSE
MP11	16	48258332	C	T	ABCC11	Missense_Mutation	p.R135H	0.06	0.00	611	113	0.24	Subclonal	-	FALSE
MP11	16	67877272	A	T	THAP11	Missense_Mutation	p.E272V	0.04	0.00	868	281	0.22	Subclonal	-	FALSE
MP11	16	67985812	G	C	SLC12A4	Missense_Mutation	p.T351R	0.03	0.00	492	77	0.18	Subclonal	-	FALSE
MP11	16	70841743	A	T	HYDIN	Missense_Mutation	p.Y5036N	0.09	0.00	818	138	0.47	Subclonal	-	FALSE
MP11	16	83712002	G	A	CDH13	Missense_Mutation	p.V539M	0.06	0.00	690	111	0.29	Subclonal	-	FALSE
MP11	16	84050848	C	T	SLC38A8	Missense_Mutation	p.V284I	0.13	0.01	594	105	0.66	Subclonal	-	FALSE
MP11	17	7577593	TAC	T	TP53	Frame_Shift_Del	p.C229Yfs*10	0.35	0.00	337	91	1	Clonal	LOH	FALSE
MP11	17	10543699	A	G	MYH3	Missense_Mutation	p.C793R	0.18	0.00	808	222	0.72	Subclonal	-	FALSE
MP11	17	11711198	T	C	DNAH9	Missense_Mutation	p.I2857T	0.05	0.00	191	25	0.21	Subclonal	-	FALSE
MP11	17	30207754	C	G	UTP6	Missense_Mutation	p.D269H	0.04	0.00	469	95	0.15	Subclonal	LOH	FALSE
MP11	17	34105907	G	C	MMP28	Missense_Mutation	p.R122G	0.03	0.01	802	185	0.13	Subclonal	LOH	FALSE
MP11	17	36517616	C	G	SOCS7	Missense_Mutation	p.Q271E	0.07	0.00	717	158	0.27	Subclonal	LOH	FALSE
MP11	17	37369375	C	G	STAC2	Missense_Mutation	p.G335A	0.07	0.00	407	103	0.3	Subclonal	LOH	FALSE
MP11	17	39334052	A	T	KRTAP4-2	Missense_Mutation	p.V122E	0.09	0.00	414	36	0.37	Subclonal	LOH	FALSE
MP11	17	39967115	C	G	LEPREL4	Missense_Mutation	p.D263H	0.04	0.00	342	74	0.18	Subclonal	LOH	FALSE
MP11	17	48456548	G	C	EME1	Missense_Mutation	p.S411T	0.04	0.01	769	151	0.21	Subclonal	LOH	FALSE
MP11	17	60741996	A	G	MRC2	Missense_Mutation	p.N69S	0.04	0.00	847	235	0.19	Subclonal	LOH	FALSE
MP11	17	74735077	T	C	MFSD11	Splice_Site	p.X51_splice	0.09	0.00	780	237	0.38	Subclonal	LOH	FALSE
MP11	17	76128928	C	A	TMC8	Missense_Mutation	p.L170I	0.09	0.00	501	149	0.34	Subclonal	LOH	FALSE
MP11	17	79792506	C	T	PPP1R27	Missense_Mutation	p.V72M	0.09	0.01	672	146	0.35	Subclonal	LOH	FALSE
MP11	18	54483298	C	G	WDR7	Missense_Mutation	p.T1076R	0.06	0.00	297	47	0.24	Subclonal	-	FALSE
MP11	18	76757233	G	T	SALL3	Missense_Mutation	p.V1272F	0.18	0.01	535	120	0.72	Subclonal	-	FALSE
MP11	19	2275921	C	G	C19orf35	Missense_Mutation	p.A394P	0.08	0.00	50	18	0.24	Subclonal	LOH	FALSE
MP11	19	4538458	G	C	LRG1	Missense_Mutation	p.P180A	0.36	0.00	664	200	1	Clonal	LOH	FALSE
MP11	19	11266370	C	G	SPC24	Missense_Mutation	p.R28P	0.16	0.00	523	89	0.8	Subclonal	-	FALSE
MP11	19	14090305	T	A	RFX1	Missense_Mutation	p.Q263L	0.19	0.00	221	63	0.95	Subclonal	-	FALSE
MP11	19	30935681	G	T	ZNF536	Missense_Mutation	p.K404N	0.04	0.00	511	97	0.19	Subclonal	-	FALSE
MP11	19	38385529	G	C	WDR87	Missense_Mutation	p.Q233E	0.15	0.00	550	112	0.75	Subclonal	-	FALSE
MP11	19	39226025	G	C	CAPN12	Missense_Mutation	p.T378S	0.15	0.00	737	157	0.76	Subclonal	-	FALSE
MP11	19	39517598	T	A	FBXO27	Missense_Mutation	p.Q207L	0.07	0.00	653	130	0.34	Subclonal	-	FALSE
MP11	19	46476938	A	C	NOVA2	Translation_Start_Site	p.M1?	0.05	0.00	81	22	0.25	Subclonal	-	FALSE
MP11	19	50119113	G	C	PRR12	Missense_Mutation	p.E1712Q	0.04	0.00	714	131	0.22	Subclonal	-	FALSE
MP11	19	50384650	G	T	TBC1D17	Missense_Mutation	p.G149C	0.10	0.00	470	97	0.48	Subclonal	-	FALSE
MP11	19	58879930	C	T	ZNF837	Missense_Mutation	p.R257Q	0.03	0.01	573	162	0.17	Subclonal	-	FALSE
MP11	2	26951108	C	T	KCNK3	Missense_Mutation	p.S266L	0.11	0.00	419	106	0.56	Subclonal	-	FALSE
MP11	2	27317438	C	G	KHK	Missense_Mutation	p.I101M	0.08	0.00	721	122	0.42	Subclonal	-	FALSE
MP11	2	27600738	G	C	ZNF513	Missense_Mutation	p.H434D	0.10	0.00	794	239	0.5	Subclonal	-	FALSE
MP11	2	27854750	G	C	GPN1	Missense_Mutation	p.A113P	0.04	0.00	767	167	0.21	Subclonal	-	FALSE
MP11	2	47748402	G	C	KCNK12	Missense_Mutation	p.L313V	0.14	0.00	475	70	0.71	Subclonal	-	FALSE
MP11	2	61275747	C	G	PEX13	Missense_Mutation	p.Q352E	0.16	0.00	817	192	0.8	Subclonal	-	FALSE
MP11	2	97474358	G	C	CNNM4	Missense_Mutation	p.R670P	0.03	0.00	527	57	0.16	Subclonal	-	FALSE
MP11	2	155158075	A	C	GALNT13	Missense_Mutation	p.K377Q	0.04	0.00	344	69	0.19	Subclonal	-	FALSE
MP11	2	165561617	T	A	COBLL1	Splice_Site	p.X333_splice	0.03	0.00	337	39	0.15	Subclonal	-	FALSE
MP11	2	179433874	A	G	TTN	Missense_Mutation	p.I25662T	0.04	0.00	804	172	0.22	Subclonal	-	FALSE
MP11	2	202438993	C	T	ALS2CR11	Missense_Mutation	p.C231Y	0.04	0.00	631	89	0.22	Subclonal	-	FALSE
MP11	2	211523398	G	C	CPS1	Missense_Mutation	p.G1254R	0.03	0.00	571	86	0.18	Subclonal	-	FALSE
MP11	2	223423274	G	C	SGPP2	Missense_Mutation	p.G286A	0.16	0.00	845	184	0.95	Subclonal	-	FALSE
MP11	2	234737614	G	A	MROH2A	Missense_Mutation	p.E1448K	0.04	0.00	665	115	0.2	Subclonal	-	FALSE
MP11	2	242757643	C	G	NEU4	Missense_Mutation	p.R255G	0.05	0.00	468	128	0.19	Subclonal	-	FALSE
MP11	20	1961099	C	T	PDYN	Missense_Mutation	p.R212Q	0.11	0.00	789	179	0.44	Subclonal	-	FALSE
MP11	20	3145507	C	G	LZTS3	Missense_Mutation	p.D539H	0.07	0.00	260	80	0.29	Subclonal	-	FALSE
MP11	20	3145509	C	G	LZTS3	Missense_Mutation	p.S538T	0.07	0.00	260	82	0.29	Subclonal	-	FALSE
MP11	20	10600935	C	G	SLX4IP	Missense_Mutation	p.Q379E	0.05	0.00	665	139	0.2	Subclonal	-	FALSE
MP11	20	13830890	C	G	SEL1L2	Missense_Mutation	p.A519P	0.04	0.00	493	101	0.17	Subclonal	-	FALSE
MP11	20	33077175	A	G	ITCH	Missense_Mutation	p.D774G	0.15	0.00	507	117	0.62	Subclonal	-	FALSE
MP11	20	49520539	C	G	ADNP	Splice_Site		0.03	0.00	747	131	0.16	Subclonal	-	FALSE
MP11	20	60894838	G	C	LAMA5	Missense_Mutation	p.R2258P	0.05	0.00	277	53	0.27	Subclonal	-	FALSE
MP11	21	28338106	G	C	ADAMTS5	Missense_Mutation	p.A202G	0.05	0.00	619	99	0.24	Subclonal	-	FALSE
MP11	21	30428801	C	T	CCT8	Missense_Mutation	p.D548N	0.03	0.00	663	126	0.16	Subclonal	-	FALSE
MP11	21	46057352	G	T	KRTAP10-10	Missense_Mutation	p.M6I	0.15	0.00	327	124	0.6	Subclonal	LOH	FALSE
MP11	22	20100307	C	G	TRMT2A	Missense_Mutation	p.A553P	0.07	0.00	453	99	0.27	Subclonal	-	FALSE
MP11	22	29469212	C	T	KREMEN1	Missense_Mutation	p.P32S	0.24	0.00	42	12	1	Subclonal	-	FALSE
MP11	22	29704567	C	A	GAS2L1	Missense_Mutation	p.V158M	0.08	0.01	659	125	0.39	Subclonal	-	FALSE
MP11	3	14106332	G	C	TPRXL	Missense_Mutation	p.S219T	0.13	0.00	204	34	0.51	Subclonal	LOH	FALSE
MP11	3	38358003	C	A	SLC22A14	Missense_Mutation	p.S574Y	0.03	0.00	400	78	0.13	Subclonal	LOH	FALSE
MP11	3	46560509	G	T	LRRC2	Missense_Mutation	p.L371I	0.25	0.00	665	143	1	Clonal	LOH	FALSE
MP11	3	48609962	C	T	COL7A1	Missense_Mutation	p.G2304R	0.06	0.00	325	116	0.23	Subclonal	LOH	FALSE
MP11	3	57544858	G	C	DENND6A	Missense_Mutation	p.Q247E	0.03	0.00	632	156	0.13	Subclonal	LOH	FALSE
MP11	3	130110385	C	G	COL6A5	Missense_Mutation	p.E927A	0.09	0.00	672	102	0.43	Subclonal	-	FALSE
MP11	3	160146539	A	G	SMC4	Splice_Site	p.X869_splice	0.15	0.00	547	87	0.74	Subclonal	-	FALSE
MP11	3	164907646	C	T	SLITRK3	Missense_Mutation	p.E329K	0.04	0.01	779	133	0.22	Subclonal	-	FALSE
MP11	3	186338633	C	T	AHSG	Missense_Mutation	p.R340C	0.06	0.00	820	101	0.34	Subclonal	-	FALSE
MP11	3	187416528	C	G	RTP2	Missense_Mutation	p.D146H	0.13	0.00	825	183	1	Clonal	-	FALSE
MP11	4	517332	G	A	PIGG	Missense_Mutation	p.A567T	0.12	0.01	685	150	0.47	Subclonal	-	FALSE
MP11	4	53728751	T	G	RASL11B	Missense_Mutation	p.V26G	0.09	0.00	520	94	0.44	Subclonal	-	FALSE
MP11	4	139157570	T	A	SLC7A11	Nonsense_Mutation	p.K105*	0.05	0.00	734	120	0.23	Subclonal	-	FALSE
MP11	4	183522261	CGTCCAGCTGCAGGACAGCTGG	C	TENM3	In_Frame_Del	p.Q234_V240del	0.10	0.00	585	76	0.39	Subclonal	-	FALSE
MP11	5	7626374	A	G	ADCY2	Missense_Mutation	p.D222G	0.11	0.00	727	88	0.65	Subclonal	-	FALSE
MP11	5	31423011	G	T	DROSHA	Missense_Mutation	p.S1101Y	0.07	0.00	477	77	0.36	Subclonal	-	FALSE
MP11	5	38427218	ACATTTCGGC	A	EGFLAM	In_Frame_Del	p.F641_P643del	0.10	0.00	831	130	0.51	Subclonal	-	FALSE
MP11	5	43492329	T	G	C5orf34	Missense_Mutation	p.E523A	0.04	0.00	821	136	0.18	Subclonal	-	FALSE
MP11	5	171509412	C	T	STK10	Missense_Mutation	p.R636Q	0.05	0.00	574	81	0.24	Subclonal	LOH	FALSE
MP11	5	176707740	A	C	NSD1	Missense_Mutation	p.N1933H	0.28	0.00	770	113	1	Clonal	LOH	FALSE
MP11	6	24706350	GTACTC	G	C6orf62	Frame_Shift_Del		0.10	0.01	958	166	0.51	Subclonal	-	FALSE
MP11	6	31630284	G	C	GPANK1	Missense_Mutation	p.R277Q	0.12	0.00	805	182	0.58	Subclonal	-	FALSE
MP11	6	35420506	C	T	FANCE	Missense_Mutation	p.R62C	0.39	0.00	80	16	1	Clonal	-	FALSE
MP11	6	47253947	T	C	TNFRSF21	Missense_Mutation	p.T161A	0.15	0.00	860	252	0.76	Subclonal	-	FALSE
MP11	6	56501437	G	A	DST	Missense_Mutation	p.A456V	0.20	0.00	812	147	0.99	Clonal	-	FALSE
MP11	6	90604566	G	A	GJA10	Missense_Mutation	p.E127K	0.26	0.00	482	80	1	Clonal	-	FALSE
MP11	6	109480472	A	G	CEP57L1	Missense_Mutation	p.M275V	0.14	0.00	740	130	0.71	Subclonal	-	FALSE
MP11	6	150184567	G	T	LRP11	Missense_Mutation	p.T197S	0.15	0.00	59	8	0.61	Subclonal	LOH	FALSE
MP11	6	152462464	T	C	SYNE1	Missense_Mutation	p.M8374V	0.04	0.00	275	62	0.15	Subclonal	LOH	FALSE
MP11	6	159197457	T	C	EZR	Missense_Mutation	p.I260V	0.04	0.00	423	82	0.14	Subclonal	-	FALSE
MP11	6	163510434	A	G	PACRG	Missense_Mutation	p.M203V	0.06	0.00	554	111	0.25	Subclonal	LOH	FALSE
MP11	6	166843954	A	G	RPS6KA2	Missense_Mutation	p.L531P	0.05	0.00	310	52	0.21	Subclonal	LOH	FALSE
MP11	7	2959173	G	T	CARD11	Missense_Mutation	p.N781K	0.28	0.00	724	137	1	Clonal	LOH	FALSE
MP11	7	24874212	C	G	OSBPL3	Missense_Mutation	p.V547L	0.22	0.00	865	201	0.88	Subclonal	-	FALSE
MP11	7	37780883	G	G	GPR141	Missense_Mutation	p.I296M	0.05	0.00	434	72	0.25	Subclonal	-	FALSE
MP11	7	44805076	C	G	ZMIZ2	Missense_Mutation	p.P714A	0.25	0.00	364	91	1	Clonal	-	FALSE
MP11	7	48411961	T	A	ABCA13	Missense_Mutation	p.L3687H	0.23	0.00	618	111	1	Clonal	-	FALSE
MP11	7	87072675	A	G	ABCB4	Missense_Mutation	p.V439A	0.03	0.00	439	62	0.17	Subclonal	-	FALSE
MP11	7	99769163	G	C	GPC2	Missense_Mutation	p.R437G	0.06	0.00	113	13	0.25	Subclonal	-	FALSE
MP11	7	101960943	A	G	SH2B2	Missense_Mutation	p.Y553C	0.04	0.00	551	130	0.17	Subclonal	-	FALSE
MP11	7	138764855	G	A	ZC3HAV1	Nonsense_Mutation	p.Q278*	0.16	0.00	859	189	0.8	Subclonal	-	FALSE
MP11	7	143053996	C	A	FAM131B	Missense_Mutation	p.A244S	0.07	0.01	659	120	0.33	Subclonal	-	FALSE
MP11	8	48777299	G	A	PRKDC	Missense_Mutation	p.L1798F	0.21	0.00	277	50	1	Clonal	-	FALSE
MP11	8	101075756	C	G	RGS22	Missense_Mutation	p.E414Q	0.04	0.00	805	145	0.25	Subclonal	LOH	FALSE
MP11	8	101724606	G	A	PABPC1	Missense_Mutation	p.T319I	0.04	0.00	273	28	0.22	Subclonal	LOH	FALSE
MP11	8	113348895	A	T	CSMD3	Missense_Mutation	p.D2335E	0.04	0.00	792	76	0.27	Subclonal	-	FALSE
MP11	8	125058478	G	C	FER1L6	Missense_Mutation	p.D1378H	0.14	0.00	795	132	1	Clonal	-	FALSE
MP11	8	142199154	A	G	DENND3	Missense_Mutation	p.N972D	0.04	0.00	762	180	0.21	Subclonal	-	FALSE
MP11	9	6254481	A	C	IL33	Missense_Mutation	p.K180N	0.03	0.00	687	91	0.18	Subclonal	LOH	FALSE
MP11	9	33294762	C	G	NFX1	Missense_Mutation	p.L124V	0.03	0.00	776	222	0.11	Subclonal	LOH	FALSE
MP11	9	72006986	C	G	FAM189A2	Missense_Mutation	p.L407V	0.06	0.00	607	88	0.3	Subclonal	-	FALSE
MP11	9	93983254	G	C	AUH	Missense_Mutation	p.R226G	0.04	0.00	280	37	0.21	Subclonal	-	FALSE
MP11	9	117143390	G	C	AKNA	Missense_Mutation	p.P75R	0.22	0.00	652	114	1	Clonal	-	FALSE
MP11	23	21863354	C	T	MBTPS2	Missense_Mutation	p.T97M	0.04	0.00	725	168	0.19	Subclonal	-	FALSE
MP11	23	36162687	A	G	CHDC2	Missense_Mutation	p.R424G	0.12	0.00	594	91	0.58	Subclonal	-	FALSE
MP11	23	41075588	G	C	USP9X	Missense_Mutation	p.G1923A	0.05	0.00	818	221	0.23	Subclonal	-	FALSE
MP11	23	49142644	C	T	PPP1R3F	Missense_Mutation	p.R498C	0.16	0.00	376	52	0.79	Subclonal	-	FALSE
MP11	23	51641275	A	G	MAGED1	Missense_Mutation	p.N631S	0.06	0.00	678	89	0.3	Subclonal	-	FALSE
MP11	23	53454961	A	G	RIBC1	Missense_Mutation	p.Q43R	0.04	0.00	645	104	0.23	Subclonal	-	FALSE
MP11	23	70328542	T	C	IL2RG	Missense_Mutation	p.N254S	0.03	0.00	576	128	0.13	Subclonal	-	FALSE
MP11	23	108868006	A	T	KCNE1L	Missense_Mutation	p.C72S	0.11	0.00	583	100	0.44	Subclonal	-	FALSE
MP11	23	154185342	G	C	F8	Missense_Mutation	p.L548V	0.05	0.00	757	147	0.25	Subclonal	LOH	FALSE
MP15	1	55518452	G	A	PCSK9	Missense_Mutation	p.G263S	0.15	0.00	142	19	1	Clonal	-	FALSE
MP15	1	65878708	A	T	DNAJC6	Nonstop_Mutation	p.*971Yext*13	0.27	0.00	568	115	1	Clonal	-	FALSE
MP15	1	79402073	A	C	ELTD1	Missense_Mutation	p.S262A	0.08	0.00	346	117	0.55	Subclonal	-	FALSE
MP15	1	92732281	C	T	GLMN	Missense_Mutation	p.M372I	0.25	0.00	65	10	1	Clonal	-	FALSE
MP15	1	94643932	A	G	ARHGAP29	Missense_Mutation	p.Y868H	0.11	0.00	638	119	0.85	Clonal	-	FALSE
MP15	1	149859417	G	C	HIST2H2AB	Missense_Mutation	p.S17W	0.22	0.01	770	143	1	Clonal	-	FALSE
MP15	1	152080991	C	A	TCHH	Nonsense_Mutation	p.E1568*	0.04	0.01	803	179	0.3	Subclonal	-	FALSE
MP15	1	152081012	C	T	TCHH	Missense_Mutation	p.E1561K	0.05	0.01	799	171	0.42	Subclonal	-	FALSE
MP15	1	163317595	C	G	NUF2	Missense_Mutation	p.L331V	0.03	0.00	238	58	0.27	Subclonal	-	FALSE
MP15	1	173569300	GAGAA	G	SLC9C2	Frame_Shift_Del	p.S61Yfs*5	0.19	0.00	499	92	1	Clonal	-	FALSE
MP15	1	182841576	T	G	DHX9	Nonsense_Mutation	p.Y554*	0.25	0.00	466	82	1	Clonal	-	FALSE
MP15	1	200534334	A	G	KIF14	Missense_Mutation	p.C1312R	0.18	0.00	606	102	1	Clonal	-	FALSE
MP15	1	201058389	G	C	CACNA1S	Nonsense_Mutation	p.Y299*	0.07	0.00	672	88	0.62	Subclonal	-	FALSE
MP15	1	202156979	A	C	PTPRVP	Splice_Site		0.07	0.00	234	39	0.65	Subclonal	-	FALSE
MP15	1	205350980	C	T	LEMD1	Missense_Mutation	p.A118T	0.25	0.00	451	62	1	Clonal	-	FALSE
MP15	1	245021342	T	C	HNRNPU	Missense_Mutation	p.K489E	0.14	0.00	462	76	1	Clonal	-	FALSE
MP15	10	6498649	T	C	PRKCQ	Missense_Mutation	p.Y545C	0.03	0.00	150	39	0.2	Subclonal	-	FALSE
MP15	10	31137485	G	C	ZNF438	Missense_Mutation	p.R612G	0.05	0.00	207	63	0.32	Subclonal	-	FALSE
MP15	10	70497646	G	C	CCAR1	Missense_Mutation	p.Q97H	0.07	0.00	453	93	0.55	Subclonal	-	FALSE
MP15	10	71562403	T	A	COL13A1	Missense_Mutation	p.L75Q	0.23	0.00	528	64	1	Clonal	-	FALSE
MP15	10	98708912	T	G	LCOR	Missense_Mutation	p.L33R	0.13	0.00	583	148	0.8	Subclonal	LOH	FALSE
MP15	10	105181191	G	C	PDCD11	Missense_Mutation	p.E788D	0.49	0.00	481	115	1	Clonal	LOH	FALSE
MP15	11	1017273	A	G	MUC6	Missense_Mutation	p.F1843S	0.06	0.01	235	104	0.42	Subclonal	-	FALSE
MP15	11	56949629	C	G	LRRC55	Missense_Mutation	p.R88G	0.08	0.00	779	148	0.59	Subclonal	-	FALSE
MP15	11	64337272	T	G	SLC22A11	Missense_Mutation	p.F511V	0.28	0.00	570	78	1	Clonal	-	FALSE
MP15	11	64559444	G	A	MAP4K2	Missense_Mutation	p.R677C	0.16	0.00	560	87	1	Clonal	-	FALSE
MP15	11	92532877	T	C	FAT3	Missense_Mutation	p.I2233T	0.27	0.00	547	92	1	Clonal	-	FALSE
MP15	11	110454462	C	A	ARHGAP20	Splice_Site	p.X472_splice	0.11	0.00	186	43	0.79	Subclonal	-	FALSE
MP15	11	111745718	C	G	FDXACB1	Missense_Mutation	p.Q601H	0.06	0.00	756	173	0.45	Subclonal	-	FALSE
MP15	11	116718226	C	A	SIK3	Missense_Mutation	p.M1200I	0.12	0.00	318	53	0.86	Clonal	-	FALSE
MP15	12	6833833	A	C	COPS7A	Missense_Mutation	p.E4A	0.15	0.00	226	45	1	Clonal	-	FALSE
MP15	12	10959426	C	A	TAS2R8	Missense_Mutation	p.V52F	0.29	0.00	395	74	1	Clonal	-	FALSE
MP15	12	55615673	A	G	OR10A7	Missense_Mutation	p.I265V	0.31	0.01	708	140	1	Clonal	-	FALSE
MP15	13	42144722	C	G	VWA8	Missense_Mutation	p.D1831H	0.03	0.00	503	141	0.16	Subclonal	LOH	FALSE
MP15	13	49047515	G	T	RB1	Nonsense_Mutation	p.E837*	0.34	0.00	193	90	1	Clonal	LOH	FALSE
MP15	14	57741481	G	A	AP5M1	Nonsense_Mutation	p.W198*	0.36	0.00	537	109	1	Clonal	-	FALSE
MP15	14	71227800	A	T	MAP3K9	Missense_Mutation	p.M307K	0.10	0.00	357	64	0.57	Subclonal	LOH	FALSE
MP15	14	102550258	G	A	HSP90AA1	Nonsense_Mutation	p.Q526*	0.59	0.00	183	41	1	Clonal	LOH	FALSE
MP15	15	40565764	G	C	PAK6	Missense_Mutation	p.D544H	0.08	0.00	765	157	0.54	Subclonal	-	FALSE
MP15	15	42602573	T	C	GANC	Missense_Mutation	p.L272P	0.12	0.00	375	82	0.86	Clonal	-	FALSE
MP15	15	78893619	C	G	CHRNA3	Missense_Mutation	p.M455I	0.13	0.00	538	103	0.89	Clonal	-	FALSE
MP15	15	83240283	C	A	CPEB1	Splice_Site	p.X64_splice	0.09	0.00	508	74	0.63	Subclonal	-	FALSE
MP15	16	614078	C	G	C16orf11	Missense_Mutation	p.P262A	0.14	0.00	455	63	0.97	Clonal	-	FALSE
MP15	16	2314307	C	T	RNPS1	Missense_Mutation	p.D33N	0.07	0.00	728	125	0.47	Subclonal	-	FALSE
MP15	17	7579351	GC	G	TP53	Frame_Shift_Del	p.G112Afs*11	0.43	0.00	603	124	1	Clonal	LOH	FALSE
MP15	17	73872034	C	G	TRIM47	Missense_Mutation	p.Q383H	0.05	0.00	791	132	0.4	Subclonal	-	FALSE
MP15	17	78831644	C	G	RPTOR	Missense_Mutation	p.R485G	0.08	0.00	676	101	0.62	Subclonal	-	FALSE
MP15	18	48333220	C	T	MRO	Missense_Mutation	p.V48I	0.35	0.00	355	88	1	Clonal	-	FALSE
MP15	18	77119409	G	A	ATP9B	Missense_Mutation	p.V987M	0.03	0.00	501	90	0.21	Subclonal	-	FALSE
MP15	19	685915	G	C	PRSS57	Missense_Mutation	p.S218C	0.05	0.00	324	44	0.32	Subclonal	-	FALSE
MP15	19	1047626	A	C	ABCA7	Missense_Mutation	p.T748P	0.27	0.00	407	54	1	Clonal	-	FALSE
MP15	19	5244237	C	G	PTPRS	Missense_Mutation	p.E415D	0.13	0.00	866	141	0.9	Clonal	-	FALSE
MP15	19	38987048	G	C	RYR1	Splice_Site	p.X2222_splice	0.10	0.00	566	61	0.78	Subclonal	-	FALSE
MP15	19	41892579	A	AACGGAAG	EXOSC5	Frame_Shift_Ins	p.F223Lfs*47	0.19	0.00	898	115	1	Clonal	-	FALSE
MP15	19	42482833	G	T	ATP1A3	Missense_Mutation	p.P532T	0.03	0.00	703	86	0.24	Subclonal	-	FALSE
MP15	19	42746467	C	G	GSK3A	Missense_Mutation	p.A51P	0.23	0.00	109	21	1	Clonal	-	FALSE
MP15	19	58773717	G	T	ZNF544	Missense_Mutation	p.G582V	0.09	0.02	401	66	0.65	Subclonal	-	FALSE
MP15	2	1652299	C	A	PXDN	Missense_Mutation	p.D1085Y	0.10	0.00	838	136	0.82	Subclonal	-	FALSE
MP15	2	11800227	G	C	NTSR2	Missense_Mutation	p.L311V	0.19	0.00	770	114	1	Clonal	-	FALSE
MP15	2	179463992	G	C	TTN	Missense_Mutation	p.T18843R	0.28	0.00	240	57	1	Clonal	-	FALSE
MP15	2	187697887	C	A	ZSWIM2	Nonsense_Mutation	p.E301*	0.10	0.00	83	19	0.68	Subclonal	-	FALSE
MP15	2	219129800	C	T	AAMP	Missense_Mutation	p.R391H	0.33	0.02	393	46	1	Clonal	-	FALSE
MP15	2	220197896	C	G	RESP18	Missense_Mutation	p.A2P	0.05	0.00	438	116	0.35	Subclonal	-	FALSE
MP15	2	233633081	AGATGCAAAACAGT	A	KCNJ13	Frame_Shift_Del	p.H297Lfs*3	0.20	0.00	892	218	1	Clonal	-	FALSE
MP15	20	30064571	T	C	REM1	Missense_Mutation	p.L108P	0.13	0.00	197	28	0.93	Clonal	-	FALSE
MP15	20	30689267	G	C	HCK	Missense_Mutation	p.S509T	0.11	0.00	748	126	0.78	Subclonal	-	FALSE
MP15	20	44472181	C	T	ACOT8	Missense_Mutation	p.E276K	0.27	0.00	788	128	1	Clonal	-	FALSE
MP15	21	46925331	G	C	COL18A1	Missense_Mutation	p.G1205R	0.08	0.00	817	171	0.57	Subclonal	-	FALSE
MP15	21	47406554	C	G	COL6A1	Missense_Mutation	p.H181Q	0.32	0.01	487	69	1	Clonal	-	FALSE
MP15	22	31494826	G	C	SMTN	Missense_Mutation	p.G778A	0.05	0.00	301	25	0.48	Subclonal	-	FALSE
MP15	22	41278068	G	A	XPNPEP3	Missense_Mutation	p.S159N	0.08	0.00	828	175	0.73	Subclonal	-	FALSE
MP15	3	52393993	C	T	DNAH1	Missense_Mutation	p.S1490L	0.10	0.00	249	51	0.5	Subclonal	LOH	FALSE
MP15	3	52422309	C	T	DNAH1	Missense_Mutation	p.H3044Y	0.13	0.00	712	142	0.64	Subclonal	LOH	FALSE
MP15	3	87039784	G	C	VGLL3	Missense_Mutation	p.P37R	0.11	0.00	157	16	0.98	Clonal	-	FALSE
MP15	3	130360495	G	C	COL6A6	Splice_Site	p.X1698_splice	0.48	0.00	58	15	1	Subclonal	-	FALSE
MP15	3	134920330	G	C	EPHB1	Missense_Mutation	p.Q715H	0.17	0.00	710	92	1	Clonal	-	FALSE
MP15	3	142840937	G	T	CHST2	Missense_Mutation	p.D427Y	0.03	0.00	775	76	0.24	Subclonal	-	FALSE
MP15	3	183906604	A	C	ABCF3	Missense_Mutation	p.I295L	0.07	0.01	855	173	0.79	Subclonal	-	FALSE
MP15	3	194158138	C	T	ATP13A3	Missense_Mutation	p.R634H	0.60	0.00	774	186	1	Clonal	-	FALSE
MP15	4	52861415	C	A	LRRC66	Missense_Mutation	p.M591I	0.22	0.00	610	130	1	Clonal	-	FALSE
MP15	4	55956161	C	T	KDR	Missense_Mutation	p.D1052N	0.13	0.00	372	111	0.81	Subclonal	-	FALSE
MP15	4	77700095	A	G	SHROOM3	Missense_Mutation	p.Q1919R	0.30	0.00	375	40	1	Clonal	LOH	FALSE
MP15	4	96127900	C	A	UNC5C	Missense_Mutation	p.C594F	0.10	0.00	246	40	0.68	Subclonal	LOH	FALSE
MP15	4	141294703	G	A	SCOC	Missense_Mutation	p.V5I	0.20	0.00	628	111	1	Clonal	-	FALSE
MP15	4	148578976	C	T	PRMT10	Missense_Mutation	p.E433K	0.09	0.00	331	89	0.54	Subclonal	-	FALSE
MP15	4	187549421	G	A	FAT1	Missense_Mutation	p.S1566F	0.05	0.00	589	112	0.37	Subclonal	LOH	FALSE
MP15	5	5460712	C	G	KIAA0947	Nonsense_Mutation	p.S422*	0.31	0.00	512	116	1	Clonal	-	FALSE
MP15	5	32263325	C	T	MTMR12	Missense_Mutation	p.V203I	0.11	0.00	428	94	0.79	Subclonal	-	FALSE
MP15	5	33947344	AACAGGCTGC	A	SLC45A2	In_Frame_Del	p.C428_L430del	0.20	0.00	590	124	1	Clonal	-	FALSE
MP15	5	67591246	A	C	PIK3R1	Splice_Site	p.X582_splice	0.15	0.00	497	163	0.75	Subclonal	LOH	FALSE
MP15	5	126770382	G	A	MEGF10	Missense_Mutation	p.C615Y	0.27	0.01	731	117	1	Clonal	-	FALSE
MP15	5	161580138	C	G	GABRG2	Missense_Mutation	p.Q438E	0.11	0.00	212	57	0.54	Subclonal	LOH	FALSE
MP15	6	30126400	C	T	TRIM10	Missense_Mutation	p.V178M	0.34	0.00	613	114	1	Clonal	-	FALSE
MP15	6	35211445	G	A	SCUBE3	Missense_Mutation	p.E882K	0.07	0.00	639	67	0.54	Subclonal	-	FALSE
MP15	6	39855284	A	T	DAAM2	Missense_Mutation	p.D659V	0.09	0.00	723	128	0.75	Subclonal	-	FALSE
MP15	6	46660489	G	C	TDRD6	Missense_Mutation	p.E1542Q	0.32	0.00	742	126	1	Clonal	-	FALSE
MP15	6	83858486	T	A	DOPEY1	Missense_Mutation	p.V1914E	0.46	0.00	71	23	1	Clonal	LOH	FALSE
MP15	6	107390485	C	A	BEND3	Missense_Mutation	p.G637V	0.07	0.01	768	120	0.65	Subclonal	-	FALSE
MP15	6	121615715	C	T	TBC1D32	Splice_Site	p.X411_splice	0.05	0.00	364	70	0.45	Subclonal	-	FALSE
MP15	6	136599813	C	G	BCLAF1	Missense_Mutation	p.R69P	0.11	0.00	784	231	1	Clonal	-	FALSE
MP15	7	4821332	C	G	AP5Z1	Missense_Mutation	p.H105D	0.11	0.00	773	154	0.96	Clonal	-	FALSE
MP15	7	31682343	AAAGCCAAG	A	CCDC129	Frame_Shift_Del	p.K460Rfs*24	0.33	0.00	1342	224	1	Clonal	-	FALSE
MP15	7	86990806	GGCC	G	CROT	In_Frame_Del	p.W142_P143delinsC	0.25	0.00	741	188	1	Clonal	-	FALSE
MP15	7	103163856	C	A	RELN	Missense_Mutation	p.C2491F	0.25	0.00	144	37	1	Clonal	-	FALSE
MP15	8	17851038	A	C	PCM1	Missense_Mutation	p.E1579D	0.08	0.00	520	146	0.54	Subclonal	-	FALSE
MP15	8	61655654	C	T	CHD7	Nonsense_Mutation	p.Q555*	0.27	0.00	164	49	1	Clonal	-	FALSE
MP15	8	103573033	A	G	ODF1	Missense_Mutation	p.N225S	0.12	0.00	827	116	1	Clonal	-	FALSE
MP15	8	110488822	A	G	PKHD1L1	Missense_Mutation	p.N2948S	0.04	0.00	750	79	0.64	Subclonal	-	FALSE
MP15	8	144994352	A	T	PLEC	Missense_Mutation	p.S3350T	0.43	0.00	808	76	1	Clonal	-	FALSE
MP15	9	20819835	G	C	FOCAD	Missense_Mutation	p.R499T	0.09	0.00	160	42	0.7	Subclonal	-	FALSE
MP15	23	18528970	A	C	CDKL5	Missense_Mutation	p.H32P	0.33	0.00	480	97	1	Clonal	-	FALSE
MP15	23	22117241	T	A	PHEX	Missense_Mutation	p.F351I	0.04	0.00	781	274	0.33	Subclonal	-	FALSE
MP15	23	24006600	C	G	KLHL15	Missense_Mutation	p.G418A	0.32	0.01	767	160	1	Clonal	-	FALSE
MP15	23	78010601	A	G	LPAR4	Missense_Mutation	p.T79A	0.08	0.00	727	165	0.53	Subclonal	-	FALSE
MP15	23	149638397	CA	C	MAMLD1	Frame_Shift_Del	p.I161Ffs*9	0.31	0.00	523	95	1	Clonal	-	FALSE
MP17	1	36921849	G	C	MRPS15	Missense_Mutation	p.P192R	0.12	0.00	650	88	0.38	Subclonal	-	FALSE
MP17	1	186157102	C	T	HMCN1	Missense_Mutation	p.T5501I	0.31	0.00	820	192	1	Clonal	-	FALSE
MP17	10	120833354	G	A	EIF3A	Missense_Mutation	p.P49S	0.03	0.00	841	202	0.09	Subclonal	-	FALSE
MP17	11	107506403	G	T	ELMOD1	Missense_Mutation	p.G111V	0.08	0.00	582	73	0.26	Subclonal	-	FALSE
MP17	11	113085211	G	A	NCAM1	Missense_Mutation	p.R405Q	0.10	0.00	245	35	0.32	Subclonal	-	FALSE
MP17	11	118501943	C	A	PHLDB1	Missense_Mutation	p.T616N	0.32	0.00	654	161	1	Clonal	-	FALSE
MP17	12	49445040	TG	T	KMT2D	Frame_Shift_Del	p.Q809Rfs*121	0.32	0.00	1160	274	1	Clonal	-	FALSE
MP17	12	105478169	G	C	ALDH1L2	Missense_Mutation	p.R16G	0.19	0.00	123	36	0.61	Subclonal	-	FALSE
MP17	13	24413811	G	GA	MIPEP	Frame_Shift_Ins	p.Q439Sfs*50	0.24	0.00	466	92	0.79	Subclonal	-	FALSE
MP17	16	2867280	G	A	PRSS21	Missense_Mutation	p.R4H	0.08	0.00	40	14	0.24	Subclonal	-	FALSE
MP17	16	61687612	G	T	CDH8	Nonsense_Mutation	p.S767*	0.24	0.01	791	113	0.78	Subclonal	-	FALSE
MP17	16	67916765	G	A	EDC4	Missense_Mutation	p.G1209D	0.27	0.00	588	139	0.89	Subclonal	-	FALSE
MP17	17	7577609	C	T	TP53	Splice_Site	p.X225_splice	0.14	0.00	391	66	0.47	Subclonal	-	FALSE
MP17	17	7578413	C	A	TP53	Missense_Mutation	p.V173L	0.27	0.00	774	154	0.87	Subclonal	-	TRUE

MP17	18	10696074	C	G	PIEZO2	Missense_Mutation	p.E2283D	0.16	0.01	437	85	0.52	Subclonal	.	FALSE
MP17	19	15271708	TA	T	NOTCH3	Frame_Shift_Del	p.Y2244Tfs*2	0.31	0.00	463	107	1	Clonal	.	FALSE
MP17	19	40360933	C	T	FCGBP	Missense_Mutation	p.D5159N	0.04	0.00	596	83	0.13	Subclonal	.	FALSE
MP17	19	44892153	G	C	ZNF285	Missense_Mutation	p.T85S	0.04	0.00	136	15	0.12	Subclonal	.	FALSE
MP17	19	56467135	G	A	NLRP8	Missense_Mutation	p.V571M	0.29	0.00	677	112	0.96	Clonal	.	FALSE
MP17	2	37486764	C	A	PRKD3	Nonsense_Mutation	p.E703*	0.04	0.00	732	128	0.12	Subclonal	.	FALSE
MP17	2	45879452	CGATGTGTGCAACGGACGCAA	C	PRKCE	Frame_Shift_Del	p.V73Rfs*24	0.12	0.00	702	127	0.38	Subclonal	.	FALSE
MP17	2	65243617	G	T	SLC1A4	Missense_Mutation	p.V282L	0.18	0.00	538	83	0.58	Subclonal	.	FALSE
MP17	2	152355874	T	A	NEB	Missense_Mutation	p.I8018F	0.05	0.00	396	83	0.17	Subclonal	.	FALSE
MP17	20	57484420	C	T	GNAS	Missense_Mutation	p.R201C	0.29	0.00	826	150	0.94	Clonal	.	TRUE
MP17	21	22841072	C	T	NCAM2	Missense_Mutation	p.P622S	0.22	0.00	356	56	0.73	Subclonal	.	FALSE
MP17	3	178952085	A	G	PIK3CA	Missense_Mutation	p.H1047R	0.29	0.00	816	118	0.95	Clonal	.	TRUE
MP17	4	126389828	G	A	FAT4	Missense_Mutation	p.D4021N	0.26	0.00	791	167	0.86	Subclonal	.	FALSE
MP17	5	157073647	A	G	SOX30	Missense_Mutation	p.V462A	0.26	0.00	255	33	0.84	Subclonal	.	FALSE
MP17	6	33262762	G	A	RGL2	Missense_Mutation	p.R424W	0.20	0.00	131	28	0.65	Subclonal	.	FALSE
MP17	6	43634191	C	A	RSPH9	Missense_Mutation	p.T219K	0.31	0.00	580	100	1	Clonal	.	FALSE
MP17	7	142566754	C	A	EPHB6	Missense_Mutation	p.Q771K	0.06	0.00	194	38	0.18	Subclonal	.	FALSE
MP17	7	158581071	G	A	ESYT2	Nonsense_Mutation	p.R256*	0.42	0.03	269	61	1	Clonal	.	FALSE
MP17	9	100899889	C	A	CORO2A	Missense_Mutation	p.D95Y	0.05	0.00	411	92	0.1	Subclonal	LOH	FALSE
MP17	23	18646512	C	T	CDKL5	Missense_Mutation	p.R840C	0.04	0.01	795	145	0.12	Subclonal	.	FALSE
MP18	1	897796	C	T	KLHL17	Missense_Mutation	p.A258V	0.03	0.00	746	190	0.12	Subclonal	.	FALSE
MP18	1	11575448	TGCCAG	T	PTCHD2	Frame_Shift_Del	p.A493Hfs*85	0.08	0.00	309	72	0.29	Subclonal	.	FALSE
MP18	1	21099997	T	A	HP1BP3	Missense_Mutation	p.M153L	0.24	0.00	342	136	0.82	Subclonal	.	FALSE
MP18	1	23889612	T	C	ZNF436	Missense_Mutation	p.E88G	0.09	0.00	773	260	0.3	Subclonal	.	FALSE
MP18	1	37356614	T	A	GRIK3	Missense_Mutation	p.I67F	0.03	0.00	707	136	0.11	Subclonal	.	FALSE
MP18	1	85560213	G	A	WDR63	Missense_Mutation	p.S383N	0.04	0.00	421	124	0.13	Subclonal	.	FALSE
MP18	1	152382620	G	A	CRNN	Missense_Mutation	p.T313I	0.10	0.00	782	128	0.45	Subclonal	.	FALSE
MP18	1	156023175	C	T	DCST1	Missense_Mutation	p.A651V	0.14	0.00	310	89	0.46	Subclonal	.	FALSE
MP18	1	156304534	C	T	CCT3	Missense_Mutation	p.M59I	0.08	0.00	556	121	0.37	Subclonal	.	FALSE
MP18	1	196967323	G	A	CFHR5	Missense_Mutation	p.G346R	0.07	0.00	122	46	0.29	Subclonal	.	FALSE
MP18	1	205210909	A	C	TMCC2	Missense_Mutation	p.K162Q	0.09	0.01	620	133	0.4	Subclonal	.	FALSE
MP18	1	226553708	C	A	PARP1	Missense_Mutation	p.V818F	0.04	0.00	427	84	0.18	Subclonal	.	FALSE
MP18	1	228559243	C	T	OBSCN	Missense_Mutation	p.R7879C	0.24	0.00	378	55	1	Clonal	.	FALSE
MP18	1	247835605	G	A	OR13G1	Missense_Mutation	p.L247F	0.09	0.00	253	46	0.51	Subclonal	.	FALSE
MP18	10	12077197	C	G	UPF2	Missense_Mutation	p.D76H	0.18	0.00	624	136	0.78	Subclonal	.	FALSE
MP18	10	98807456	G	C	SLIT1	Missense_Mutation	p.T542R	0.18	0.00	88	23	0.44	Subclonal	LOH	FALSE
MP18	11	8940938	G	A	AKIP1	Missense_Mutation	p.V182I	0.18	0.00	337	104	0.61	Subclonal	.	FALSE
MP18	11	60899812	G	A	VPS37C	Missense_Mutation	p.P183L	0.04	0.00	300	53	0.13	Subclonal	.	FALSE
MP18	11	62600461	C	G	WDR74	Missense_Mutation	p.R374P	0.14	0.00	422	120	0.34	Subclonal	.	FALSE
MP18	11	117299142	C	A	DSCAML1	Missense_Mutation	p.G2082C	0.14	0.02	657	127	0.61	Subclonal	.	FALSE
MP18	11	118183392	T	C	CD3E	Missense_Mutation	p.S55P	0.32	0.00	617	177	1	Clonal	.	FALSE
MP18	11	118769127	G	T	BCL9L	Missense_Mutation	p.F1499L	0.29	0.00	348	59	0.98	Clonal	.	FALSE
MP18	11	124765535	C	T	ROBO4	Missense_Mutation	p.R285K	0.13	0.01	581	115	0.45	Subclonal	.	FALSE
MP18	12	57628139	C	T	SHMT2	Missense_Mutation	p.H504Y	0.39	0.00	399	179	0.93	Clonal	LOH	FALSE
MP18	12	100491231	G	C	UHRF1BP1L	Missense_Mutation	p.A194G	0.23	0.00	189	61	0.78	Subclonal	.	FALSE
MP18	12	124358132	C	T	DNAH10	Missense_Mutation	p.R2487C	0.12	0.00	541	118	0.41	Subclonal	.	FALSE
MP18	12	130898843	C	T	RIMBP2	Missense_Mutation	p.G827R	0.07	0.00	346	46	0.31	Subclonal	.	FALSE
MP18	14	55646413	G	C	DLGAP5	Missense_Mutation	p.N236K	0.06	0.00	176	100	0.14	Subclonal	LOH	FALSE
MP18	15	22969241	G	C	CYFIP1	Missense_Mutation	p.A823P	0.24	0.00	620	120	0.8	Subclonal	.	FALSE
MP18	15	30011250	GTCTGGCC	G	TJP1	Frame_Shift_Del	p.R1030Tfs*6	0.25	0.01	484	167	0.6	Subclonal	.	FALSE
MP18	15	43489654	G	A	EPB42	Missense_Mutation	p.S671L	0.10	0.00	374	134	0.25	Subclonal	.	FALSE
MP18	15	43815854	C	A	MAP1A	Missense_Mutation	p.A728D	0.29	0.00	264	111	0.7	Subclonal	.	FALSE
MP18	15	85401679	A	T	ALPK3	Missense_Mutation	p.D1439V	0.06	0.00	136	34	0.2	Subclonal	.	FALSE
[illegible]															

Supplementary Table S2 - Page 3 of 5

MPB	19	44739247	G	A	[illegible]
MPB	19	50380303	G	C	[illegible]
MPB	2	27445104	G	C	[illegible]
MPB	2	138373741	G	C	[illegible]
MPB	2	233346217	C	T	[illegible]
MPB	20	31021570	G	C	[illegible]
MPB	20	34542094	G	T	[illegible]
MPB	20	36012629	C	T	[illegible]
MPB	20	58547038	A	C	[illegible]
MPB	20	60868821	G	C	[illegible]
MPB	20	60885319	C	G	[illegible]
MPB	20	62853283	C	G	[illegible]
MPB	21	45457686	G	A	[illegible]
MPB	22	26164094	T	C	[illegible]
MPB	22	46447728	A	T	[illegible]
MPB	3	14106332	G	C	[illegible]
MPB	3	107435463	C	T	[illegible]
MPB	3	182597394	C	G	[illegible]
MPB	3	195605762	A	G	[illegible]
MPB	4	8229535	G	A	[illegible]
MPB	4	77065354	C	G	[illegible]
MPB	4	109584391	G	A	[illegible]
MPB	4	148743974	G	C	[illegible]
MPB	6	30039022	C	T	[illegible]
MPB	6	32551915	A	G	[illegible]
MPB	6	57011988	C	A	[illegible]
MPB	6	73017056	C	T	[illegible]
MPB	6	139186175	C	G	[illegible]
MPB	6	152652506	A	T	[illegible]
MPB	6	152765580	A	T	[illegible]
MPB	7	14722196	A	C	[illegible]
MPB	7	22769261	C	G	[illegible]
MPB	7	23826149	C	A	[illegible]
MPB	7	31123783	C	T	[illegible]
MPB	7	73452042	G	C	[illegible]
MPB	7	131194993	A	G	[illegible]
MPB	7	134819837	T	C	[illegible]
MPB	8	17486053	C	G	[illegible]
MPB	8	22863630	G	A	[illegible]
MPB	8	67476997	G	C	[illegible]
MPB	8	101075782	C	G	[illegible]
MPB	8	104427096	C	G	[illegible]
MPB	8	144885585	C	T	[illegible]
MPB	9	27192621	G	A	[illegible]
MPB	9	72724635	G	C	[illegible]
MPB	9	133759709	G	A	[illegible]
MPB	23	15342986	C	G	[illegible]
MPB	23	21627435	G	A	[illegible]
MPB	23	83128170	A	T	[illegible]
MPB	23	108846459	A	ACAGTAG	[illegible]
MPB	23	123870839	C	G	[illegible]
MPB	23	153633824	A	C	[illegible]
MPB	23	153663804	G	T	[illegible]
MTC01	1	22150863	C	T	[illegible]
MTC01	1	154541995	G	A	[illegible]
MTC01	10	105344611	G	A	[illegible]
MTC01	11	67257924	C	G	[illegible]
MTC01	11	119535659	C	T	[illegible]
MTC01	12	25380282	G	C	[illegible]
MTC01	12	53691661	G	A	[illegible]
MTC01	13	50141301	C	T	[illegible]

Supplementary Table S2 - Page 4 of 5

MTC07	16	23711917	T	G	ERN2	Missense_Mutation	p.S538R	0.19	0.00	261	71	1	Clonal	.	FALSE
MTC07	16	48244966	C	G	ABCC11	Missense_Mutation	p.G501R	0.28	0.00	447	116	1	Clonal	.	FALSE
MTC07	16	89167477	C	T	ACSF3	Missense_Mutation	p.P130S	0.05	0.00	296	140	0.33	Subclonal	LOH	FALSE
MTC07	16	90130139	A	G	PRDM7	Missense_Mutation	p.L130S	0.04	0.00	228	67	0.22	Subclonal	LOH	FALSE
MTC07	17	3524560	T	C	SHPK	Missense_Mutation	p.Y265C	0.05	0.00	85	35	0.24	Subclonal	LOH	FALSE
MTC07	17	7366348	A	C	ZBTB4	Missense_Mutation	p.D651E	0.03	0.00	262	101	0.18	Subclonal	LOH	FALSE
MTC07	17	7578265	A	G	TP53	Missense_Mutation	p.I195T	0.04	0.00	185	76	0.19	Subclonal	LOH	TRUE
MTC07	17	7578271	T	A	TP53	Missense_Mutation	p.H193L	0.42	0.00	199	80	1	Clonal	LOH	TRUE
MTC07	17	16347245	C	T	FAM211A	Missense_Mutation	p.R231H	0.04	0.00	153	56	0.2	Subclonal	LOH	FALSE
MTC07	17	27829701	G	A	TAOK1	Missense_Mutation	p.R433H	0.38	0.00	228	79	1	Clonal	LOH	FALSE
MTC07	17	36667150	C	T	ARHGAP23	Missense_Mutation	p.S1473F	0.34	0.00	108	48	1	Clonal	LOH	FALSE
MTC07	17	48166489	C	T	ITGA3	Missense_Mutation	p.R1021W	0.04	0.00	345	116	0.25	Subclonal	LOH	FALSE
MTC07	17	55195780	A	C	AKAP1	Missense_Mutation	p.S847R	0.04	0.00	305	80	0.22	Subclonal	LOH	FALSE
MTC07	17	56833491	T	C	PPM1E	Missense_Mutation	p.S45P	0.03	0.00	641	163	0.2	Subclonal	LOH	FALSE
MTC07	17	67031907	A	G	ABCA9	Missense_Mutation	p.L279P	0.03	0.00	456	115	0.2	Subclonal	LOH	FALSE
MTC07	17	73499946	C	T	CASKIN2	Missense_Mutation	p.R522H	0.08	0.00	263	68	0.54	Subclonal	LOH	FALSE
MTC07	17	73569706	G	A	LLGL2	Missense_Mutation	p.R957Q	0.06	0.00	124	43	0.4	Subclonal	LOH	FALSE
MTC07	17	74018031	G	T	EVPL	Missense_Mutation	p.L242M	0.05	0.00	245	74	0.35	Subclonal	LOH	FALSE
MTC07	17	74155497	G	A	RNF157	Missense_Mutation	p.P408L	0.04	0.00	309	93	0.3	Subclonal	LOH	FALSE
MTC07	17	77807927	A	G	CBX4	Missense_Mutation	p.V505A	0.22	0.00	290	121	1	Clonal	LOH	FALSE
MTC07	17	78272230	C	G	RNF213	Missense_Mutation	p.H708D	0.05	0.00	350	96	0.37	Subclonal	LOH	FALSE
MTC07	17	79419058	G	A	RP11-1055B8.7	Missense_Mutation	p.R1414Q	0.44	0.00	90	22	1	Subclonal	LOH	FALSE
MTC07	18	30275459	C	T	KLHL14	Missense_Mutation	p.V376M	0.35	0.00	237	77	1	Clonal	.	FALSE
MTC07	18	30321910	C	G	KLHL14	Missense_Mutation	p.K350N	0.03	0.00	209	72	0.21	Subclonal	.	FALSE
MTC07	18	42531593	C	G	SETBP1	Missense_Mutation	p.S763C	0.35	0.00	367	193	1	Clonal	LOH	FALSE
MTC07	19	12786449	G	A	WDR83	Missense_Mutation	p.R304Q	0.05	0.00	244	72	0.25	Subclonal	LOH	FALSE
MTC07	19	12921186	T	C	RNASEH2A	Missense_Mutation	p.L202S	0.03	0.00	283	95	0.16	Subclonal	LOH	FALSE
MTC07	19	15233732	G	A	ILVBL	Missense_Mutation	p.A192V	0.03	0.00	459	123	0.27	Subclonal	.	FALSE
MTC07	19	38643539	C	T	SIPA1L3	Missense_Mutation	p.T1198M	0.06	0.00	375	174	0.39	Subclonal	.	FALSE
MTC07	19	42560859	C	T	GRIK5	Missense_Mutation	p.M286I	0.05	0.00	168	49	0.33	Subclonal	.	FALSE
MTC07	19	44536850	C	A	ZNF222	Nonsense_Mutation	p.Y381*	0.06	0.00	455	147	0.36	Subclonal	.	FALSE
MTC07	19	44636802	A	T	ZNF225	Missense_Mutation	p.T679S	0.05	0.00	169	47	0.29	Subclonal	.	FALSE
MTC07	19	46094229	T	A	GPR4	Missense_Mutation	p.K299M	0.24	0.00	291	103	1	Subclonal	.	FALSE
MTC07	19	46333349	C	T	SYMPK	Missense_Mutation	p.R571Q	0.04	0.00	167	44	0.26	Subclonal	.	FALSE
MTC07	19	47423849	AAT	A	ARHGAP35	Frame_Shift_Del	p.I640Rfs*42	0.17	0.00	410	140	1	Clonal	.	FALSE
MTC07	19	50193725	C	T	ADM5	Missense_Mutation	p.P146L	0.28	0.00	208	80	1	Clonal	.	FALSE
MTC07	19	51919998	T	C	SIGLEC10	Missense_Mutation	p.N210D	0.04	0.00	189	60	0.23	Subclonal	.	FALSE
MTC07	19	54314512	C	T	NLRP12	Missense_Mutation	p.R134H	0.19	0.00	273	97	1	Clonal	.	FALSE
MTC07	19	58879660	T	C	ZNF837	Missense_Mutation	p.Y347C	0.09	0.00	90	25	0.55	Subclonal	.	FALSE
MTC07	2	3391539	G	A	TRAPPC12	Missense_Mutation	p.E49K	0.08	0.00	647	249	0.51	Subclonal	.	FALSE
MTC07	2	21227979	C	T	APOB	Missense_Mutation	p.V3921I	0.07	0.00	181	58	0.41	Subclonal	.	FALSE
MTC07	2	31590917	T	C	XDH	Missense_Mutation	p.I703V	0.03	0.00	230	80	0.16	Subclonal	.	FALSE
MTC07	2	80085198	C	T	CTNNA2	Missense_Mutation	p.R120C	0.10	0.00	117	35	0.53	Subclonal	.	FALSE
MTC07	2	97268597	C	T	KANSL3	Missense_Mutation	p.A718T	0.04	0.00	271	86	0.23	Subclonal	.	FALSE
MTC07	2	101911488	T	C	RNF149	Missense_Mutation	p.I206V	0.03	0.00	266	76	0.17	Subclonal	.	FALSE
MTC07	2	112786211	C	T	MERTK	Missense_Mutation	p.P924S	0.03	0.00	458	169	0.17	Subclonal	.	FALSE
MTC07	2	121747822	G	A	GLI2	Missense_Mutation	p.M1444I	0.05	0.00	371	159	0.26	Subclonal	.	FALSE
MTC07	2	121747823	C	T	GLI2	Missense_Mutation	p.L1445F	0.05	0.00	375	156	0.26	Subclonal	.	FALSE
MTC07	2	179576726	G	T	TTN	Nonsense_Mutation	p.C9277*	0.26	0.00	228	80	1	Clonal	.	FALSE
MTC07	2	187501806	G	C	ITGAV	Missense_Mutation	p.V258L	0.13	0.00	344	150	0.78	Subclonal	.	FALSE
MTC07	2	214013322	G	C	IKZF2	Missense_Mutation	p.T11R	0.16	0.00	231	74	0.96	Subclonal	.	FALSE
MTC07	2	228172468	G	C	COL4A3	Missense_Mutation	p.R1432P	0.06	0.00	128	35	0.38	Subclonal	.	FALSE
MTC07	2	230450693	G	A	DNER	Missense_Mutation	p.T243M	0.03	0.00	270	87	0.2	Subclonal	.	FALSE
MTC07	2	238283472	T	G	COL6A3	Missense_Mutation	p.K1088Q	0.04	0.00	571	220	0.23	Subclonal	.	FALSE
MTC07	2	238726904	G	A	RBM44	Missense_Mutation	p.E449K	0.04	0.00	332	93	0.24	Subclonal	.	FALSE
MTC07	20	3846401	G	C	MAVS	Missense_Mutation	p.E410D	0.12	0.00	182	86	0.59	Subclonal	LOH	FALSE
MTC07	20	29960672	A	G	DEFB118	Missense_Mutation	p.E24G	0.29	0.00	177	53	1	Clonal	.	FALSE
MTC07	20	61288379	C	A	SLCO4A1	Missense_Mutation	p.H191Q	0.12	0.00	198	56	0.83	Subclonal	.	FALSE
MTC07	22	20761192	G	T	ZNF74	Missense_Mutation	p.K623N	0.04	0.00	334	116	0.24	Subclonal	LOH	FALSE
MTC07	22	20761193	C	T	ZNF74	Missense_Mutation	p.L624F	0.03	0.00	336	119	0.2	Subclonal	LOH	FALSE
MTC07	22	32200849	C	T	DEPDC5	Missense_Mutation	p.R389C	0.04	0.00	170	70	0.22	Subclonal	LOH	FALSE
MTC07	22	50688571	G	C	HDAC10	Missense_Mutation	p.R104G	0.03	0.00	230	94	0.21	Subclonal	LOH	FALSE
MTC07	22	51042973	G	A	MAPK8IP2	Missense_Mutation	p.A415T	0.10	0.00	50	22	0.61	Subclonal	LOH	FALSE
MTC07	3	13367357	G	A	NUP210	Missense_Mutation	p.R1528W	0.04	0.00	183	72	0.22	Subclonal	LOH	FALSE
MTC07	3	38135170	A	G	DLEC1	Missense_Mutation	p.T611A	0.06	0.00	252	88	0.34	Subclonal	LOH	FALSE
MTC07	3	55508550	C	T	WNT5A	Missense_Mutation	p.A167T	0.15	0.00	161	40	0.77	Subclonal	LOH	FALSE
MTC07	3	57407306	G	C	DNAH12	Missense_Mutation	p.S1815C	0.33	0.00	203	80	1	Clonal	LOH	FALSE
MTC07	3	64507857	C	T	ADAMTS9	Missense_Mutation	p.R1933Q	0.03	0.00	360	153	0.17	Subclonal	LOH	FALSE
MTC07	3	64580068	C	T	ADAMTS9	Missense_Mutation	p.V1408I	0.03	0.00	381	166	0.18	Subclonal	LOH	FALSE
MTC07	3	69031963	T	C	EOGT	Missense_Mutation	p.R321G	0.07	0.00	180	59	0.34	Subclonal	LOH	FALSE
MTC07	3	137843431	C	T	A4GNT	Missense_Mutation	p.R233K	0.21	0.00	487	170	1	Clonal	.	FALSE
MTC07	3	138664847	C	T	FOXL2	Missense_Mutation	p.G240S	0.05	0.00	333	89	0.28	Subclonal	.	FALSE
MTC07	4	126370647	G	A	FAT4	Missense_Mutation	p.D2826N	0.03	0.00	394	147	0.16	Subclonal	.	FALSE
MTC07	4	144618119	C	T	FREM3	Missense_Mutation	p.R1237Q	0.03	0.00	392	147	0.17	Subclonal	.	FALSE
MTC07	4	156784678	C	A	ASIC5	Missense_Mutation	p.W90L	0.10	0.00	393	162	0.61	Subclonal	.	FALSE
MTC07	5	205939	C	T	CCDC127	Missense_Mutation	p.A86T	0.04	0.00	613	181	0.22	Subclonal	.	FALSE
MTC01	5	1295228	G	A	TERT	Promoter		0.49	0.00	82	126	1	Clonal	.	TRUE
MP17	5	1295228	G	A	TERT	Promoter		0.40	0.00	67	346	1	Clonal	.	TRUE
MTC07	5	1271301	C	G	TERT	Missense_Mutation	p.A801P	0.09	0.00	349	104	0.54	Subclonal	.	FALSE
MTC07	5	149450132	T	C	CSF1R	Missense_Mutation	p.H362R	0.05	0.00	200	74	0.28	Subclonal	LOH	FALSE
MTC07	5	156899928	C	T	NIPAL4	Missense_Mutation	p.S454L	0.45	0.00	413	149	1	Clonal	LOH	FALSE
MTC07	5	177547537	A	G	N4BP3	Missense_Mutation	p.K230R	0.05	0.00	400	156	0.29	Subclonal	LOH	FALSE
MTC07	6	4995505	T	C	RPP40	Missense_Mutation	p.Y300C	0.04	0.00	216	69	0.26	Subclonal	.	FALSE
MTC07	6	10704992	G	T	PAK1IP1	Missense_Mutation	p.A219S	0.25	0.00	232	70	1	Subclonal	.	FALSE
MTC07	6	11565912	G	C	TMEM170B	Missense_Mutation	p.W37C	0.08	0.00	112	47	0.49	Subclonal	.	FALSE
MTC07	6	13588650	C	A	SIRT5	Missense_Mutation	p.P68Q	0.04	0.00	126	52	0.24	Subclonal	.	FALSE
MTC07	6	33170112	T	G	SLC39A7	Missense_Mutation	p.F236C	0.03	0.00	631	147	0.24	Subclonal	.	FALSE
MTC07	6	34512195	G	C	SPDEF	Missense_Mutation	p.P13R	0.03	0.00	336	93	0.24	Subclonal	.	FALSE
MTC07	6	43568762	A	G	POLH	Missense_Mutation	p.N233S	0.04	0.00	156	43	0.24	Subclonal	.	FALSE
MTC07	6	116720622	G	A	DSE	Missense_Mutation	p.R70H	0.04	0.00	372	156	0.22	Subclonal	LOH	FALSE
MTC07	6	116821709	C	T	TRAPPC3L	Missense_Mutation	p.A121T	0.06	0.00	158	64	0.29	Subclonal	LOH	FALSE
MTC07	6	117010525	G	A	KPNA5	Missense_Mutation	p.S16N	0.04	0.00	231	70	0.2	Subclonal	LOH	FALSE
MTC07	6	148865872	G	A	SASH1	Missense_Mutation	p.R1089Q	0.04	0.00	204	88	0.23	Subclonal	LOH	FALSE
MTC07	7	44432049	G	T	NUDCD3	Missense_Mutation	p.N274K	0.08	0.00	290	134	0.47	Subclonal	.	FALSE
MTC07	7	111927067	A	G	ZNF277	Missense_Mutation	p.K84R	0.28	0.00	392	166	1	Clonal	.	FALSE
MTC07	7	116414971	G	A	MET	Missense_Mutation	p.R1022Q	0.04	0.00	95	29	0.22	Subclonal	.	FALSE
MTC07	8	42229081	G	A	POLB	Missense_Mutation	p.G305E	0.18	0.00	262	75	1	Clonal	.	FALSE
MTC07	8	53571491	A	C	RB1CC1	Missense_Mutation	p.L579V	0.03	0.00	302	72	0.27	Subclonal	.	FALSE
MTC07	8	77762545	C	G	ZFHX4	Nonsense_Mutation	p.S1304*	0.06	0.00	419	96	0.5	Subclonal	.	FALSE
MTC07	8	82606003	G	C	SLC10A5	Missense_Mutation	p.T402R	0.03	0.00	481	102	0.22	Subclonal	.	FALSE
MTC07	8	144511538	C	A	MAFA	Missense_Mutation	p.G347C	0.06	0.00	255	101	0.36	Subclonal	LOH	FALSE
MTC07	8	145154910	G	A	SHARPIN	Missense_Mutation	p.P147S	0.05	0.00	79	19	0.31	Subclonal	LOH	FALSE
MTC07	9	5968256	C	A	KIAA2026	Missense_Mutation	p.V659F	0.03	0.00	443	116	0.21	Subclonal	.	FALSE
MTC07	9	14748573	C	G	FREM1	Missense_Mutation	p.W1874C	0.04	0.00	300	91	0.27	Subclonal	.	FALSE
MTC07	9	14775917	T	A	FREM1	Missense_Mutation	p.N1576I	0.03	0.00	514	163	0.18	Subclonal	.	FALSE
MTC07	9	35906631	G	T	HRCT1	Nonstop_Mutation	p.*116Lext*36	0.06	0.00	66	19	0.43	Subclonal	.	FALSE
MTC07	9	90501980	G	A	SPATA31E1	Missense_Mutation	p.V860I	0.03	0.00	240	70	0.2	Subclonal	.	FALSE
MTC07	9	97869370	A	G	FANCC	Missense_Mutation	p.I504T	0.12	0.00	105	32	0.76	Subclonal	.	FALSE
MTC07	9	113169659	A	G	SVEP1	Missense_Mutation	p.Y2741H	0.03	0.00	430	140	0.22	Subclonal	.	FALSE
MTC07	9	113562952	C	G	MUSK	Missense_Mutation	p.P765R	0.08	0.00	670	236	0.56	Subclonal	.	FALSE
MTC07	9	130858570	C	A	ST6GALNAC6	Missense_Mutation	p.R29L	0.05	0.00	197	49	0.31	Subclonal	.	FALSE
MTC07	9	131243901	C	T	ODF2	Missense_Mutation	p.R296W	0.17	0.00	282	87	1	Clonal	.	FALSE
MTC07	23	39933418	G	A	BCOR	Missense_Mutation	p.P394L	0.03	0.00	375	139	0.18	Subclonal	.	FALSE
MTC07	23	47433975	G	C	SYN1	Missense_Mutation	p.P470A	0.10	0.00	110	36	0.51	Subclonal	.	FALSE
MTC07	23	67937576	A	G	STARD8	Missense_Mutation	p.T274A	0.05	0.00	326	128	0.27	Subclonal	.	FALSE
MTC07	23	101909549	G	A	GPRASP1	Missense_Mutation	p.M236I	0.03	0.00	379	167	0.15	Subclonal	.	FALSE
MTC07	23	102968498	G	A	GLRA4	Nonsense_Mutation	p.Q345*	0.22	0.00	248	103	1	Clonal	.	FALSE
MTC07	23	110459733	C	T	PAK3	Missense_Mutation	p.R534C	0.14	0.00	248	87	0.72	Subclonal	.	FALSE
MTC07	23	114425196	G	A	RBMXL3	Missense_Mutation	p.D398N	0.52	0.00	243	97	1	Clonal	.	FALSE
MTC11	1	16464531	C	T	EPHA2	Missense_Mutation	p.G377R	0.07	0.00	180	73	0.6	Subclonal	.	FALSE
MTC11	1	27105720	AGAAGAG	A	ARID1A	In_Frame_Del	p.E1779_E1780del	0.06	0.00	409	146	0.55	Subclonal	.	FALSE
MTC11	1	62910795	C	T	USP1	Missense_Mutation	p.P315L	0.08	0.01	282	158	0.74	Subclonal	.	FALSE
MTC11	1	154569604	T	C	ADAR	Missense_Mutation	p.N692D	0.08	0.01	361	170	0.7	Subclonal	.	FALSE
MTC11	1	155039453	G	C	EFNA4	Missense_Mutation	p.G121R	0.12	0.00	270	85	1	Clonal	.	FALSE
MTC11	1	179990077	A	T	CEP350	Missense_Mutation	p.E1056D	0.04	0.00	347	111	0.34	Subclonal	.	FALSE
MTC11	1	202407115	G	T	PPP1R12B	Missense_Mutation	p.S474I	0.04	0.01	372	136	0.34	Subclonal	.	FALSE
MTC11	10	21177105	A	T	NEBL	Missense_Mutation	p.L97H	0.07	0.00	172	76	0.63	Subclonal	.	FALSE
MTC11	10	32323684	C	T	KIF5B	Missense_Mutation	p.E349K	0.09	0.00	75	64	0.85	Clonal	.	FALSE
MTC11	10	79589262	T	C	DLG5	Missense_Mutation	p.N679S	0.04	0.00	810	106	0.93	Clonal	.	FALSE
MTC11	10	93768025	A	T	BTAF1	Missense_Mutation	p.Y1269F	0.11	0.00	101	63	0.88	Clonal	LOH	FALSE
MTC11	10	103917017	C	T	NOLC1	Missense_Mutation	p.S83L	0.10	0.01	261	103	0.83	Clonal	LOH	FALSE
MTC11	11	1092362	A	G	MUC2	Missense_Mutation	p.K1394R	0.09	0.01	181	85	0.71	Subclonal	.	FALSE
MTC11	11	64435067	G	A	NRXN2	Missense_Mutation	p.R485C	0.03	0.00	404	129	0.29	Subclonal	.	FALSE
MTC11	11	64893333	A	G	MRPL49	Missense_Mutation	p.K164E	0.09	0.01	350	89	0.78	Subclonal	.	FALSE
MTC11	11	65412478	C	A	SIPA1	Nonsense_Mutation	p.S346*	0.10	0.02	181	66	0.9	Clonal	.	FALSE
MTC11	11	78383281	G	T	TENM4	Missense_Mutation	p.L1864M	0.05	0.00	347	136	0.39	Subclonal	LOH	FALSE
MTC11	11	111608248	C	A	PPP2R1B	Missense_Mutation	p.S612I	0.10	0.00	174	56	0.83	Clonal	LOH	FALSE
MTC11	13	73319149	A	C	BORA	Missense_Mutation	p.D181A	0.13	0.02	190	81	1	Clonal	.	FALSE
MTC11	13	115091481	G	A	CHAMP1	Missense_Mutation	p.G722S	0.14	0.01	319	146	1	Clonal	.	FALSE
MTC11	14	23856195	G	T	MYH7	Missense_Mutation	p.L714I	0.06	0.00	186	78	0.47	Subclonal	.	FALSE
MTC11	14	24109103	A	G	DHRS2	Missense_Mutation	p.K140R	0.09	0.00	215	67	0.75	Subclonal	.	FALSE
MTC11	15	80762590	C	T	ARNT2	Missense_Mutation	p.R78W	0.25	0.02	394	161	1	Clonal	.	FALSE
MTC11	16	2026933	G	T	TBL3	Missense_Mutation	p.A471S	0.11	0.02	305	93	1	Clonal	.	FALSE
MTC11	16	2026934	C	T	TBL3	Missense_Mutation	p.A471V	0.11	0.02	308	94	1	Clonal	.	FALSE
MTC11	16	22926623	G	T	HS3ST2	Missense_Mutation	p.A282S	0.07	0.01	443	160	0.63	Subclonal	.	FALSE
MTC11	16	62055220	C	A	CDH8	Missense_Mutation	p.A30S	0.07	0.01	284	133	0.6	Subclonal	.	FALSE
MTC11	16	88600656	C	T	ZFPM1	Missense_Mutation	p.P764S	0.12	0.00	49	47	0.99	Clonal	.	FALSE
MTC11	17	7121949	G	A	DLG4	Missense_Mutation	p.S10L	0.17	0.01	282	89	1	Clonal	LOH	FALSE
MTC11	17	26712106	C	T	SARM1	Missense_Mutation	p.T447M	0.08	0.01	408	104	0.84	Clonal	.	FALSE
MTC11	17	37006629	T	G	RPL23	Missense_Mutation	p.K109T	0.13	0.01	231	123	1	Clonal	.	FALSE
MTC11	17	39919519	C	A	JUP	Missense_Mutation	p.V405F	0.09	0.00	367	147	0.81	Subclonal	.	FALSE
MTC11	17	57970680	G	T	RPS6KB1	Missense_Mutation	p.E45D	0.21	0.02	202	53	1	Clonal	.	FALSE
MTC11	18	70209148	C	T	CBLN2	Missense_Mutation	p.G83D	0.04	0.00	213	92	0.3	Subclonal	.	FALSE
MTC11	19	2097468	A	T	IZUMO4	Missense_Mutation	p.H115L	0.29	0.01	479	195	1	Clonal	.	FALSE
MTC11	19	8326594	G	C	CERS4	Missense_Mutation	p.E291Q	0.06	0.00	395	126	0.61	Subclonal	.	FALSE
MTC11	19	10404824	A	G	ICAM5	Missense_Mutation	p.K807R	0.03	0.00	584	226	0.23	Subclonal	.	FALSE
MTC11	19	15355045	G	C	BRD4	Missense_Mutation	p.P860A	0.10	0.00	148	55	0.92	Clonal	.	FALSE
MTC11	19	19745959	G	A	GMIP	Missense_Mutation	p.L542F	0.05	0.01	340	116	0.5	Subclonal	.	FALSE
MTC11	19	47151917	C	T	DACT3	Missense_Mutation	p.G571D	0.03	0.00	315	131	0.35	Subclonal	.	FALSE
MTC11	19	50926154	G	A	SPIB	Missense_Mutation	p.A67T	0.09	0.00	118	50	0.94	Clonal	.	FALSE
MTC11	19	50936438	G	T	MYBPC2	Missense_Mutation	p.G14C	0.05	0.00	189	81	0.48	Subclonal	.	FALSE
MTC11	2	55253427	G	A	RTN4	Missense_Mutation	p.S603L	0.16	0.02	286	116	1	Clonal	.	FALSE
MTC11	2	86709180	C	G	KDM3A	Missense_Mutation	p.N880K	0.13	0.01	378	161	1	Clonal	.	FALSE
MTC11	2	149226110	C	T	MBD5	Nonsense_Mutation	p.R200*	0.03	0.01	403	186	0.26	Subclonal	.	FALSE
MTC11	2	203684485	A	T	ICA1L	Missense_Mutation	p.M166K	0.24	0.04	289	125	1	Clonal	.	FALSE
MTC11	2	206058019	G	C	PARD3B	Missense_Mutation	p.L643F	0.15	0.00	67	36	1	Clonal	.	FALSE
MTC11	20	3329179	G	A	C20orf194	Missense_Mutation	p.A261V	0.13	0.00	154	71	1	Clonal	.	FALSE
MTC11	20	47838061	G	A	DDX27	Missense_Mutation	p.V87I	0.19	0.04	150	56	1	Clonal	.	FALSE
MTC11	20	50407283	G	T	SALL4	Missense_Mutation	p.S580Y	0.12	0.00	517	137	1	Clonal	.	FALSE
MTC11	20	54974056	C	T	CSTF1	Missense_Mutation	p.P227S	0.08	0.01	280	156	0.75	Subclonal	.	FALSE
MTC11	20	60963389	C	T	RPS21	Nonsense_Mutation	p.R71*	0.08	0.01	402	118	0.83	Clonal	.	FALSE
MTC11	21	34939479	C	G	SON	Missense_Mutation	p.T2225R	0.10	0.00	90	38	1	Clonal	.	FALSE
MTC11	22	31655180	A	T	LIMK2	Missense_Mutation	p.D88V	0.12	0.00	202	50	0.96	Clonal	.	FALSE
MTC11	22	45822026	G	T	RIBC2	Missense_Mutation	p.V287F	0.09	0.01	365	147	0.97	Clonal	.	FALSE
MTC11	22	46656373	C	T	PKDREJ	Missense_Mutation	p.E283K	0.08	0.00	464	127	0.88	Clonal	.	FALSE
MTC11	3	78717285	T	C	ROBO1	Missense_Mutation	p.S800G	0.04	0.00	276	149	0.33	Subclonal	.	FALSE
MTC11	3	142773896	A	T	U2SURP	Missense_Mutation	p.K962N	0.04	0.00	167	71	0.38	Subclonal	.	FALSE
MTC11	3	183959650	G	C	VWA5B2	Missense_Mutation	p.D1185H	0.08	0.01	447	124	0.71	Subclonal	.	FALSE
MTC11	3	185191203	G	T	MAP3K13	Missense_Mutation	p.S69I	0.08	0.01	345	139	0.76	Subclonal	.	FALSE
MTC11	4	5731089	T	G	EVC	Missense_Mutation	p.V119G	0.07	0.01	122	79	0.53	Subclonal	.	FALSE
MTC11	4	20618656	C	G	SLIT2	Missense_Mutation	p.P1324R	0.06	0.01	198	110	0.49	Subclonal	.	FALSE
MTC11	4	37444947	A	T	KIAA1239	Missense_Mutation	p.D446V	0.18	0.03	393	150	1	Clonal	.	FALSE
MTC11	4	109740473	C	T	COL25A1	Missense_Mutation	p.V620I	0.11	0.00	251	123	0.9	Clonal	.	FALSE
MTC11	4	144620960	A	T	FREM3	Missense_Mutation	p.F290Y	0.08	0.01	353	149	0.62	Subclonal	.	FALSE
MTC11	4	154197128	G	T	TRIM2	Missense_Mutation	p.G100V	0.05	0.01	319	125	0.43	Subclonal	.	FALSE
MTC11	5	40950059	G	A	C7	Missense_Mutation	p.V34I	0.09	0.01	273	73	0.92	Clonal	.	FALSE
MTC11	5	56155555	T	A	MAP3K1	Missense_Mutation	p.I216N	0.13	0.00	115	68	1	Clonal	.	FALSE
MTC11	5	79732722	A	G	ZFYVE16	Missense_Mutation	p.Y73C	0.16	0.01	285	130	1	Clonal	.	FALSE
MTC11	5	121188148	C	G	FTMT	Missense_Mutation	p.L164V	0.10	0.02	317	154	0.79	Subclonal	.	FALSE
MTC11	5	157078735	C	G	SOX30	Missense_Mutation	p.G118R	0.13	0.00	332	117	1	Clonal	.	FALSE
MTC11	5	172550172	C	T	CREBRF	Nonsense_Mutation	p.Q591*	0.06	0.00	224	85	0.53	Subclonal	.	FALSE
MTC11	6	27806527	C	T	HIST1H2BN	Missense_Mutation	p.R30C	0.05	0.00	199	143	0.36	Subclonal	.	FALSE
MTC11	6	31598512	C	A	PRRC2A	Missense_Mutation	p.P800H	0.20	0.00	195	88	1	Clonal	.	FALSE
MTC11	6	35288994	C	A	DEF6	Nonsense_Mutation	p.S568*	0.25	0.00	56	55	1	Clonal	.	FALSE
MTC11	6	70966501	T	A	COL9A1	Missense_Mutation	p.E491D	0.09	0.01	213	89	0.85	Clonal	.	FALSE
MTC11	6	123595817	T	C	TRDN	Splice_Site	p.X523_splice	0.09	0.00	43	19	0.75	Subclonal	.	FALSE
MTC11	7	21470260	C	T	SP4	Nonsense_Mutation	p.Q493*	0.04	0.00	482	200	0.44	Subclonal	.	FALSE
MTC11	7	44147608	C	A	AEBP1	Missense_Mutation	p.P289T	0.32	0.05	157	82	1	Clonal	.	FALSE
MTC11	7	48318202	A	G	ABCA13	Missense_Mutation	p.T2471A	0.07	0.01	403	195	0.67	Subclonal	.	FALSE
MTC11	7	49815384	C	T	VWC2	Missense_Mutation	p.P118L	0.08	0.01	452	116	0.76	Subclonal	.	FALSE
MTC11	7	82595385	T	A	PCLO	Missense_Mutation	p.K1240M	0.04	0.00	331	227	0.36	Subclonal	.	FALSE
MTC11	7	83610743	G	A	SEMA3A	Missense_Mutation	p.R516W	0.19	0.01	194	69	1	Clonal	.	FALSE
MTC11	7	153750014	G	A	DPP6	Missense_Mutation	p.G37S	0.11	0.00	27	15	1	Clonal	.	FALSE
MTC11	7	155255316	C	G	EN2	Missense_Mutation	p.H312Q	0.04	0.00	172	99	0.37	Subclonal	.	FALSE
MTC11	9	18826373	C	G	ADAMTSL1	Missense_Mutation	p.N1342K	0.03	0.00	271	106	0.21	Subclonal	.	FALSE
MTC11	9	135173631	C	A	SETX	Nonsense_Mutation	p.E1873*	0.07	0.01	241	120	0.6	Subclonal	.	FALSE
MTC11	9	139905722	G	C	ABCA2	Missense_Mutation	p.L1947V	0.08	0.00	504	176	0.7	Subclonal	.	FALSE
MTC11	23	107976823	C	G	IRS4	Missense_Mutation	p.S918P	0.05	0.00	318	188	0.38	Subclonal	.	FALSE
MTC11	23	114425196	G	A	RBMXL3	Missense_Mutation	p.D398N	0.53	0.00	213	151	1	Clonal	.	FALSE
MTC12	1	12343234	G	A	VPS13D	Missense_Mutation	p.R1692Q	0.06	0.00	125	86	0.33	Subclonal	.	FALSE
MTC12	1	26691576	T	C	ZNF683	Missense_Mutation	p.N154S	0.18	0.00	105	53	1	Clonal	.	FALSE
MTC12	1	120306981	CCA	C	HMGCS2	Frame_Shift_Del	p.V124Gfs*8	0.23	0.00	145	151	0.93	Clonal	LOH	FALSE
MTC12	1	170521205	A	G	GORAB	Missense_Mutation	p.I263V	0.23	0.00	211	145	1	Clonal	.	FALSE
MTC12	1	201869631	G	A	SHISA4	Missense_Mutation	p.V99M	0.13	0.00	186	106	0.67	Subclonal	.	FALSE
MTC12	10	89692851	T	A	PTEN	Missense_Mutation	p.L112Q	0.33	0.00	119	134	1	Clonal	LOH	FALSE
MTC12	10	134682812	C	G	TTC40	Missense_Mutation	p.G1526R	0.07	0.00	61	28	0.33	Subclonal	.	FALSE
MTC12	11	6341427	C	T	PRKCDBP	Missense_Mutation	p.A94T	0.22	0.00	91	49	1	Clonal	.	FALSE
MTC12	11	47609658	C	A	FAM180B	Missense_Mutation	p.S116Y	0.05	0.00	128	79	0.27	Subclonal	.	FALSE
MTC12	11	63721932	G	C	NAA40	Missense_Mutation	p.C232S	0.07	0.00	196	133	0.35	Subclonal	.	FALSE
MTC12	11	69950167	C	G	ANO1	Missense_Mutation	p.I201M	0.16	0.00	217	132	0.78	Subclonal	.	FALSE
MTC12	11	92526009	G	T	FAT3	Missense_Mutation	p.S1563I	0.18	0.00	195	162	0.92	Clonal	.	FALSE
MTC12	11	100064250	G	C	CNTN5	Missense_Mutation	p.R580T	0.16	0.00	74	99	0.8	Subclonal	.	FALSE
MTC12	11	123676886	A	G	OR6M1	Missense_Mutation	p.Y58H	0.17	0.01	174	120	0.83	Subclonal	.	FALSE
MTC12	12	306018	T	A	SLC6A12	Missense_Mutation	p.K369M	0.07	0.00	96	49	0.36	Subclonal	.	FALSE
MTC12	13	70514320	G	A	KLHL1	Missense_Mutation	p.A289V	0.38	0.00	34	26	1	Clonal	LOH	FALSE
MTC12	14	81743704	C	T	STON2	Missense_Mutation	p.V651M	0.40	0.00	310	138	1	Clonal	.	FALSE
MTC12	15	29385283	G	T	APBA2	Missense_Mutation	p.G359W	0.05	0.00	133	126	0.18	Subclonal	LOH	FALSE
MTC12	15	78293995	G	A	TBC1D2B	Missense_Mutation	p.L888P	0.16	0.00	129	69	0.97	Clonal	.	FALSE
MTC12	15	80873947	C	T	ARNT2	Missense_Mutation	p.P607L	0.21	0.00	214	140	1	Clonal	.	FALSE
MTC12	16	24816116	C	G	TNRC6A	Missense_Mutation	p.Q1310E	0.11	0.00	91	66	0.55	Subclonal	.	FALSE
MTC12	16	30991306	G	A	SETD1A	Missense_Mutation	p.R1400H	0.25	0.00	51	17	1	Clonal	.	FALSE
MTC12	16	53671766	C	T	RPGRIP1L	Missense_Mutation	p.V1021I	0.12	0.00	90	68	0.61	Subclonal	.	FALSE
MTC12	17	7577022	G	A	TP53	Nonsense_Mutation	p.R306*	0.35	0.00	191	137	1	Clonal	LOH	FALSE
MTC12	17	7758968	C	T	TMEM88	Missense_Mutation	p.P139L	0.32	0.00	145	119	1	Clonal	LOH	FALSE
MTC12	17	64125931	G	A	CEP112	Missense_Mutation	p.S192L	0.29	0.00	85	34	1	Clonal	.	FALSE
MTC12	19	15302346	C	T	NOTCH3	Missense_Mutation	p.E309K	0.15	0.00	220	100	0.59	Subclonal	LOH	FALSE
MTC12	19	15302399	C	T	NOTCH3	Missense_Mutation	p.C291Y	0.12	0.00	201	112	0.47	Subclonal	LOH	FALSE
MTC12	19	15302405	C	T	NOTCH3	Missense_Mutation	p.G289D	0.11	0.00	198	107	0.42	Subclonal	LOH	FALSE
MTC12	19	15302445	C	T	NOTCH3	Missense_Mutation	p.D276N	0.09	0.00	176	103	0.34	Subclonal	LOH	FALSE
MTC12	19	15587227	G	A	PGLYRP2	Missense_Mutation	p.P85L	0.24	0.00	242	136	0.95	Clonal	LOH	FALSE
MTC12	19	22836805	G	A	ZNF492	Missense_Mutation	p.A40T	0.10	0.00	77	57	0.41	Subclonal	LOH	FALSE
MTC12	19	35435294	G	A	ZNF30	Missense_Mutation	p.R476Q	0.14	0.00	139	95	0.68	Subclonal	.	FALSE
MTC12	19	38136525	T	C	ZFP30	Missense_Mutation	p.N41S	0.20	0.00	79	45	1	Clonal	.	FALSE
MTC12	19	38934223	C	T	RYR1	Missense_Mutation	p.T99M	0.15	0.00	98	56	0.76	Subclonal	.	FALSE
MTC12	19	52869083	T	G	ZNF610	Missense_Mutation	p.V151G	0.15	0.00	172	116	0.87	Clonal	.	FALSE
MTC12	2	135888220	A	T	RAB3GAP1	Nonsense_Mutation	p.K389*	0.19	0.00	164	113	0.94	Clonal	.	FALSE
MTC12	2	196544959	G	T	SLC39A10	Nonsense_Mutation	p.E65*	0.10	0.00	199	129	0.6	Subclonal	.	FALSE
MTC12	2	201534325	G	T	AOX1	Missense_Mutation	p.G1276W	0.23	0.00	61	50	1	Clonal	.	FALSE
MTC12	20	30584914	C	A	XKR7	Missense_Mutation	p.P465H	0.10	0.00	220	125	0.52	Subclonal	.	FALSE
MTC12	22	29747188	G	T	AP1B1	Missense_Mutation	p.A376D	0.05	0.00	128	75	0.27	Subclonal	.	FALSE
MTC12	3	14861906	G	A	FGD5	Missense_Mutation	p.G443E	0.04	0.00	210	153	0.17	Subclonal	LOH	FALSE
MTC12	3	38950540	G	T	PLCD1	Nonsense_Mutation	p.C593*	0.21	0.00	24	24	0.83	Clonal	LOH	FALSE
MTC12	3	141162996	C	T	ZBTB38	Missense_Mutation	p.A589V	0.03	0.00	288	164	0.19	Subclonal	.	FALSE
MTC12	3	153973167	C	A	ARHGEF26	Missense_Mutation	p.P841T	0.18	0.00	125	70	0.87	Clonal	.	FALSE
MTC12	4	7698990	A	C	SORCS2	Missense_Mutation	p.T553P	0.27	0.00	70	56	1	Clonal	.	FALSE
MTC12	4	153896887	T	C	FHDC1	Missense_Mutation	p.M815T	0.13	0.00	273	155	0.65	Subclonal	.	FALSE
MTC12	5	67576760	A	T	PIK3R1	Missense_Mutation	p.D281V	0.19	0.00	70	76	0.92	Clonal	.	FALSE
MTC12	5	72874855	T	A	UTP15	Missense_Mutation	p.I387K	0.17	0.00	123	84	0.85	Clonal	.	FALSE
MTC12	6	42196386	G	T	TRERF1	Missense_Mutation	p.S1100R	0.03	0.00	147	98	0.17	Subclonal	.	FALSE
MTC12	7	33055337	G	A	NT5C3A	Missense_Mutation	p.A285V	0.17	0.00	47	23	1	Clonal	.	FALSE
MTC12	7	83631288	C	T	SEMA3A	Missense_Mutation	p.E479K	0.20	0.00	188	168	1	Clonal	.	FALSE
MTC12	7	107745012	C	T	LAMB4	Missense_Mutation	p.R308K	0.23	0.00	194	131	1	Clonal	.	FALSE
MTC12	8	23179750	C	A	LOXL2	Nonsense_Mutation	p.E399*	0.04	0.00	93	92	0.17	Subclonal	LOH	FALSE
MTC12	8	41584805	A	G	ANK1	Missense_Mutation	p.L163S	0.22	0.00	77	88	1	Clonal	.	FALSE
MTC12	8	105509494	C	T	LRP12	Missense_Mutation	p.R429H	0.21	0.00	310	238	1	Clonal	.	FALSE
MTC12	9	7103841	T	C	KDM4C	Missense_Mutation	p.C861R	0.15	0.00	62	53	0.72	Subclonal	.	FALSE
MTC12	23	47486983	T	C	CFP	Missense_Mutation	p.K154R	0.11	0.01	139	77	0.54	Subclonal	.	FALSE
MTC12	23	70367889	G	T	NLGN3	Missense_Mutation	p.R97L	0.18	0.00	207	137	0.89	Clonal	.	FALSE
MTC12	23	70524080	T	C	ITGB1BP2	Missense_Mutation	p.L228S	0.04	0.00	149	106	0.2	Subclonal	.	FALSE
MTC12	23	132351090	C	G	TFDP3	Missense_Mutation	p.E400Q	0.03	0.00	240	129	0.17	Subclonal	.	FALSE
MTC12	23	137715055	C	T	FGF13	Missense_Mutation	p.V242M	0.11	0.00	108	44	0.66	Subclonal	.	FALSE
MTC13	1	16556010	G	A	C1orf134	Missense_Mutation	p.A10V	0.20	0.00	142	68	0.62	Subclonal	.	FALSE
MTC13	1	110257864	C	T	GSTM5	Missense_Mutation	p.R187C	0.03	0.00	243	141	0.1	Subclonal	.	FALSE
MTC13	1	237870512	A	C	RYR2	Missense_Mutation	p.K3282Q	0.07	0.00	605	385	0.37	Subclonal	.	FALSE
MTC13	11	802297	G	A	PIDD	Missense_Mutation	p.P372S	0.04	0.00	136	76	0.14	Subclonal	.	FALSE
MTC13	11	4615316	G	A	OR52I1	Missense_Mutation	p.F16L	0.35	0.00	288	202	1	Clonal	.	FALSE
MTC13	12	49446463	C	T	KMT2D	Missense_Mutation	p.R83Q	0.10	0.00	417	159	0.4	Subclonal	.	FALSE
MTC13	12	104064552	G	A	STAB2	Missense_Mutation	p.D780N	0.12	0.00	311	103	0.5	Subclonal	.	FALSE
MTC13	13	32783169	G	A	FRY	Missense_Mutation	p.G1400R	0.04	0.00	250	86	0.11	Subclonal	.	FALSE
MTC13	13	43462422	A	T	EPSTI1	Missense_Mutation	p.N399K	0.04	0.00	298	136	0.12	Subclonal	.	FALSE
MTC13	14	73717646	C	T	PAPLN	Missense_Mutation	p.S166F	0.04	0.00	191	125	0.11	Subclonal	.	FALSE
MTC13	14	77274474	A	C	ANGEL1	Missense_Mutation	p.W223G	0.04	0.00	130	70	0.12	Subclonal	.	FALSE
MTC13	14	107078483	G	T	IGHV1-58	Missense_Mutation	p.Q81K	0.25	0.00	212	119	0.77	Subclonal	.	FALSE
MTC13	16	789680	C	T	NARFL	Missense_Mutation	p.R42H	0.27	0.00	273	109	0.84	Subclonal	.	FALSE
MTC13	16	27268779	G	C	NSMCE1	Missense_Mutation	p.T38R	0.07	0.00	97	48	0.3	Subclonal	.	FALSE
MTC13	17	42837203	G	A	ADAM11	Missense_Mutation	p.G59R	0.11	0.00	212	61	0.45	Subclonal	.	FALSE
MTC13	19	16640630	G	A	CHERP	Nonsense_Mutation	p.Q320*	0.11	0.00	198	87	0.33	Subclonal	.	FALSE
MTC13	19	51380133	C	T	KLK2	Missense_Mutation	p.R167C	0.09	0.00	360	150	0.28	Subclonal	.	FALSE
MTC13	19	56450946	G	A	NLRP7	Missense_Mutation	p.T414M	0.18	0.00	342	170	0.56	Subclonal	.	FALSE
MTC13	2	212578291	C	A	ERBB4	Missense_Mutation	p.M322I	0.04	0.00	228	71	0.14	Subclonal	.	FALSE
MTC13	20	40877462	C	G	PTPRT	Missense_Mutation	p.G745A	0.08	0.00	170	55	0.34	Subclonal	.	FALSE
MTC13	22	26888831	G	T	SEZ6L	Missense_Mutation	p.W165L	0.03	0.00	283	104	0.1	Subclonal	.	FALSE
MTC13	3	184091304	G	A	THPO	Missense_Mutation	p.R99W	0.07	0.00	188	84	0.22	Subclonal	.	FALSE
MTC13	4	144354763	C	T	GAB1	Nonsense_Mutation	p.Q163*	0.12	0.00	234	114	0.39	Subclonal	.	FALSE
MTC13	5	64865743	TTGTTC	T	PPWD1	Frame_Shift_Del	p.S150Gfs*2	0.34	0.00	175	42	1	Clonal	.	FALSE
MTC13	5	137284676	G	A	FAM13B	Missense_Mutation	p.R888C	0.30	0.00	164	87	0.93	Subclonal	.	FALSE
MTC13	5	149429927	C	T	HMGXB3	Missense_Mutation	p.S1105L	0.03	0.00	240	96	0.1	Subclonal	.	FALSE
MTC13	7	37934114	G	T	NME8	Missense_Mutation	p.Q482H	0.05	0.00	249	101	0.2	Subclonal	.	FALSE
MTC13	7	66459197	A	G	SBDS	Splice_Site	p.X86_splice	0.62	0.00	85	20	1	Clonal	.	FALSE
MTC13	7	142013441	C	T	TRBV4-1	Missense_Mutation	p.A99V	0.14	0.00	347	161	0.57	Subclonal	.	FALSE
MTC13	8	20038388	C	T	SLC18A1	Missense_Mutation	p.A30T	0.13	0.00	120	74	0.39	Subclonal	.	FALSE
MTC13	8	92082559	C	G	OTUD6B	Missense_Mutation	p.P13A	0.14	0.00	242	89	0.44	Subclonal	.	FALSE
MTC13	23	3228382	G	A	MXRA5	Missense_Mutation	p.A2621V	0.07	0.00	57	27	0.15	Subclonal	LOH	FALSE
MTC14	1	2235512	CGGAGGT	C	SKI	In_Frame_Del	p.V486_E487del	0.23	0.00	189	93	1	Clonal	LOH	FALSE
MTC14	1	8420805	G	A	RERE	Missense_Mutation	p.A921V	0.08	0.00	152	54	0.36	Subclonal	LOH	FALSE
MTC14	1	43909427	T	C	SZT2	Missense_Mutation	p.F2872L	0.07	0.00	76	27	0.3	Subclonal	.	FALSE
MTC14	1	75171990	A	T	CRYZ	Missense_Mutation	p.L327H	0.21	0.01	200	109	0.92	Clonal	.	FALSE
MTC14	1	109191501	C	T	HENMT1	Missense_Mutation	p.R290Q	0.07	0.01	226	94	0.3	Subclonal	.	FALSE
MTC14	1	110459920	T	G	CSF1	Missense_Mutation	p.D77E	0.05	0.00	279	122	0.24	Subclonal	.	FALSE
MTC14	1	160093775	G	C	ATP1A2	Missense_Mutation	p.G142R	0.15	0.00	328	92	0.97	Clonal	.	FALSE
MTC14	1	161953517	T	C	OLFML2B	Missense_Mutation	p.Y734C	0.03	0.00	551	137	0.21	Subclonal	.	FALSE
MTC14	1	212534102	G	A	PPP2R5A	Missense_Mutation	p.S484N	0.50	0.00	104	69	1	Clonal	.	FALSE
MTC14	1	227947089	G	C	SNAP47	Missense_Mutation	p.Q342H	0.58	0.01	513	177	1	Clonal	.	FALSE
MTC14	10	12206397	G	C	SEC61A2	Missense_Mutation	p.E459Q	0.03	0.00	345	92	0.26	Subclonal	.	FALSE
MTC14	10	71003060	G	T	HKDC1	Missense_Mutation	p.D272Y	0.26	0.00	161	55	1	Clonal	LOH	FALSE
MTC14	10	75290057	C	G	USP54	Missense_Mutation	p.E558Q	0.10	0.00	517	199	0.54	Subclonal	LOH	FALSE
MTC14	10	76994937	C	T	COMTD1	Splice_Site	p.X110_splice	0.13	0.00	231	86	0.71	Subclonal	LOH	FALSE
MTC14	10	89720653	C	A	PTEN	Missense_Mutation	p.D268E	0.19	0.00	47	13	1	Clonal	LOH	FALSE
MTC14	11	298626	A	G	IFITM5	Missense_Mutation	p.W92R	0.10	0.00	344	159	0.37	Subclonal	.	FALSE
MTC14	11	4112746	G	C	STIM1	Missense_Mutation	p.K592N	0.38	0.01	220	120	1	Clonal	.	FALSE
MTC14	11	4112747	G	T	STIM1	Missense_Mutation	p.A593S	0.37	0.01	217	118	1	Clonal	.	FALSE
MTC14	11	35454193	A	T	PAMR1	Missense_Mutation	p.V642E	0.45	0.00	231	92	1	Clonal	.	FALSE
MTC14	11	66133608	G	A	SLC29A2	Missense_Mutation	p.P321L	0.22	0.00	433	163	1	Clonal	.	FALSE
MTC14	11	73789450	C	T	C2CD3	Missense_Mutation	p.R1438H	0.47	0.01	477	183	1	Clonal	.	FALSE
MTC14	11	92600252	C	T	FAT3	Missense_Mutation	p.R4002C	0.52	0.00	250	68	1	Clonal	.	FALSE
MTC14	12	308097	C	A	SLC6A12	Missense_Mutation	p.V238L	0.66	0.03	123	40	1	Clonal	.	FALSE
MTC14	12	30790026	A	G	IPO8	Missense_Mutation	p.V862A	0.06	0.00	516	161	0.31	Subclonal	.	FALSE
MTC14	12	51089718	G	A	DIP2B	Missense_Mutation	p.R634Q	0.26	0.01	386	154	1	Clonal	.	FALSE
MTC14	12	56031591	G	T	OR10P1	Missense_Mutation	p.V306L	0.08	0.00	330	141	0.38	Subclonal	.	FALSE
MTC14	12	70088246	T	A	BEST3	Splice_Site	p.X51_splice	0.05	0.00	261	77	0.27	Subclonal	.	FALSE
MTC14	12	79689874	C	G	SYT1	Missense_Mutation	p.A167G	0.37	0.01	180	83	1	Clonal	.	FALSE
MTC14	12	122517159	A	C	MLXIP	Missense_Mutation	p.T134P	0.05	0.00	238	125	0.16	Subclonal	LOH	FALSE
MTC14	12	132510280	G	A	EP400	Missense_Mutation	p.A1649T	0.23	0.02	99	50	0.81	Subclonal	LOH	FALSE
MTC14	13	23905655	T	A	SACS	Missense_Mutation	p.L4120F	0.49	0.01	388	173	1	Clonal	.	FALSE
MTC14	13	25488850	C	T	CENPJ	Missense_Mutation	p.G105E	0.05	0.00	352	177	0.2	Subclonal	.	FALSE
MTC14	13	31232261	G	T	USPL1	Missense_Mutation	p.D683Y	0.19	0.00	243	112	0.85	Subclonal	.	FALSE
MTC14	13	79979844	C	A	RBM26	Missense_Mutation	p.E22D	0.54	0.01	378	154	1	Clonal	.	FALSE
MTC14	13	95364242	G	A	SOX21	Missense_Mutation	p.A21V	0.09	0.00	495	252	0.39	Subclonal	.	FALSE
MTC14	13	95364259	G	T	SOX21	Missense_Mutation	p.F15L	0.09	0.00	502	234	0.41	Subclonal	.	FALSE
MTC14	13	100338491	T	C	METTL21C	Missense_Mutation	p.T229A	0.12	0.00	391	169	0.52	Subclonal	.	FALSE
MTC14	13	110830210	G	C	COL4A1	Missense_Mutation	p.P899A	0.07	0.00	153	73	0.29	Subclonal	.	FALSE
MTC14	14	21489973	C	CA	NDRG2	Frame_Shift_Ins	p.R254Sfs*15	0.11	0.00	259	137	0.38	Subclonal	.	FALSE
MTC14	14	60212836	G	C	RTN1	Missense_Mutation	p.P202R	0.22	0.00	430	167	1	Clonal	.	FALSE
MTC14	15	34040347	G	C	RYR3	Missense_Mutation	p.E2674D	0.38	0.01	177	71	1	Clonal	.	FALSE
MTC14	15	62966077	G	C	TLN2	Missense_Mutation	p.K280N	0.05	0.00	217	91	0.21	Subclonal	LOH	FALSE
MTC14	15	66601127	A	T	DIS3L	Missense_Mutation	p.E168V	0.06	0.00	201	95	0.27	Subclonal	LOH	FALSE
MTC14	15	67524215	C	T	AAGAB	Missense_Mutation	p.G158R	0.05	0.00	457	202	0.25	Subclonal	LOH	FALSE
MTC14	15	82431091	G	C	EFTUD1	Missense_Mutation	p.L1028V	0.75	0.00	341	159	1	Clonal	LOH	FALSE
MTC14	16	1270321	G	A	CACNA1H	Missense_Mutation	p.R2130Q	0.56	0.01	439	145	1	Clonal	.	FALSE
MTC14	16	25256157	C	G	ZKSCAN2	Missense_Mutation	p.V644L	0.11	0.00	247	100	0.47	Subclonal	.	FALSE
MTC14	16	57921977	G	A	CNGB1	Missense_Mutation	p.R1082C	0.17	0.01	229	73	0.94	Clonal	.	FALSE
MTC14	16	70285577	T	A	EXOSC6	Missense_Mutation	p.E76V	0.05	0.00	110	22	0.3	Subclonal	.	FALSE
MTC14	16	88805144	C	G	PIEZO1	Missense_Mutation	p.D156H	0.14	0.00	147	77	0.48	Subclonal	LOH	FALSE
MTC14	16	89017676	G	A	RP11-830F9.6	Missense_Mutation	p.V384I	0.23	0.00	166	100	0.8	Subclonal	LOH	FALSE
MTC14	17	7578265	A	G	TP53	Missense_Mutation	p.I195T	0.79	0.00	233	104	1	Clonal	LOH	TRUE
MTC14	17	10348604	C	G	MYH4	Missense_Mutation	p.V1749L	0.06	0.00	157	66	0.26	Subclonal	LOH	FALSE
MTC14	17	21188249	C	G	MAP2K3	Missense_Mutation	p.S6W	0.17	0.00	170	102	0.6	Subclonal	LOH	FALSE
MTC14	17	59853838	T	C	BRIP1	Missense_Mutation	p.D674G	0.06	0.00	386	130	0.39	Subclonal	LOH	FALSE
MTC14	17	73235566	G	A	GGA3	Missense_Mutation	p.P557L	0.07	0.00	231	70	0.45	Subclonal	LOH	FALSE
MTC14	17	76045268	C	G	TNRC6C	Missense_Mutation	p.A42G	0.14	0.00	481	153	0.93	Clonal	LOH	FALSE
MTC14	17	76201752	G	A	AFMID	Missense_Mutation	p.R238H	0.07	0.00	533	169	0.43	Subclonal	LOH	FALSE
MTC14	17	79429851	C	T	RP11-1055B8.7	Missense_Mutation	p.A2490V	0.03	0.00	442	120	0.22	Subclonal	LOH	FALSE
MTC14	18	7050935	C	T	LAMA1	Missense_Mutation	p.V116I	0.05	0.00	237	130	0.18	Subclonal	LOH	FALSE
MTC14	18	29410926	G	A	TRAPPC8	Missense_Mutation	p.S1367L	0.73	0.01	227	137	1	Clonal	LOH	FALSE
MTC14	19	1241889	C	T	ATP5D	Missense_Mutation	p.R14C	0.73	0.00	253	142	1	Clonal	LOH	FALSE
MTC14	19	16631020	CGGGACCGCGACT	C	CHERP	In_Frame_Del	p.Q797_S800del	0.05	0.00	167	99	0.19	Subclonal	LOH	FALSE
MTC14	19	47422960	C	G	ARHGAP35	Missense_Mutation	p.A343G	0.22	0.01	546	182	1	Clonal	LOH	FALSE
MTC14	19	55496535	G	T	NLRP2	Missense_Mutation	p.R717S	0.21	0.00	149	97	0.75	Subclonal	LOH	FALSE
MTC14	19	55496536	A	G	NLRP2	Missense_Mutation	p.I718V	0.21	0.00	147	96	0.74	Subclonal	LOH	FALSE
MTC14	2	8934075	G	A	KIDINS220	Missense_Mutation	p.R381W	0.04	0.00	372	100	0.32	Subclonal	.	FALSE
MTC14	2	29256416	C	A	FAM179A	Missense_Mutation	p.P738T	0.17	0.00	120	35	0.92	Clonal	LOH	FALSE
MTC14	2	32692599	G	A	BIRC6	Missense_Mutation	p.R1788K	0.19	0.00	289	105	0.85	Subclonal	.	FALSE
MTC14	2	49190402	T	A	FSHR	Missense_Mutation	p.M520L	0.49	0.01	322	150	1	Clonal	.	FALSE
MTC14	2	70068620	C	G	GMCL1	Missense_Mutation	p.S139C	0.10	0.00	196	90	0.34	Subclonal	.	FALSE
MTC14	2	80530488	C	G	LRRTM1	Missense_Mutation	p.A153P	0.12	0.00	375	148	0.53	Subclonal	.	FALSE
MTC14	2	85818882	C	A	VAMP5	Missense_Mutation	p.A13E	0.27	0.01	414	181	1	Clonal	.	FALSE
MTC14	2	135743789	G	A	MAP3K19	Nonsense_Mutation	p.R885*	0.12	0.00	289	95	0.56	Subclonal	LOH	FALSE
MTC14	2	211471635	G	A	CPS1	Missense_Mutation	p.R727Q	0.07	0.00	148	58	0.3	Subclonal	LOH	FALSE
MTC14	2	234178670	A	C	ATG16L1	Missense_Mutation	p.K222Q	0.12	0.00	58	43	0.54	Subclonal	LOH	FALSE
MTC14	20	13260443	G	A	ISM1	Missense_Mutation	p.D181N	0.07	0.01	309	133	0.34	Subclonal	.	FALSE
MTC14	20	23065564	C	T	CD93	Missense_Mutation	p.V41I	0.26	0.00	476	179	1	Clonal	.	FALSE
MTC14	20	44659911	C	A	ZNF335	Missense_Mutation	p.G47W	0.26	0.00	234	100	1	Clonal	.	FALSE
MTC14	20	55100859	G	T	FAM209A	Splice_Site	p.X84_splice	0.04	0.00	350	113	0.22	Subclonal	.	FALSE
MTC14	21	30329657	C	T	LTN1	Missense_Mutation	p.M1009I	0.07	0.01	213	96	0.32	Subclonal	.	FALSE
MTC14	22	23500218	T	G	RAB36	Missense_Mutation	p.L211R	0.04	0.00	437	200	0.18	Subclonal	.	FALSE
MTC14	22	24808863	C	G	SPECC1L	Missense_Mutation	p.I1084M	0.09	0.00	137	70	0.43	Subclonal	.	FALSE
MTC14	22	50276904	G	C	ZBED4	Missense_Mutation	p.E532Q	0.05	0.00	395	168	0.23	Subclonal	.	FALSE
MTC14	22	50688571	G	C	HDAC10	Missense_Mutation	p.R104G	0.54	0.01	245	110	1	Clonal	.	FALSE
MTC14	3	48725752	C	G	IP6K2	Missense_Mutation	p.S412T	0.06	0.00	375	170	0.26	Subclonal	LOH	FALSE
MTC14	3	49720306	C	A	APEH	Missense_Mutation	p.S638R	0.43	0.00	320	140	1	Clonal	LOH	FALSE
MTC14	3	138153782	C	A	ESYT3	Missense_Mutation	p.P48T	0.06	0.00	325	85	0.46	Subclonal	.	FALSE
MTC14	3	172025182	G	T	FNDC3B	Missense_Mutation	p.G364V	0.06	0.00	548	152	0.34	Subclonal	.	FALSE
MTC14	4	5624575	C	A	EVC2	Missense_Mutation	p.Q730H	0.07	0.00	364	145	0.32	Subclonal	.	FALSE
MTC14	4	20530623	C	G	SLIT2	Missense_Mutation	p.A505G	0.03	0.00	181	78	0.18	Subclonal	.	FALSE
MTC14	4	23815980	G	C	PPARGC1A	Missense_Mutation	p.P378A	0.16	0.00	267	107	0.91	Clonal	.	FALSE
MTC14	4	70709918	C	T	SULT1E1	Missense_Mutation	p.E245K	0.19	0.00	115	73	0.67	Subclonal	LOH	FALSE
MTC14	4	83822020	G	A	THAP9	Missense_Mutation	p.G22D	0.12	0.00	129	49	0.52	Subclonal	LOH	FALSE
MTC14	4	186064616	G	C	SLC25A4	Missense_Mutation	p.E30D	0.33	0.00	190	43	1	Clonal	.	FALSE
MTC14	5	82499522	G	A	XRCC4	Missense_Mutation	p.E212K	0.68	0.01	201	127	1	Clonal	LOH	FALSE
MTC14	6	32095948	G	A	ATF6B	Missense_Mutation	p.P13S	0.11	0.00	180	75	0.79	Subclonal	.	FALSE
MTC14	6	34614486	C	T	C6orf106	Missense_Mutation	p.E135K	0.06	0.00	393	75	0.42	Subclonal	.	FALSE
MTC14	6	70886442	A	T	COL19A1	Missense_Mutation	p.I885L	0.04	0.00	191	101	0.15	Subclonal	LOH	FALSE
MTC14	6	71289195	T	G	C6orf57	Missense_Mutation	p.L48R	0.72	0.00	178	110	1	Clonal	LOH	FALSE
MTC14	6	160560832	C	G	SLC22A1	Missense_Mutation	p.I403M	0.62	0.00	73	72	1	Clonal	LOH	FALSE
MTC14	7	21745156	G	T	DNAH11	Splice_Site	p.X2189_splice	0.08	0.00	364	151	0.36	Subclonal	.	FALSE
MTC14	7	101845092	G	A	CUX1	Missense_Mutation	p.E850K	0.07	0.00	221	108	0.31	Subclonal	.	FALSE
MTC14	7	107557319	C	G	DLD	Missense_Mutation	p.T319S	0.50	0.00	206	86	1	Clonal	.	FALSE
MTC14	7	128494201	G	T	FLNC	Missense_Mutation	p.A2226S	0.06	0.00	229	96	0.26	Subclonal	.	FALSE
MTC14	7	151970951	C	T	KMT2C	Missense_Mutation	p.R284Q	0.05	0.00	393	230	0.22	Subclonal	.	FALSE
MTC14	8	22107646	G	C	POLR3D	Missense_Mutation	p.G327A	0.64	0.02	145	117	1	Clonal	LOH	FALSE
MTC14	8	53580748	C	A	RB1CC1	Missense_Mutation	p.D336Y	0.04	0.00	227	53	0.29	Subclonal	.	FALSE
MTC14	8	56052723	A	T	RP11-386G21.1	Missense_Mutation	p.S20R	0.08	0.00	278	75	0.49	Subclonal	.	FALSE
MTC14	8	77765499	G	T	ZFHX4	Missense_Mutation	p.Q2114H	0.03	0.00	895	178	0.28	Subclonal	.	FALSE
MTC14	8	81897541	G	C	PAG1	Missense_Mutation	p.L116V	0.05	0.00	949	149	0.61	Subclonal	.	FALSE
MTC14	8	82667648	C	T	CHMP4C	Nonsense_Mutation	p.Q138*	0.22	0.00	930	217	1	Clonal	.	FALSE
MTC14	8	95674533	G	C	ESRP1	Missense_Mutation	p.R175P	0.28	0.00	483	88	1	Clonal	.	FALSE
MTC14	9	33344172	A	C	NFX1	Missense_Mutation	p.E777A	0.16	0.00	154	52	0.89	Clonal	.	FALSE
MTC14	9	107555511	C	G	ABCA1	Missense_Mutation	p.E1859D	0.13	0.00	231	151	0.47	Subclonal	LOH	FALSE
MTC14	9	123210215	C	G	CDK5RAP2	Missense_Mutation	p.V995L	0.16	0.00	334	138	0.7	Subclonal	LOH	FALSE
MTC14	9	125330008	G	A	OR1L8	Missense_Mutation	p.T250M	0.10	0.00	268	99	0.44	Subclonal	LOH	FALSE
MTC14	9	139390648	CAG	C	NOTCH1	Frame_Shift_Del	p.P2514Rfs*4	0.79	0.02	144	63	1	Clonal	LOH	FALSE
MTC14	23	27765760	C	A	DCAF8L2	Missense_Mutation	p.L250I	0.05	0.00	610	261	0.21	Subclonal	.	FALSE
MTC14	23	54334491	G	A	WNK3	Missense_Mutation	p.P318L	0.07	0.00	264	81	0.38	Subclonal	.	FALSE
MTC14	23	74280164	G	C	ABCB7	Missense_Mutation	p.T647S	0.43	0.01	279	103	1	Clonal	.	FALSE
MTC14	23	107422008	C	G	COL4A6	Missense_Mutation	p.Q808H	0.44	0.03	279	97	1	Clonal	.	FALSE
MTC14	23	151997815	T	G	CETN2	Missense_Mutation	p.M67L	0.12	0.00	214	71	0.64	Subclonal	.	FALSE
MTC14	23	152612600	G	C	ZNF275	Missense_Mutation	p.A153P	0.38	0.00	605	194	1	Clonal	.	FALSE
MTC14	23	153760892	G	A	G6PD	Missense_Mutation	p.R423C	0.05	0.00	635	240	0.25	Subclonal	.	FALSE
MTC16	1	981416	C	T	AGRN	Missense_Mutation	p.S918L	0.13	0.01	287	176	0.68	Subclonal	LOH	FALSE
MTC16	1	21936630	C	T	RAP1GAP	Missense_Mutation	p.D392N	0.10	0.01	350	168	0.54	Subclonal	LOH	FALSE
MTC16	10	89685316	T	C	PTEN	Splice_Site	p.X70_splice	0.13	0.00	77	57	0.83	Clonal	.	FALSE
MTC16	12	30869444	G	T	CAPRIN2	Missense_Mutation	p.T772N	0.23	0.02	188	119	1	Clonal	LOH	FALSE
MTC16	12	110765433	C	G	ATP2A2	Missense_Mutation	p.R236G	0.25	0.02	224	150	1	Clonal	LOH	FALSE
MTC16	15	40648331	G	C	PHGR1	Missense_Mutation	p.G26R	0.10	0.00	61	31	0.63	Subclonal	.	FALSE
MTC16	16	74537517	C	T	GLG1	Missense_Mutation	p.S225N	0.18	0.01	237	107	1	Clonal	.	FALSE
MTC16	17	7577117	A	G	TP53	Missense_Mutation	p.V274A	0.27	0.01	170	109	1	Clonal	LOH	TRUE
MTC16	19	812599	T	C	LPPR3	Missense_Mutation	p.K738E	0.20	0.00	59	21	1	Clonal	.	FALSE
MTC16	19	17837881	G	A	MAP1S	Missense_Mutation	p.R563H	0.18	0.02	331	142	1	Clonal	.	FALSE
MTC16	19	19576179	C	T	GATAD2A	Missense_Mutation	p.R9W	0.10	0.01	408	255	0.66	Subclonal	.	FALSE
MTC16	3	88190115	C	T	ZNF654	Missense_Mutation	p.T552I	0.19	0.01	199	114	1	Clonal	LOH	FALSE
MTC16	3	178927980	T	C	PIK3CA	Missense_Mutation	p.C420R	0.28	0.03	133	102	1	Clonal	LOH	TRUE
MTC16	5	140475066	G	A	PCDHB2	Missense_Mutation	p.R231Q	0.10	0.00	360	192	0.66	Subclonal	.	FALSE
MTC16	6	52268703	G	C	PAQR8	Missense_Mutation	p.R231P	0.20	0.03	265	142	1	Clonal	LOH	FALSE
MTC16	7	6631413	G	A	C7orf26	Missense_Mutation	p.R110Q	0.03	0.01	445	159	0.23	Subclonal	.	FALSE
MTC16	7	82585533	C	T	PCLO	Missense_Mutation	p.R1579K	0.13	0.01	265	190	0.82	Subclonal	.	FALSE
MTC16	7	88963875	A	T	ZNF804B	Missense_Mutation	p.I527F	0.06	0.01	229	150	0.31	Subclonal	LOH	FALSE
MTC16	9	124535435	C	G	DAB2IP	Missense_Mutation	p.S848R	0.18	0.01	388	191	1	Clonal	LOH	FALSE
MTC16	9	134350896	G	A	PRRC2B	Missense_Mutation	p.R1127Q	0.14	0.01	364	182	0.9	Clonal	LOH	FALSE
MTC16	23	24766557	A	T	POLA1	Missense_Mutation	p.N935Y	0.10	0.00	202	115	0.54	Subclonal	LOH	FALSE
MTC16	23	53283878	G	A	IQSEC2	Missense_Mutation	p.S412L	0.11	0.00	311	165	0.59	Subclonal	LOH	FALSE
MTC19	1	62267402	AC	A	INADL	Frame_Shift_Del	p.T504Ifs*2	0.30	0.00	218	96	1	Clonal	.	FALSE
MTC19	1	109823794	C	T	PSRC1	Missense_Mutation	p.R200Q	0.05	0.00	718	211	0.5	Subclonal	.	FALSE
MTC19	1	114968215	C	G	TRIM33	Missense_Mutation	p.Q517H	0.05	0.00	213	111	0.28	Subclonal	.	FALSE
MTC19	1	154218791	T	A	UBAP2L	Missense_Mutation	p.N318K	0.05	0.01	295	132	0.4	Subclonal	.	FALSE
MTC19	1	158651439	G	A	SPTA1	Missense_Mutation	p.R137C	0.57	0.00	222	79	1	Clonal	.	FALSE
MTC19	1	176564357	C	A	PAPPA2	Missense_Mutation	p.N539K	0.28	0.01	388	164	1	Clonal	.	FALSE
MTC19	1	179533845	A	G	NPHS2	Missense_Mutation	p.S120P	0.06	0.00	192	66	0.42	Subclonal	.	FALSE
MTC19	10	50954876	C	T	OGDHL	Missense_Mutation	p.V406M	0.20	0.00	902	175	1	Clonal	.	FALSE
MTC19	10	68280476	T	G	CTNNA3	Missense_Mutation	p.N477T	0.16	0.00	815	147	1	Clonal	.	FALSE
MTC19	11	1619423	T	C	KRTAP5-2	Missense_Mutation	p.S20G	0.16	0.02	75	42	0.54	Subclonal	LOH	FALSE
MTC19	11	61109327	G	A	DAK	Missense_Mutation	p.V200I	0.03	0.00	406	203	0.21	Subclonal	.	FALSE
MTC19	11	111887496	C	G	DIXDC1	Missense_Mutation	p.P612R	0.34	0.01	191	146	1	Subclonal	LOH	FALSE
MTC19	12	3921499	A	T	PARP11	Missense_Mutation	p.H269Q	0.06	0.00	341	166	0.32	Subclonal	.	FALSE
MTC19	12	9305903	C	T	PZP	Missense_Mutation	p.G1271R	0.38	0.00	123	55	1	Clonal	.	FALSE
MTC19	12	23908626	C	G	SOX5	Missense_Mutation	p.D172H	0.04	0.00	216	99	0.2	Subclonal	.	FALSE
MTC19	12	49445224	C	A	KMT2D	Nonsense_Mutation	p.E748*	0.20	0.00	304	204	0.67	Subclonal	LOH	FALSE
MTC19	12	83251037	G	T	TMTC2	Missense_Mutation	p.G111V	0.68	0.00	363	320	1	Clonal	LOH	FALSE
MTC19	13	51854768	C	G	FAM124A	Missense_Mutation	p.H375Q	0.09	0.00	458	331	0.29	Subclonal	LOH	FALSE
MTC19	14	30068864	G	T	PRKD1	Missense_Mutation	p.Q689K	0.43	0.00	214	87	1	Clonal	.	FALSE
MTC19	16	773117	C	T	CCDC78	Missense_Mutation	p.R393Q	0.05	0.00	244	104	0.24	Subclonal	.	FALSE
MTC19	16	4861804	C	T	GLYR1	Splice_Site	p.X428_splice	0.05	0.00	264	109	0.25	Subclonal	.	FALSE
MTC19	16	78005778	G	C	VAT1L	Missense_Mutation	p.R370P	0.03	0.00	356	264	0.11	Subclonal	LOH	FALSE
MTC19	17	7481686	G	A	EIF4A1	Missense_Mutation	p.R368H	0.12	0.00	92	98	0.41	Subclonal	LOH	FALSE
MTC19	17	7578493	C	T	TP53	Nonsense_Mutation	p.W146*	0.55	0.00	132	119	1	Clonal	LOH	FALSE
MTC19	17	74868920	T	C	MGAT5B	Missense_Mutation	p.L30P	0.04	0.00	375	152	0.22	Subclonal	LOH	FALSE
MTC19	18	77896690	T	A	ADNP2	Nonstop_Mutation	p.*1132Kext*12	0.13	0.00	45	27	0.45	Subclonal	LOH	FALSE
MTC19	2	20454988	G	A	PUM2	Nonsense_Mutation	p.Q913*	0.44	0.00	175	101	1	Clonal	.	FALSE
MTC19	2	179549096	C	G	TTN	Missense_Mutation	p.V10895L	0.40	0.00	256	125	1	Clonal	.	FALSE
MTC19	2	231861028	C	A	SPATA3	Missense_Mutation	p.T27N	0.31	0.00	424	252	1	Clonal	.	FALSE
MTC19	20	47752392	C	T	STAU1	Missense_Mutation	p.R196Q	0.26	0.00	185	108	1	Clonal	.	FALSE
MTC19	22	19463121	G	T	UFD1L	Missense_Mutation	p.S3Y	0.09	0.00	218	140	0.38	Subclonal	LOH	FALSE
MTC19	3	37670789	C	T	ITGA9	Missense_Mutation	p.R601C	0.14	0.00	235	130	0.74	Subclonal	.	FALSE
MTC19	3	39942398	AAAG	A	MYRIP	In_Frame_Del	p.E34del	0.27	0.00	172	71	1	Clonal	.	FALSE
MTC19	3	143515676	G	C	SLC9A9	Missense_Mutation	p.L150V	0.32	0.00	165	74	1	Clonal	.	FALSE
MTC19	3	183957213	G	A	VWA5B2	Missense_Mutation	p.R745H	0.40	0.00	282	159	1	Clonal	.	FALSE
MTC19	5	170216227	A	C	GABRP	Missense_Mutation	p.K50Q	0.06	0.00	385	179	0.33	Subclonal	.	FALSE
MTC19	6	7373799	A	G	CAGE1	Missense_Mutation	p.V418A	0.05	0.00	429	200	0.28	Subclonal	.	FALSE
MTC19	6	30126446	A	C	TRIM10	Missense_Mutation	p.F64V	0.10	0.00	462	230	0.55	Subclonal	.	FALSE
MTC19	7	151856137	C	A	KMT2C	Missense_Mutation	p.M3827I	0.07	0.00	197	78	0.38	Subclonal	.	FALSE
MTC19	8	2088746	T	C	MYOM2	Missense_Mutation	p.Y1301H	0.36	0.00	182	92	1	Clonal	.	FALSE
MTC19	23	135292068	C	G	FHL1	Missense_Mutation	p.D309E	0.08	0.00	130	80	0.29	Subclonal	LOH	FALSE
MTC20	1	2572958	A	G	TTC34	Missense_Mutation	p.L517P	0.23	0.00	159	56	1	Clonal	.	FALSE
MTC20	1	6662835	G	A	KLHL21	Missense_Mutation	p.P15S	0.24	0.00	246	60	1	Clonal	.	FALSE
MTC20	1	17949566	G	A	ARHGEF10L	Missense_Mutation	p.V366M	0.20	0.00	240	94	0.91	Clonal	.	FALSE
MTC20	1	27099926	G	C	ARID1A	Missense_Mutation	p.G1269R	0.06	0.00	305	65	0.25	Subclonal	.	FALSE
MTC20	1	37941482	G	T	ZC3H12A	Nonsense_Mutation	p.E129*	0.08	0.00	427	79	0.51	Subclonal	.	FALSE
MTC20	1	51768188	C	G	TTC39A	Missense_Mutation	p.R278P	0.11	0.00	524	97	0.93	Clonal	.	FALSE
MTC20	1	190195266	G	C	BRINP3	Missense_Mutation	p.L303V	0.41	0.00	193	61	1	Clonal	.	FALSE
MTC20	1	204946815	AG	A	NFASC	Frame_Shift_Del	p.A614Pfs*23	0.46	0.00	599	152	1	Clonal	.	FALSE
MTC20	1	215260014	T	A	KCNK2	Missense_Mutation	p.L117H	0.17	0.00	374	90	1	Clonal	.	FALSE
MTC20	1	233372676	G	A	PCNXL2	Missense_Mutation	p.P758L	0.04	0.00	298	65	0.33	Subclonal	.	FALSE
MTC20	1	244582136	C	A	ADSS	Nonsense_Mutation	p.G291*	0.13	0.00	314	72	1	Clonal	.	FALSE
MTC20	10	51586674	A	G	NCOA4	Nonstop_Mutation	p.*651Wext*1	0.35	0.00	109	30	1	Clonal	LOH	FALSE
MTC20	10	75158092	T	A	ANXA7	Missense_Mutation	p.S36C	0.09	0.00	401	90	0.53	Subclonal	LOH	FALSE
MTC20	10	89685314	TGTAA	T	PTEN	Splice_Site	p.X70_splice	0.52	0.00	147	42	1	Clonal	LOH	FALSE
MTC20	11	5274541	C	G	HBG2	Missense_Mutation	p.G137A	0.08	0.00	92	23	0.35	Subclonal	LOH	FALSE
MTC20	11	22396308	T	G	SLC17A6	Missense_Mutation	p.M350R	0.14	0.00	209	94	0.65	Subclonal	LOH	FALSE
MTC20	11	45246339	GC	G	PRDM11	Frame_Shift_Del	p.V441Yfs*22	0.20	0.00	472	174	0.9	Clonal	LOH	FALSE
MTC20	11	57310474	C	A	SMTNL1	Missense_Mutation	p.S138Y	0.48	0.00	170	58	1	Clonal	LOH	FALSE
MTC20	11	62415826	G	A	INTS5	Missense_Mutation	p.R576C	0.13	0.00	398	137	0.6	Subclonal	LOH	FALSE
MTC20	11	64785689	A	G	ARL2	Missense_Mutation	p.D40G	0.05	0.00	118	40	0.23	Subclonal	LOH	FALSE
MTC20	11	65618586	C	T	SNX32	Nonsense_Mutation	p.R222*	0.04	0.00	552	155	0.24	Subclonal	.	FALSE
MTC20	11	103070182	C	A	DYNC2H1	Missense_Mutation	p.Q2689K	0.15	0.00	252	92	0.67	Subclonal	LOH	FALSE
MTC20	12	5021802	G	A	KCNA1	Missense_Mutation	p.E420K	0.07	0.00	276	85	0.31	Subclonal	.	FALSE
MTC20	12	56999794	CTT	C	BAZ2A	Frame_Shift_Del	p.K773Efs*23	0.23	0.00	193	63	1	Clonal	.	FALSE
MTC20	12	86270479	G	C	NTS	Missense_Mutation	p.K45N	0.20	0.00	187	65	0.92	Clonal	.	FALSE
MTC20	12	113901103	C	G	LHX5	Missense_Mutation	p.E367D	0.15	0.00	280	94	0.7	Subclonal	.	FALSE
MTC20	14	45658291	C	T	FANCM	Missense_Mutation	p.A1689V	0.05	0.00	350	121	0.22	Subclonal	.	FALSE
MTC20	14	105206963	G	A	ADSSL1	Missense_Mutation	p.G211R	0.65	0.01	816	126	1	Clonal	LOH	FALSE
MTC20	15	75108549	G	A	LMAN1L	Missense_Mutation	p.R76Q	0.10	0.00	90	35	0.46	Subclonal	LOH	FALSE
MTC20	15	82575017	C	G	FAM154B	Missense_Mutation	p.Q271E	0.15	0.00	291	89	0.69	Subclonal	LOH	FALSE
MTC20	16	27761148	A	G	KIAA0556	Missense_Mutation	p.Y956C	0.16	0.00	323	89	1	Clonal	.	FALSE
MTC20	16	30537665	G	T	ZNF768	Missense_Mutation	p.P24T	0.15	0.00	553	122	0.99	Clonal	.	FALSE
MTC20	16	50256319	T	A	PAPD5	Missense_Mutation	p.F366I	0.16	0.00	520	146	1	Clonal	.	FALSE
MTC20	17	7578279	AG	A	TP53	Frame_Shift_Del	p.P190Lfs*57	0.40	0.00	246	86	1	Clonal	LOH	FALSE
MTC20	17	33457308	C	A	FNDC8	Missense_Mutation	p.T277N	0.19	0.00	320	112	0.85	Subclonal	LOH	FALSE
MTC20	17	40843295	G	A	CNTNAP1	Missense_Mutation	p.A734T	0.25	0.00	226	86	1	Clonal	LOH	FALSE
MTC20	17	78013909	G	T	CCDC40	Missense_Mutation	p.S131I	0.03	0.00	230	84	0.16	Subclonal	LOH	FALSE
MTC20	17	79102319	C	T	AATK	Missense_Mutation	p.R122Q	0.24	0.00	121	37	1	Clonal	LOH	FALSE
MTC20	18	31432855	A	T	NOL4	Nonsense_Mutation	p.L623*	0.06	0.00	314	77	0.32	Subclonal	.	FALSE
MTC20	18	46606075	A	G	DYM	Missense_Mutation	p.S83P	0.04	0.00	224	61	0.2	Subclonal	.	FALSE
MTC20	19	6306885	A	G	ACER1	Missense_Mutation	p.L212P	0.22	0.00	200	50	1	Clonal	.	FALSE
MTC20	19	18234944	C	T	MAST3	Missense_Mutation	p.R248W	0.10	0.00	398	115	0.53	Subclonal	.	FALSE
MTC20	19	35843198	C	A	FFAR1	Missense_Mutation	p.F248L	0.41	0.00	970	67	1	Clonal	.	FALSE
MTC20	19	38973736	G	C	RYR1	Missense_Mutation	p.E1564Q	0.15	0.00	690	150	1	Clonal	.	FALSE
MTC20	19	50370425	C	G	PNKP	Missense_Mutation	p.E13Q	0.12	0.00	107	33	0.55	Subclonal	.	FALSE
MTC20	19	58102036	G	A	ZIK1	Missense_Mutation	p.R286Q	0.19	0.00	285	119	0.86	Clonal	.	FALSE
MTC20	2	24285943	G	A	FKBP1B	Missense_Mutation	p.G70R	0.09	0.00	392	81	0.57	Subclonal	.	FALSE
MTC20	2	137988779	G	A	THSD7B	Missense_Mutation	p.R630K	0.35	0.00	288	86	1	Clonal	.	FALSE

Supplementary Table S2 - Page 5 of 5

MTC20	2	165755162	G	A	SLC38A11	Missense_Mutation	p.H336Y	0.27	0.00	349	87	1	Clonal	.	FALSE
MTC20	2	168726259	C	T	B3GALT1	Missense_Mutation	p.S237L	0.16	0.00	360	96	1	Clonal	.	FALSE
MTC20	20	13830875	C	T	SEL1L2	Missense_Mutation	p.E524K	0.13	0.00	334	69	0.7	Subclonal	.	FALSE
MTC20	20	21376958	C	T	NKX2-4	Missense_Mutation	p.R219H	0.03	0.00	506	151	0.15	Subclonal	.	FALSE
MTC20	20	35860788	C	A	RPN2	Missense_Mutation	p.F557L	0.15	0.00	234	62	0.83	Subclonal	.	FALSE
MTC20	20	44588886	C	T	ZNF335	Missense_Mutation	p.E661K	0.15	0.00	350	109	0.86	Clonal	.	FALSE
MTC20	21	22910276	T	C	NCAM2	Nonstop_Mutation	p.*838Qext*18	0.05	0.00	427	133	0.23	Subclonal	.	FALSE
MTC20	21	31654562	G	A	KRTAP24-1	Missense_Mutation	p.S230F	0.03	0.00	328	113	0.14	Subclonal	.	FALSE
MTC20	3	9945781	C	T	IL17RE	Nonsense_Mutation	p.R72*	0.25	0.00	595	91	1	Clonal	.	FALSE
MTC20	3	42906721	G	A	ACKR2	Missense_Mutation	p.A243T	0.05	0.00	307	168	0.19	Subclonal	LOH	FALSE
MTC20	3	170150353	G	A	CLDN11	Missense_Mutation	p.A145T	0.25	0.00	331	78	1	Clonal	.	FALSE
MTC20	4	21950226	G	A	KCNIP4	Missense_Mutation	p.S10L	0.11	0.00	324	127	0.52	Subclonal	LOH	FALSE
MTC20	4	30723838	C	T	PCDH7	Missense_Mutation	p.A265V	0.39	0.00	157	45	1	Clonal	LOH	FALSE
MTC20	4	36230603	G	C	ARAP2	Missense_Mutation	p.S169C	0.15	0.01	259	107	0.7	Subclonal	LOH	FALSE
MTC20	4	85742362	A	T	WDFY3	Missense_Mutation	p.F489Y	0.06	0.00	264	107	0.26	Subclonal	LOH	FALSE
MTC20	5	124080311	C	G	ZNF608	Missense_Mutation	p.L124F	0.12	0.00	415	114	0.54	Subclonal	.	FALSE
MTC20	5	138209354	GAGTTTA	G	LRRTM2	In_Frame_Del	p.L297_N298del	0.05	0.00	506	155	0.24	Subclonal	.	FALSE
MTC20	5	140474755	C	A	PCDHB2	Missense_Mutation	p.D127E	0.08	0.00	342	124	0.39	Subclonal	.	FALSE
MTC20	5	140627349	C	T	PCDHB15	Missense_Mutation	p.H735Y	0.06	0.00	115	44	0.28	Subclonal	.	FALSE
MTC20	6	38142831	G	A	BTBD9	Missense_Mutation	p.A590V	0.22	0.00	329	95	1	Clonal	.	FALSE
MTC20	6	89967693	C	T	GABRR2	Missense_Mutation	p.C365Y	0.08	0.00	500	127	0.5	Subclonal	.	FALSE
MTC20	6	90384229	C	T	MDN1	Missense_Mutation	p.D4281N	0.26	0.00	89	18	1	Clonal	.	FALSE
MTC20	6	134210806	C	T	TCF21	Missense_Mutation	p.R91C	0.20	0.00	595	143	1	Clonal	.	FALSE
MTC20	7	22190089	A	G	RAPGEF5	Missense_Mutation	p.I454T	0.10	0.00	262	55	0.65	Subclonal	.	FALSE
MTC20	7	94827794	G	A	PPP1R9A	Missense_Mutation	p.E630K	0.50	0.00	111	21	1	Clonal	LOH	FALSE
MTC20	7	141442026	A	T	SSBP1	Nonsense_Mutation	p.R28*	0.05	0.00	279	72	0.31	Subclonal	.	FALSE
MTC20	8	2820036	C	A	CSMD1	Missense_Mutation	p.V3194F	0.28	0.00	262	116	0.99	Clonal	LOH	FALSE
MTC20	8	77766535	G	A	ZFHX4	Missense_Mutation	p.G2460R	0.04	0.00	573	136	0.18	Subclonal	.	FALSE
MTC20	8	87491210	T	C	RMDN1	Missense_Mutation	p.Y224C	0.34	0.00	172	49	1	Clonal	.	FALSE
MTC20	8	95521968	C	T	KIAA1429	Missense_Mutation	p.R1276H	0.04	0.00	272	80	0.2	Subclonal	.	FALSE
MTC20	9	34339065	A	T	NUDT2	Missense_Mutation	p.I10F	0.47	0.00	228	91	1	Clonal	.	FALSE
MTC20	9	105767696	G	C	CYLC2	Missense_Mutation	p.K261N	0.11	0.00	447	166	0.51	Subclonal	LOH	FALSE
MTC20	9	137701032	G	A	COL5A1	Missense_Mutation	p.E1124K	0.24	0.00	202	59	1	Clonal	LOH	FALSE
MTC20	23	105451146	T	C	MUM1L1	Missense_Mutation	p.V574A	0.03	0.00	474	143	0.18	Subclonal	.	FALSE
MTC20	23	142716989	T	G	SLITRK4	Missense_Mutation	p.N646H	0.23	0.00	324	85	1	Clonal	.	FALSE
MTC23	1	2573070	C	T	TTC34	Missense_Mutation	p.E480K	0.12	0.00	209	109	0.35	Subclonal	.	FALSE
MTC23	1	16257350	G	A	SPEN	Missense_Mutation	p.D1539N	0.27	0.00	394	232	0.79	Subclonal	.	FALSE
MTC23	1	63044638	C	G	DOCK7	Splice_Site	p.X624_splice	0.10	0.00	102	64	0.29	Subclonal	.	FALSE
MTC23	1	113244235	ACCTC	A	RHOC	Frame_Shift_Del	p.E169Cfs*51	0.46	0.00	167	87	1	Clonal	.	FALSE
MTC23	1	150443745	G	A	RPRD2	Missense_Mutation	p.R774K	0.11	0.00	446	232	0.44	Subclonal	LOH	FALSE
MTC23	1	207317258	G	T	C4BPA	Missense_Mutation	p.G514C	0.18	0.00	124	90	0.7	Subclonal	LOH	FALSE
MTC23	10	44052669	C	T	ZNF239	Missense_Mutation	p.G287S	0.53	0.00	457	184	1	Clonal	.	FALSE
MTC23	11	63753778	G	T	AP000721.4	Missense_Mutation	p.A94S	0.18	0.00	395	207	0.87	Clonal	.	FALSE
MTC23	11	117789327	T	C	TMPRSS13	Missense_Mutation	p.Q83R	0.38	0.00	24	19	1	Clonal	.	FALSE
MTC23	11	123624985	C	T	OR6X1	Missense_Mutation	p.G81E	0.31	0.00	376	224	0.91	Clonal	.	FALSE
MTC23	11	124135294	C	T	OR8G5	Missense_Mutation	p.A191V	0.30	0.01	305	165	0.87	Clonal	.	FALSE
MTC23	12	48062719	T	C	RPAP3	Missense_Mutation	p.M565V	0.35	0.00	144	102	1	Clonal	LOH	FALSE
MTC23	12	57910320	G	T	MARS	Nonsense_Mutation	p.E887*	0.60	0.00	247	159	1	Clonal	LOH	FALSE
MTC23	12	81769690	C	T	PPFIA2	Missense_Mutation	p.R339K	0.65	0.00	80	74	1	Clonal	LOH	FALSE
MTC23	12	120110112	G	C	PRKAB1	Missense_Mutation	p.E56Q	0.55	0.00	80	42	1	Clonal	.	FALSE
MTC23	13	29598948	A	G	MTUS2	Missense_Mutation	p.N48S	0.71	0.00	393	256	1	Clonal	LOH	FALSE
MTC23	13	113530151	G	A	ATP11A	Missense_Mutation	p.A1075T	0.80	0.00	311	109	1	Clonal	LOH	FALSE
MTC23	14	52741596	C	T	PTGDR	Missense_Mutation	p.R332W	0.14	0.00	306	191	0.4	Subclonal	.	FALSE
MTC23	14	105957743	G	A	C14orf80	Missense_Mutation	p.A11T	0.29	0.00	146	92	0.86	Clonal	.	FALSE
MTC23	16	2203047	G	C	RAB26	Splice_Site	p.X197_splice	0.43	0.01	303	176	1	Clonal	.	FALSE
MTC23	16	3021803	G	A	PAQR4	Missense_Mutation	p.E226K	0.29	0.00	374	209	1	Clonal	.	FALSE
MTC23	16	53493456	A	G	RBL2	Missense_Mutation	p.S425G	0.37	0.00	201	80	1	Clonal	.	FALSE
MTC23	17	7574034	C	G	TP53	Splice_Site	p.X332_splice	0.61	0.00	197	174	1	Clonal	LOH	FALSE
MTC23	19	6897538	C	T	EMR1	Missense_Mutation	p.S165F	0.60	0.00	344	212	1	Clonal	LOH	FALSE
MTC23	19	33693790	G	A	LRP3	Missense_Mutation	p.R53Q	0.08	0.00	90	70	0.23	Subclonal	LOH	FALSE
MTC23	2	9597137	C	T	CPSF3	Missense_Mutation	p.P560L	0.28	0.00	162	85	1	Clonal	.	FALSE
MTC23	2	33442635	C	T	LTBP1	Missense_Mutation	p.P573L	0.11	0.00	258	107	0.44	Subclonal	.	FALSE
MTC23	2	42508113	C	T	EML4	Missense_Mutation	p.A264V	0.29	0.01	145	72	1	Clonal	.	FALSE
MTC23	2	71791260	C	G	DYSF	Missense_Mutation	p.P828A	0.37	0.00	299	152	1	Clonal	.	FALSE
MTC23	2	74007178	G	A	DUSP11	Missense_Mutation	p.S22F	0.12	0.00	655	274	0.47	Subclonal	.	FALSE
MTC23	2	79971696	G	A	CTNNA2	Missense_Mutation	p.V96M	0.16	0.00	194	90	0.65	Subclonal	.	FALSE
MTC23	2	197208380	C	T	HECW2	Splice_Site	p.X134_splice	0.22	0.00	87	53	0.86	Clonal	.	FALSE
MTC23	21	45755647	G	A	C21orf2	Missense_Mutation	p.T46M	0.17	0.00	221	201	0.5	Subclonal	LOH	FALSE
MTC23	3	40208400	C	T	MYRIP	Missense_Mutation	p.A205V	0.30	0.00	111	71	0.87	Clonal	LOH	FALSE
MTC23	3	48456683	C	A	PLXNB1	Missense_Mutation	p.V1290F	0.66	0.01	302	197	1	Clonal	LOH	FALSE
MTC23	3	52643391	AC	A	PBRM1	Frame_Shift_Del	p.R835Lfs*20	0.36	0.00	124	129	0.7	Subclonal	LOH	FALSE
MTC23	3	178936082	G	A	PIK3CA	Missense_Mutation	p.E542K	0.26	0.00	242	107	1	Clonal	.	TRUE
MTC23	4	6884274	C	T	KIAA0232	Missense_Mutation	p.S722L	0.33	0.00	458	219	1	Clonal	.	FALSE
MTC23	4	110754357	C	T	RRH	Missense_Mutation	p.R57W	0.45	0.00	264	135	1	Clonal	.	FALSE
MTC23	4	184930697	C	T	STOX2	Missense_Mutation	p.P236S	0.49	0.00	426	239	1	Clonal	.	FALSE
MTC23	5	41058293	C	T	MROH2B	Missense_Mutation	p.V210I	0.10	0.00	129	74	0.5	Subclonal	.	FALSE
MTC23	5	180661716	CGT	C	TRIM41	Frame_Shift_Del	p.V613Lfs*41	0.19	0.00	363	229	0.74	Subclonal	.	FALSE
MTC23	6	43493626	G	A	XPO5	Missense_Mutation	p.S1006L	0.25	0.00	91	52	0.99	Clonal	.	FALSE
MTC23	9	20717823	A	T	FOCAD	Nonsense_Mutation	p.K30*	0.55	0.00	119	103	1	Clonal	LOH	FALSE
MTC23	9	116812389	G	A	ZNF618	Missense_Mutation	p.R843Q	0.24	0.00	440	238	0.95	Clonal	.	FALSE
MTC23	9	140115282	G	A	RNF208	Missense_Mutation	p.S128L	0.25	0.01	327	191	1	Clonal	.	FALSE
MTC23	23	39933386	G	T	BCOR	Missense_Mutation	p.P405T	0.14	0.00	449	270	0.4	Subclonal	.	FALSE
MTC23	23	70586227	G	A	TAF1	Missense_Mutation	p.M2I	0.51	0.01	486	181	1	Clonal	.	FALSE
MBC102T	1	976604	C	T	AGRN	Missense_Mutation	p.A260V	0.06	0.00	80	54	0.29	Subclonal	LOH	FALSE
MBC102T	1	11345789	C	T	UBIAD1	Missense_Mutation	p.Q206H	0.05	0.00	121	250	0.18	Subclonal	LOH	FALSE
MBC102T	1	43002234	G	C	CCDC30	Missense_Mutation	p.L27V	0.47	0.00	83	100	1	Clonal	.	FALSE
MBC102T	1	47610107	ATTTCTGGACATCC	A	CYP4A22	Frame_Shift_Del	p.F291Pfs*19	0.19	0.00	104	148	0.69	Subclonal	.	FALSE
MBC102T	1	54661260	G	T	CYB5RL	Missense_Mutation	p.D10E	0.26	0.00	119	182	0.94	Clonal	.	FALSE
MBC102T	1	110884300	C	A	RBM15	Missense_Mutation	p.T758N	0.06	0.01	109	156	0.25	Subclonal	.	FALSE
MBC102T	1	156948112	C	T	ARHGEF11	Missense_Mutation	p.L132	0.28	0.00	124	142	1	Clonal	LOH	FALSE
MBC102T	1	161168072	G	C	ADAMTS4	Missense_Mutation	p.P116A	0.13	0.00	252	245	0.87	Clonal	.	FALSE
MBC102T	1	223933044	G	A	CAPN2	Missense_Mutation	p.D155N	0.16	0.00	167	145	0.91	Clonal	.	FALSE
MBC102T	10	99619302	C	T	GOLGA7B	Nonsense_Mutation	p.Q34*	0.13	0.00	61	75	0.47	Subclonal	.	FALSE
MBC102T	12	2786274	G	A	CACNA1C	Missense_Mutation	p.V1663I	0.11	0.00	106	70	0.98	Clonal	.	FALSE
MBC102T	12	2794937	C	T	CACNA1C	Missense_Mutation	p.T1918M	0.10	0.00	98	68	0.88	Clonal	.	FALSE
MBC102T	12	25047325	C	G	BCAT1	Missense_Mutation	p.D67H	0.38	0.01	82	71	1	Clonal	.	FALSE
MBC102T	12	57350961	A	T	RDH16	Missense_Mutation	p.W96R	0.25	0.00	118	199	0.89	Clonal	LOH	FALSE
MBC102T	14	20344787	G	C	OR4K2	Missense_Mutation	p.D121H	0.17	0.00	64	63	0.96	Clonal	.	FALSE
MBC102T	14	65271717	C	G	SPTB	Missense_Mutation	p.K80N	0.12	0.00	99	143	0.44	Subclonal	LOH	FALSE
MBC102T	14	94423160	C	G	ASB2	Missense_Mutation	p.G88A	0.06	0.00	78	184	0.23	Subclonal	LOH	FALSE
MBC102T	15	91513704	C	G	PRC1	Missense_Mutation	p.S501T	0.16	0.00	44	61	0.57	Subclonal	LOH	FALSE
MBC102T	16	19195179	C	T	SYT17	Missense_Mutation	p.R221C	0.12	0.00	171	179	0.66	Subclonal	.	FALSE
MBC102T	16	66998318	C	G	CES3	Missense_Mutation	p.R207G	0.45	0.00	109	147	1	Clonal	.	FALSE
MBC102T	16	67680675	G	C	RLTPR	Missense_Mutation	p.E175D	0.42	0.00	76	97	1	Clonal	.	FALSE
MBC102T	16	88688688	G	A	ZC3H18	Missense_Mutation	p.R520H	0.31	0.00	151	184	1	Clonal	.	FALSE
MBC102T	16	89777057	C	T	VPS9D1	Missense_Mutation	p.V399I	0.17	0.00	135	171	0.79	Clonal	.	FALSE
MBC102T	17	7578275	G	A	TP53	Nonsense_Mutation	p.Q192*	0.45	0.00	84	89	1	Clonal	LOH	TRUE
MBC102T	17	16062142	G	C	NCOR1	Missense_Mutation	p.P222A	0.04	0.00	135	58	0.43	Subclonal	LOH	FALSE
MBC102T	17	74868849	A	T	MGAT5B	Missense_Mutation	p.K6N	0.12	0.00	139	103	0.65	Subclonal	LOH	FALSE
MBC102T	18	77623916	GC	G	KCNG2	Frame_Shift_Del	p.L84Ffs*113	0.11	0.00	182	70	0.95	Clonal	.	FALSE
MBC102T	19	8032593	T	G	ELAVL1	Missense_Mutation	p.K171T	0.05	0.00	146	146	0.22	Subclonal	.	FALSE
MBC102T	19	39361447	C	A	RINL	Missense_Mutation	p.V263F	0.17	0.00	143	209	0.98	Clonal	.	FALSE
MBC102T	19	40542138	GAGTA	G	ZNF780B	Frame_Shift_Del	p.T209Dfs*65	0.21	0.01	107	177	0.95	Clonal	.	FALSE
MBC102T	2	113514279	AGGAACTAAACTGTTCTT	A	CKAP2L	Frame_Shift_Del	p.K218*	0.15	0.00	40	85	0.69	Subclonal	.	FALSE
MBC102T	2	120362314	G	C	PCDP1	Missense_Mutation	p.M286I	0.39	0.00	79	73	1	Clonal	.	FALSE
MBC102T	2	169948316	T	G	DHRS9	Missense_Mutation	p.F197V	0.09	0.00	82	99	0.39	Subclonal	.	FALSE
MBC102T	2	219294178	C	T	VIL1	Missense_Mutation	p.E246D	0.25	0.00	151	170	1	Clonal	.	FALSE
MBC102T	2	228476110	C	G	C2orf83	Nonstop_Mutation	p.*151Yext*19	0.08	0.00	49	39	0.38	Subclonal	.	FALSE
MBC102T	2	239975279	AGGCAGCGCCAGTACTTGCCT	A	HDAC4	Splice_Site	p.X1025_splice	0.09	0.00	87	83	0.42	Subclonal	.	FALSE
MBC102T	20	8113356	G	T	PLCB1	Missense_Mutation	p.D20Y	0.49	0.00	80	40	1	Clonal	.	FALSE
MBC102T	20	36611937	G	C	TTI1	Missense_Mutation	p.P1064R	0.27	0.00	78	132	0.97	Clonal	LOH	FALSE
MBC102T	21	46057833	T	G	KRTAP10-10	Missense_Mutation	p.S167A	0.21	0.00	373	734	0.96	Clonal	.	FALSE
MBC102T	22	26224765	T	C	MYO18B	Missense_Mutation	p.S937P	0.26	0.00	74	135	0.93	Clonal	.	FALSE
MBC102T	3	45527206	TG	T	LARS2	Frame_Shift_Del	p.V348*	0.24	0.00	41	40	0.88	Clonal	LOH	FALSE
MBC102T	4	40810526	C	G	NSUN7	Missense_Mutation	p.A576G	0.31	0.00	55	108	1	Clonal	LOH	FALSE
MBC102T	4	71555718	A	G	UTP3	Missense_Mutation	p.R442G	0.16	0.00	86	198	0.91	Clonal	.	FALSE
MBC102T	4	72363374	A	T	SLC4A4	Nonsense_Mutation	p.K711*	0.12	0.00	49	55	0.69	Subclonal	.	FALSE
MBC102T	4	99862446	C	T	METAP1	Missense_Mutation	p.P380L	0.20	0.00	66	101	0.91	Clonal	.	FALSE
MBC102T	5	17275704	G	A	BASP1	Missense_Mutation	p.A127T	0.19	0.00	104	100	1	Clonal	.	FALSE
MBC102T	5	132161811	C	A	SHROOM1	Missense_Mutation	p.G8C	0.24	0.00	131	145	1	Clonal	LOH	FALSE
MBC102T	5	154394640	C	A	KIF4B	Missense_Mutation	p.S407R	0.08	0.00	79	132	0.35	Subclonal	LOH	FALSE
MBC102T	7	87022313	C	T	CROT	Nonsense_Mutation	p.R578*	0.17	0.00	47	78	0.62	Subclonal	.	FALSE
MBC102T	7	94293846	G	C	PEG10	Nonstop_Mutation	p.*326Yext*10	0.16	0.00	118	99	0.9	Clonal	.	FALSE
MBC102T	7	150440117	T	C	GIMAP5	Missense_Mutation	p.F297S	0.14	0.00	79	229	0.64	Subclonal	LOH	FALSE
MBC102T	8	139772490	T	C	COL22A1	Missense_Mutation	p.E633G	0.39	0.00	147	86	1	Clonal	.	FALSE
MBC102T	8	139772491	C	T	COL22A1	Missense_Mutation	p.E633K	0.39	0.00	147	87	1	Clonal	.	FALSE
MBC102T	9	135546017	C	T	GTF3C4	Missense_Mutation	p.P11L	0.51	0.02	68	63	1	Clonal	LOH	FALSE
MBC102T	9	139917178	C	T	ABCA2	Missense_Mutation	p.G139R	0.10	0.00	101	126	0.36	Subclonal	LOH	FALSE
MBC102T	23	148797822	A	G	MAGEA11	Missense_Mutation	p.I226V	0.42	0.00	77	66	1	Clonal	.	FALSE
MBC104T	1	41101696	C	T	RIMS3	Missense_Mutation	p.R84H	0.54	0.00	156	138	1	Clonal	LOH	FALSE
MBC104T	1	46476420	G	C	MAST2	Missense_Mutation	p.E333Q	0.22	0.00	101	54	1	Clonal	.	FALSE
MBC104T	1	49193564	C	A	BEND5	Missense_Mutation	p.L420F	0.18	0.00	92	68	0.92	Clonal	.	FALSE
MBC104T	1	52344255	A	C	NRD1	Missense_Mutation	p.C11W	0.16	0.01	193	120	1	Clonal	.	FALSE
MBC104T	1	75684202	A	T	SLC44A5	Missense_Mutation	p.L501H	0.11	0.00	165	64	0.87	Clonal	.	FALSE
MBC104T	1	98187211	G	T	DPYD	Missense_Mutation	p.A113D	0.10	0.00	58	41	0.62	Subclonal	.	FALSE
MBC104T	1	120458948	T	A	NOTCH2	Nonsense_Mutation	p.K2133*	0.34	0.00	146	75	1	Clonal	.	FALSE
MBC104T	1	156279007	C	G	CCT3	Missense_Mutation	p.D541H	0.48	0.00	90	62	1	Clonal	.	FALSE
MBC104T	1	161019106	C	G	ARHGAP30	Missense_Mutation	p.E569Q	0.04	0.00	256	188	0.21	Subclonal	.	FALSE
MBC104T	1	237791172	C	G	RYR2	Missense_Mutation	p.P2078A	0.05	0.00	170	88	0.32	Subclonal	.	FALSE
MBC104T	10	4872939	TACTTTTACC	T	AKR1E2	In_Frame_Del	p.F39_H41del	0.08	0.00	169	151	0.31	Subclonal	LOH	FALSE
MBC104T	10	43292265	A	C	BMS1	Missense_Mutation	p.S525R	0.09	0.00	222	147	0.51	Subclonal	.	FALSE
MBC104T	10	114053527	C	A	TECTB	Missense_Mutation	p.P172H	0.28	0.00	88	51	1	Clonal	.	FALSE
MBC104T	10	135107148	CGAGGAAGGTCCGGCTCTGCCTCCCAGCCAGGGGCT	C	TUBGCP2	Frame_Shift_Del	p.Q264Rfs*31	0.12	0.01	160	133	0.59	Subclonal	.	FALSE
MBC104T	11	34118154	C	A	CAPRIN1	Missense_Mutation	p.R612S	0.04	0.00	90	69	0.18	Subclonal	.	FALSE
MBC104T	11	62348952	C	CT	TUT1	Frame_Shift_Ins	p.L204Afs*44	0.18	0.00	271	177	0.9	Clonal	.	FALSE
MBC104T	11	67265777	C	A	PITPNM1	Missense_Mutation	p.H501Y	0.51	0.00	139	91	1	Clonal	.	FALSE
MBC104T	11	101953921	C	G	C11orf70	Missense_Mutation	p.D265E	0.45	0.00	77	36	1	Clonal	.	FALSE
MBC104T	11	113283577	T	C	DRD2	Missense_Mutation	p.E280G	0.53	0.00	191	123	1	Clonal	.	FALSE
MBC104T	11	118390386	A	C	KMT2A	Missense_Mutation	p.I3734L	0.08	0.00	86	76	0.49	Subclonal	.	FALSE
MBC104T	12	10312516	CTGAAGGCAGCTAAAA	C	OLR1	In_Frame_Del	p.I257_F261del	0.34	0.00	144	63	1	Clonal	.	FALSE
MBC104T	12	32974343	T	G	PKP2	Missense_Mutation	p.T698P	0.42	0.00	112	55	1	Clonal	.	FALSE
MBC104T	12	53188116	C	A	KRT3	Splice_Site	p.X216_splice	0.11	0.00	71	54	0.56	Subclonal	.	FALSE
MBC104T	12	56420748	C	T	IKZF4	Missense_Mutation	p.G157V	0.14	0.00	205	117	0.68	Subclonal	.	FALSE
MBC104T	12	96409421	C	G	LTA4H	Missense_Mutation	p.L333F	0.17	0.00	66	47	0.83	Clonal	.	FALSE
MBC104T	12	133197848	AAATACTACAAGATC	A	P2RX2	Frame_Shift_Del	p.Y306Wfs*16	0.25	0.00	122	72	0.98	Clonal	LOH	FALSE
MBC104T	14	24676640	TCTCAAAAAG	T	TSSK4	In_Frame_Del	p.K255_L257del	0.23	0.00	128	93	0.93	Clonal	LOH	FALSE
MBC104T	14	33069997	A	G	AKAP6	Missense_Mutation	p.K910R	0.34	0.00	95	61	1	Clonal	LOH	FALSE
MBC104T	14	103805071	G	A	EIF5	Splice_Site	p.X196_splice	0.09	0.00	265	103	0.85	Clonal	LOH	FALSE
MBC104T	14	104641533	GGGAGCTCACCGACAACGAA	G	KIF26A	Frame_Shift_Del	p.E604Vfs*12	0.22	0.00	292	129	1	Clonal	LOH	FALSE
MBC104T	15	38643275	A	C	SPRED1	Missense_Mutation	p.N249H	0.52	0.00	82	58	1	Clonal	LOH	FALSE
MBC104T	15	55611523	G	C	PIGB	Missense_Mutation	p.Q25H	0.48	0.00	124	99	1	Clonal	LOH	FALSE
MBC104T	15	77907402	C	T	LINGO1	Missense_Mutation	p.V283I	0.33	0.01	301	167	1	Clonal	.	FALSE
MBC104T	16	1390957	C	A	BAIAP3	Missense_Mutation	p.A183D	0.15	0.00	126	99	0.75	Subclonal	.	FALSE
MBC104T	16	2134665	A	G	TSC2	Missense_Mutation	p.K1481R	0.20	0.00	276	195	1	Clonal	.	FALSE
MBC104T	16	2347464	T	C	ABCA3	Missense_Mutation	p.Q710R	0.18	0.00	172	108	0.9	Clonal	.	FALSE
MBC104T	16	28506009	TTCCTCCGGCTATACC	T	APOBR	In_Frame_Del	p.L4_L8del	0.27	0.00	103	100	1	Clonal	.	FALSE
MBC104T	16	28962407	G	T	NFATC2IP	Missense_Mutation	p.W25C	0.05	0.00	261	174	0.23	Subclonal	.	FALSE
MBC104T	16	53340278	G	C	CHD9	Missense_Mutation	p.G2250A	0.58	0.00	64	31	1	Clonal	LOH	FALSE
MBC104T	16	57171070	G	A	CPNE2	Missense_Mutation	p.G393D	0.52	0.00	137	148	1	Clonal	LOH	FALSE
MBC104T	16	88039776	G	A	BANP	Missense_Mutation	p.S179N	0.24	0.00	259	189	1	Clonal	.	FALSE
MBC104T	16	89178609	C	A	ACSF3	Missense_Mutation	p.P311Q	0.17	0.00	140	91	0.85	Clonal	.	FALSE
MBC104T	17	7578344	GCAAGAAGCCCAGACGGAAAC	G	TP53	Frame_Shift_Del	p.G105Afs*34	0.41	0.00	116	104	1	Clonal	LOH	FALSE
MBC104T	17	39887953	C	G	HAP1	Missense_Mutation	p.Q313H	0.22	0.00	63	46	0.88	Clonal	LOH	FALSE
MBC104T	17	56233106	C	T	OR4D1	Missense_Mutation	p.L198F	0.11	0.00	240	120	0.9	Clonal	LOH	FALSE
MBC104T	18	31251786	A	C	ASXL3	Missense_Mutation	p.K224T	0.17	0.00	52	32	0.69	Subclonal	LOH	FALSE
MBC104T	18	44173717	G	C	LOXHD1	Missense_Mutation	p.P426R	0.25	0.00	95	38	1	Clonal	.	FALSE
MBC104T	19	5749111	G	C	CATSPERD	Splice_Site	p.X302_splice	0.26	0.00	53	47	1	Clonal	LOH	FALSE
MBC104T	19	15466034	C	T	AKAP8	Missense_Mutation	p.G591R	0.50	0.00	245	176	1	Clonal	LOH	FALSE
MBC104T	19	36394752	T	A	HCST	Missense_Mutation	p.L63H	0.27	0.00	181	143	1	Clonal	.	FALSE
MBC104T	19	45649203	G	T	PPP1R37	Missense_Mutation	p.G630V	0.08	0.00	143	99	0.38	Subclonal	.	FALSE
MBC104T	19	50795561	C	T	MYH14	Missense_Mutation	p.T1724I	0.16	0.00	153	105	0.81	Clonal	.	FALSE
MBC104T	19	58199279	CTCAT	C	ZNF551	Frame_Shift_Del	p.I547Sfs*136	0.09	0.01	317	190	0.47	Subclonal	.	FALSE
MBC104T	2	10917770	G	C	ATP6V1C2	Missense_Mutation	p.K295N	0.06	0.00	208	103	0.34	Subclonal	.	FALSE
MBC104T	2	55645971	T	A	CCDC88A	Missense_Mutation	p.N49Y	0.12	0.02	114	45	0.73	Subclonal	.	FALSE
MBC104T	2	84972256	G	A	DNAH6	Missense_Mutation	p.M3465I	0.12	0.00	83	47	0.48	Subclonal	.	FALSE
MBC104T	2	96781122	T	G	ADRA2B	Missense_Mutation	p.K256T	0.08	0.00	213	117	0.5	Subclonal	.	FALSE
MBC104T	2	107459810	C	T	ST6GAL2	Nonsense_Mutation	p.W208*	0.38	0.00	121	97	1	Clonal	.	FALSE
MBC104T	2	110372347	C	A	SOWAHC	Missense_Mutation	p.P94Q	0.04	0.00	136	109	0.18	Subclonal	.	FALSE
MBC104T	2	112917284	C	T	FBLN7	Missense_Mutation	p.A36V	0.34	0.01	170	166	1	Clonal	.	FALSE
MBC104T	2	182322473	G	T	ITGA4	Missense_Mutation	p.G31V	0.14	0.00	154	142	0.71	Subclonal	LOH	FALSE
MBC104T	2	200790110	G	A	C2orf69	Missense_Mutation	p.R220K	0.11	0.00	91	51	0.55	Subclonal	LOH	FALSE
MBC104T	20	56191400	T	G	ZBP1	Missense_Mutation	p.K53N	0.04	0.00	234	172	0.26	Subclonal	.	FALSE
MBC104T	21	32525401	A	T	TIAM1	Missense_Mutation	p.F1075I	0.20	0.00	50	49	0.79	Clonal	LOH	FALSE
MBC104T	21	46078008	G	T	KRTAP12-3	Missense_Mutation	p.V38L	0.17	0.00	535	289	1	Subclonal	.	FALSE
MBC104T	22	23915709	T	C	IGLL1	Missense_Mutation	p.N129S	0.38	0.00	81	103	1	Clonal	LOH	FALSE
MBC104T	3	53834314	CCGGCGTGCT	C	CACNA1D	In_Frame_Del	p.R1675_A1677del	0.14	0.00	70	43	0.57	Subclonal	LOH	FALSE
MBC104T	3	137787168	G	C	DZIP1L	Missense_Mutation	p.P553A	0.10	0.00	166	106	0.61	Subclonal	.	FALSE
MBC104T	3	190106206	A	T	CLDN16	Missense_Mutation	p.M100L	0.33	0.01	216	73	1	Clonal	.	FALSE
MBC104T	3	190031890	C	G	ATP13A5	Missense_Mutation	p.E751Q	0.19	0.00	226	87	1	Clonal	.	FALSE
MBC104T	4	9783750	G	C	DRD5	Missense_Mutation	p.A33P	0.59	0.00	125	128	1	Clonal	LOH	FALSE
MBC104T	4	84026135	G	T	PLAC8	Missense_Mutation	p.Q53K	0.10	0.01	133	76	0.58	Subclonal	.	FALSE
MBC104T	4	90856166	G	C	MMRN1	Missense_Mutation	p.L445F	0.15	0.00	122	48	0.88	Clonal	.	FALSE
MBC104T	4	103681939	A	G	MANBA	Missense_Mutation	p.L38P	0.05	0.01	182	107	0.3	Subclonal	.	FALSE
MBC104T	4	113436367	G	T	NEUROG2	Missense_Mutation	p.R89S	0.26	0.00	174	79	1	Clonal	.	FALSE
MBC104T	4	165961669	G	A	TRIM60	Missense_Mutation	p.L14I	0.20	0.00	64	39	0.81	Clonal	LOH	FALSE
MBC104T	5	178460049	G	A	ZNF879	Missense_Mutation	p.T367N	0.12	0.00	145	93	0.16	Subclonal	LOH	FALSE
MBC104T	6	27215772	G	A	PRSS16	Nonsense_Mutation	p.W61*	0.16	0.01	193	185	0.64	Subclonal	.	FALSE
MBC104T	6	30997218	C	G	MUC22	Missense_Mutation	p.T1337R	0.03	0.00	484	494	0.16	Subclonal	.	FALSE
MBC104T	6	31918441	C	A	CFB	Missense_Mutation	p.P1059H	0.21	0.00	121	104	1	Clonal	.	FALSE
MBC104T	6	74229735	C	G	EEF1A1	Missense_Mutation	p.K5N	0.35	0.00	178	104	1	Clonal	.	FALSE
MBC104T	7	193438	C	G	FAM20C	Missense_Mutation	p.A80G	0.05	0.00	88	59	0.18	Subclonal	LOH	FALSE
MBC104T	7	34118586	C	T	BMPER	Missense_Mutation	p.S399L	0.10	0.00	197	191	0.33	Subclonal	LOH	FALSE
MBC104T	7	92077150	G	A	GATAD1	Missense_Mutation	p.R36Q	0.41	0.00	103	96	1	Clonal	.	FALSE
MBC104T	7	94174998	C	A	CASD1	Missense_Mutation	p.Q540K	0.09	0.00	53	30	0.47	Subclonal	.	FALSE
MBC104T	7	102715738	G	A	ARMC10	Missense_Mutation	p.A7T	0.07	0.00	75	93	0.26	Subclonal	LOH	FALSE
MBC104T	7	127251588	C	A	PAX4	Splice_Site	p.X297_splice	0.35	0.00	106	69	1	Clonal	LOH	FALSE
MBC104T	7	128445863	G	A	CCDC136	Missense_Mutation	p.V333I	0.59	0.00	116	79	1	Clonal	LOH	FALSE
MBC104T	8	11282140	C	A	FAM167A	Missense_Mutation	p.E129D	0.40	0.00	154	204	1	Clonal	LOH	FALSE
MBC104T	8	74585450	T	A	STAU2	Missense_Mutation	p.N101I	0.13	0.00	104	51	0.75	Clonal	.	FALSE
MBC104T	8	77761236	A	G	ZFHX4	Missense_Mutation	p.I1173V	0.17	0.00	82	42	1	Clonal	.	FALSE
MBC104T	8	92090633	G	C	OTUD6B	Missense_Mutation	p.A152G	0.16	0.00	94	43	0.95	Clonal	.	FALSE
MBC104T	8	98973734	G	C	MATN2	Missense_Mutation	p.A312P	0.04	0.00	148	112	0.24	Subclonal	.	FALSE
MBC104T	8	119391936	C	T	SAMD12	Missense_Mutation	p.R109Q	0.13	0.00	142	58	0.8	Clonal	.	FALSE
MBC104T	8	144887169	G	A	SCRIB	Missense_Mutation	p.R896C	0.30	0.00	105	86	1	Clonal	LOH	FALSE
MBC104T	23	49961590	T	G	AKAP4	Missense_Mutation	p.E76D	0.55	0.00	60	44	1	Clonal	LOH	FALSE
MBC104T	23	79984382	C	T	BRWD3	Missense_Mutation	p.D419N	0.09	0.00	54	32	0.37	Subclonal	LOH	FALSE
MBC104T	23	106871867	TATCAAA	T	PRPS1	In_Frame_Del	p.K5_I6del	0.05	0.00	213	210	0.21	Subclonal	LOH	FALSE
MBC104T	23	132161873	CCTTCTGTGTTGTGCTAGAAAAGA	C	USP26	Frame_Shift_Del	p.V118Gfs*11	0.35	0.00	71	49	1	Clonal	LOH	FALSE
MBC108T	1	3672045	C	G	CCDC27	Missense_Mutation	p.S156C	0.41	0.00	69	106	1	Clonal	LOH	FALSE
MBC108T	1	16271190	A	G	ZBTB17	Splice_Site	p.X357_splice	0.19	0.00	243	257	1	Clonal	LOH	FALSE
MBC108T	1	47133814	C	G	ATPAF1	Missense_Mutation	p.D84H	0.08	0.00	99	92	0.45	Subclonal	LOH	FALSE
MBC108T	1	47571882	C	T	CYP4Z1	Missense_Mutation	p.R384W	0.50	0.00	90	108	1	Clonal	LOH	FALSE
MBC108T	1	66838030	G	A	PDE4B	Nonsense_Mutation	p.W627*	0.37	0.00	62	74	1	Clonal	LOH	FALSE
MBC108T	1	94474420	C	A	ABCA4	Nonsense_Mutation	p.E1908*	0.52	0.00	92	71	1	Clonal	LOH	FALSE
MBC108T	1	98293958	A	G	DPYD	Splice_Site	p.X78_splice	0.38	0.00	52	51	1	Clonal	LOH	FALSE
MBC108T	1	120469159	C	T	NOTCH2	Missense_Mutation	p.S1323N	0.08	0.01	143	79	0.89	Clonal	.	FALSE
MBC108T	1	158622360	G	A	SPTA1	Missense_Mutation	p.A1091V	0.08	0.00	151	109	0.68	Subclonal	.	FALSE
MBC108T	1	158813170	A	C	MNDA	Missense_Mutation	p.T123P	0.11	0.00	130	107	0.92	Clonal	.	FALSE
MBC108T	1	160206953	T	C	DCAF8	Missense_Mutation	p.I311V	0.11	0.00	106	68	0.96	Clonal	.	FALSE
MBC108T	1	175375714	T	C	TNR	Missense_Mutation	p.E46G	0.10	0.00	200	125	0.81	Clonal	.	FALSE
MBC108T	1	216380755	G	A	USH2A	Missense_Mutation	p.P1059L	0.09	0.00	150	94	0.98	Clonal	.	FALSE
MBC108T	1	222860330	ATCT	A	AIDA	In_Frame_Del	p.E133del	0.58	0.00	189	104	1	Clonal	.	FALSE
MBC108T	1	227946962	T	C	SNAP47	Missense_Mutation	p.V300A	0.11	0.00	230	117	1	Clonal	.	FALSE
MBC108T	1	235505468	T	G	GGPS1	Missense_Mutation	p.V95G	0.07	0.00	103	69	0.71	Clonal	.	FALSE
MBC108T	1	248458366	A	G	OR2T12	Missense_Mutation	p.I172T	0.10	0.00	714	323	1	Clonal	.	FALSE
MBC108T	10	5808019	C	T	GDI2	Missense_Mutation	p.D430N	0.42	0.00	93	56	1	Subclonal	.	FALSE
MBC108T	10	32322810	T	A	KIF5B	Missense_Mutation	p.E423V	0.20	0.00	85	55	1	Clonal	.	FALSE
MBC108T	10	49951199	T	C	WDFY4	Missense_Mutation	p.S689P	0.06	0.00	229	193	0.4	Subclonal	.	FALSE
MBC108T	10	101458523	T	C	ENTPD7	Missense_Mutation	p.F415L	0.20	0.00	147	123	1	Clonal	.	FALSE
MBC108T	10	102749600	G	GCCCGAC	C10orf2	In_Frame_Ins	p.P482_L483insDP	0.11	0.00	152	109	0.69	Subclonal	.	FALSE
MBC108T	10	103906731	G	A	PPRC1	Missense_Mutation	p.D1328N	0.33	0.00	243	193	1	Clonal	.	FALSE
MBC108T	11	1263814	G	C	MUC5B	Missense_Mutation	p.G1902R	0.20	0.00	429	483	1	Clonal	LOH	FALSE
MBC108T	11	34981976	G	T	PDHX	Missense_Mutation	p.L184F	0.13	0.00	63	57	0.83	Clonal	.	FALSE
MBC108T	11	55135778	T	C	OR4A15	Missense_Mutation	p.V140A	0.17	0.00	52	51	1	Clonal	.	FALSE
MBC108T	11	61912514	A	G	INCENP	Missense_Mutation	p.R556G	0.18	0.00	166	138	1	Clonal	.	FALSE
MBC108T	11	94911890	G	A	SESN3	Missense_Mutation	p.P347L	0.21	0.00	47	48	1	Clonal	.	FALSE
MBC108T	11	121454230	G	A	SORL1	Missense_Mutation	p.R1215Q	0.05	0.00	123	110	0.27	Subclonal	.	FALSE
MBC108T	12	56188620	C	G	RP11-762I7.5	Missense_Mutation	p.L429F	0.18	0.00	55	60	1	Clonal	.	FALSE
MBC108T	12	56231400	A	G	MMP19	Missense_Mutation	p.V376A	0.18	0.00	152	100	1	Clonal	.	FALSE
MBC108T	12	64813928	A	G	XPOT	Missense_Mutation	p.I190V	0.14	0.00	106	85	0.92	Clonal	.	FALSE
MBC108T	12	101480466	G	C	ANO4	Missense_Mutation	p.G487A	0.24	0.00	80	100	1	Clonal	.	FALSE
MBC108T	12	104200655	C	G	NT5DC3	Missense_Mutation	p.G149A	0.10	0.00	49	34	0.56	Subclonal	.	FALSE
MBC108T	12	113865879	G	T	SDSL	Missense_Mutation	p.G31V	0.17	0.01	155	145	0.92	Clonal	.	FALSE
MBC108T	13	77625291	G	C	MYCBP2	Missense_Mutation	p.P4550A	0.23	0.00	62	48	1	Clonal	.	FALSE
MBC108T	14	52781753	G	C	PTGER2	Missense_Mutation	p.V163L	0.07	0.00	245	179	0.48	Subclonal	LOH	FALSE
MBC108T	14	100134610	G	T	HHIPL1	Missense_Mutation	p.G567V	0.25	0.00	71	66	1	Clonal	LOH	FALSE
MBC108T	15	68583140	C	T	FEM1B	Missense_Mutation	p.R482C	0.17	0.00	98	58	1	Clonal	.	FALSE
MBC108T	15	75979931	G	A	CSPG4	Missense_Mutation	p.R1159C	0.20	0.01	216	130	1	Clonal	.	FALSE
MBC108T	16	3273954	G	T	ZNF200	Missense_Mutation	p.L376M	0.07	0.00	105	117	0.37	Subclonal	.	FALSE
MBC108T	16	3591887	C	T	NLRC3	Missense_Mutation	p.G1092R	0.07	0.00	69	58	0.4	Subclonal	.	FALSE
MBC108T	16	28507445	G	C	APOBR	Missense_Mutation	p.E361D	0.11	0.02	294	266	0.73	Subclonal	.	FALSE
MBC108T	16	28507452	G	T	APOBR	Missense_Mutation	p.G364W	0.08	0.02	274	264	0.55	Subclonal	.	FALSE
MBC108T	16	29908329	G	A	SEZ6L2	Missense_Mutation	p.P109S	0.24	0.00	149	126	1	Clonal	.	FALSE
MBC108T	16	48122501	T	A	ABCC12	Missense_Mutation	p.I1144L	0.12	0.00	124	117	0.79	Clonal	.	FALSE
MBC108T	16	50388798	C	G	BRD7	Missense_Mutation	p.E98D	0.33	0.00	107	98	1	Clonal	.	FALSE
MBC108T	16	71483768	C	T	ZNF23	Missense_Mutation	p.D54N	0.28	0.00	69	48	1	Clonal	.	FALSE
MBC108T	16	88497843	G	A	ZNF469	Missense_Mutation	p.R1294H	0.34	0.01	226	157	1	Clonal	.	FALSE
MBC108T	17	7577538	C	T	TP53	Missense_Mutation	p.R248Q	0.63	0.00	116	57	1	Clonal	LOH	TRUE
MBC108T	17	8722417	G	A	PIK3R6	Missense_Mutation	p.A659V	0.08	0.00	134	70	0.62	Subclonal	LOH	FALSE
MBC108T	17	11738075	G	A	DNAH9	Missense_Mutation	p.A3123T	0.04	0.00	289	167	0.31	Subclonal	LOH	FALSE
MBC108T	17	30538146	T	C	RHOT1	Missense_Mutation	p.L616S	0.12	0.00	67	53	0.9	Clonal	LOH	FALSE
MBC108T	17	40362499	C	T	STAT5B	Missense_Mutation	p.R566Q	0.09	0.00	75	62	0.7	Subclonal	LOH	FALSE
MBC108T	18	25591824	G	T	CDH2	Missense_Mutation	p.Q178K	0.16	0.00	51	49	0.86	Clonal	.	FALSE
MBC108T	18	67406208	G	A	DOK6	Missense_Mutation	p.D203N	0.19	0.00	77	90	1	Clonal	.	FALSE
MBC108T	18	67960342	T	C	SOCS6	Missense_Mutation	p.S480P	0.18	0.00	110	112	1	Clonal	.	FALSE
MBC108T	19	14721008	A	T	CLEC17A	Nonstop_Mutation	p.*379Cext*11	0.18	0.00	90	90	0.98	Clonal	.	FALSE
MBC108T	19	39214924	G	C	ACTN4	Missense_Mutation	p.G607A	0.05	0.00	277	246	0.3	Subclonal	.	FALSE
MBC108T	19	51892593	T	C	C19orf84	Missense_Mutation	p.H69R	0.08	0.00	185	180	0.45	Subclonal	.	FALSE
MBC108T	2	119726813	G	A	MARCO	Missense_Mutation	p.G59S	0.14	0.00	119	95	0.93	Clonal	.	FALSE
MBC108T	2	188532951	A	C	CSRNP3	Missense_Mutation	p.K180Q	0.09	0.00	111	119	0.59	Subclonal	.	FALSE
MBC108T	2	198544797	C	A	SLC39A10	Missense_Mutation	p.L11I	0.07	0.00	71	62	0.46	Subclonal	.	FALSE
MBC108T	2	196889169	G	C	DNAH7	Missense_Mutation	p.P243A	0.49	0.00	49	41	1	Subclonal	.	FALSE
MBC108T	20	33447370	C	T	GGT7	Missense_Mutation	p.R297H	0.18	0.00	164	153	0.28	Subclonal	.	FALSE
MBC108T	20	47835921	G	A	DDX27	Missense_Mutation	p.G10D	0.11	0.00	258	131	0.84	Clonal	.	FALSE
MBC108T	20	60348230	C	G	CDH4	Missense_Mutation	p.L190V	0.15	0.00	194	125	1	Clonal	.	FALSE
MBC108T	21	44837519	G	A	SIK1	Missense_Mutation	p.P627L	0.24	0.00	274	199	1	Clonal	.	FALSE
MBC108T	22	18613742	C	T	TUBA8	Missense_Mutation	p.L397F	0.15	0.00	456	380	0.99	Clonal	LOH	FALSE
MBC108T	22	21119214	T	A	PI4KA	Missense_Mutation	p.T867S	0.06	0.00	142	76	0.41	Subclonal	LOH	FALSE
MBC108T	22	38477653	A	C	SLC16A8	Missense_Mutation	p.L131R	0.14	0.00	296	121	1	Clonal	.	FALSE
MBC108T	22	38577373	T	C	KDELR3	Missense_Mutation	p.Y170H	0.10	0.00	245	77	1	Clonal	.	FALSE
MBC108T	22	39918353	A	G	ATF4	Missense_Mutation	p.M268V	0.22	0.00	388	123	1	Clonal	.	FALSE
MBC108T	3	38798317	C	T	SCN10A	Missense_Mutation	p.V380I	0.41	0.01	95	70	1	Clonal	.	FALSE
MBC108T	3	42680974	C	A	NKTR	Missense_Mutation	p.Q1260K	0.06	0.00	86	90	0.38	Subclonal	.	FALSE
MBC108T	3	47887748	C	A	DHX30	Missense_Mutation	p.P396T	0.04	0.01	197	164	0.23	Subclonal	.	FALSE
MBC108T	3	69087869	T	A	TMF1	Missense_Mutation	p.E666V	0.10	0.00	64	62	0.62	Subclonal	.	FALSE
MBC108T	3	77600065	G	A	ROBO2	Missense_Mutation	p.A402T	0.18	0.01	110	82	1	Clonal	.	FALSE
MBC108T	3	78684942	C	A	ROBO1	Missense_Mutation	p.E1118D	0.19	0.00	95	68	1	Clonal	.	FALSE
MBC108T	3	97202781	G	A	EPHA6	Missense_Mutation	p.R693H	0.09	0.01	75	76	0.51	Subclonal	.	FALSE
MBC108T	3	108748660	C	A	MORC1	Nonsense_Mutation	p.E548*	0.15	0.00	110	67	0.8	Clonal	.	FALSE
MBC108T	3	140407179	C	T	TRIM42	Missense_Mutation	p.G552V	0.05	0.00	197	142	0.25	Subclonal	.	FALSE
MBC108T	3	147113908	G	T	ZIC4	Missense_Mutation	p.T190N	0.05	0.00	513	279	0.34	Subclonal	.	FALSE
MBC108T	3	164777085	A	T	SI	Missense_Mutation	p.D383E	0.13	0.00	60	49	0.87	Clonal	.	FALSE
MBC108T	3	167035315	C	A	ZBBX	Missense_Mutation	p.A352S	0.21	0.00	62	44	1	Clonal	.	FALSE
MBC108T	3	186015273	C	G	DGKG	Splice_Site	p.X104_splice	0.15	0.00	54	41	0.96	Clonal	.	FALSE
MBC108T	4	1379733	A	T	UVSSA	Missense_Mutation	p.N705I	0.06	0.00	143	116	0.36	Subclonal	.	FALSE
MBC108T	4	57906995	T	C	IGFBP7	Missense_Mutation	p.N194D	0.08	0.00	50	39	0.6	Subclonal	.	FALSE
MBC108T	4	166000864	CTTACT	C	TMEM192	Frame_Shift_Del	p.S253Afs*16	0.15	0.00	73	93	0.83	Clonal	.	FALSE
MBC108T	4	185940788	A	T	HELT	Missense_Mutation	p.Q92L	0.18	0.00	85	75	0.97	Clonal	.	FALSE
MBC108T	4	190878562	G	C	FRG1	Missense_Mutation	p.A148P	0.07	0.01	131	156	0.38	Subclonal	.	FALSE
MBC108T	5	66386032	C	T	MAST4	Missense_Mutation	p.A269V	0.30	0.00	98	94	1	Clonal	.	FALSE
MBC108T	5	157106981	AC	A	C5orf52	Frame_Shift_Del	p.F153Sfs*19	0.30	0.00	90	89	1	Clonal	.	FALSE
MBC108T	5	178310976	C	T	ZNF354B	Missense_Mutation	p.S508L	0.15	0.00	123	141	0.81	Clonal	.	FALSE
MBC108T	6	32820908	G	A	TAP1	Missense_Mutation	p.S229F	0.13	0.00	346	209	1	Clonal	.	FALSE
MBC108T	6	36166207	G	T	BRPF3	Missense_Mutation	p.Q36H	0.03	0.00	358	178	0.29	Subclonal	.	FALSE
MBC108T	6	43013118	C	T	CUL7	Missense_Mutation	p.R1046Q	0.07	0.01	712	247	1	Clonal	.	FALSE
MBC108T	6	153077303	C	A	VIP	Missense_Mutation	p.R124S	0.06	0.00	90	77	0.36	Subclonal	.	FALSE
MBC108T	6	155569292	G	C	TIAM2	Missense_Mutation	p.E1271Q	0.06	0.00	234	191	0.36	Subclonal	.	FALSE
MBC108T	7	47409180	C	G	TNS3	Missense_Mutation	p.A355P	0.04	0.00	181	161	0.29	Subclonal	LOH	FALSE
MBC108T	7	111381624	C	G	DOCK4	Missense_Mutation	p.D1622H	0.05	0.00	132	93	0.39	Subclonal	.	FALSE
MBC108T	7	130025083	A	G	CPA1	Missense_Mutation	p.D295G	0.15	0.00	189	109	1	Clonal	.	FALSE
MBC108T	7	142609845	A	G	TRPV5	Missense_Mutation	p.F531L	0.10	0.00	201	112	0.89	Clonal	.	FALSE
MBC108T	8	77618911	T	G	ZFHX4	Missense_Mutation	p.L863R	0.08	0.00	419	99	1	Clonal	.	FALSE
MBC108T	8	77619990	G	T	ZFHX4	Nonsense_Mutation	p.E934*	0.06	0.00	305	99	0.92	Clonal	.	FALSE
MBC108T	8	87679312	C	T	CNGB3	Nonsense_Mutation	p.W231*	0.06	0.00	218	62	0.98	Clonal	.	FALSE
MBC108T	8	95444829	C	T	FSBP	Missense_Mutation	p.D144N	0.03	0.00	327	101	0.56	Subclonal	.	FALSE
MBC108T	8	98078339	C	T	CPQ	Nonsense_Mutation	p.E386*	0.81	0.00	268	81	1	Clonal	.	FALSE
MBC108T	8	105405047	C	A	DPYS	Nonsense_Mutation	p.E470*	0.08	0.01	359	111	1	Clonal	.	FALSE
MBC108T	8	110451290	G	A	PKHD1L1	Missense_Mutation	p.E1309K	0.10	0.00	187	53	1	Clonal	.	FALSE
MBC108T	8	126011849	T	G	SQLE	Missense_Mutation	p.D88E	0.04	0.00	706	186	0.72	Subclonal	.	FALSE
MBC108T	8	143556837	G	C	BAI1	Missense_Mutation	p.G263R	0.03	0.00	430	98	0.61	Subclonal	.	FALSE
MBC108T	9	34257647	C	T	KIF24	Missense_Mutation	p.R653H	0.54	0.00	167	114	1	Clonal	.	FALSE
MBC108T	23	55172537	G	A	FAM104B	Nonsense_Mutation	p.R111*	0.06	0.00	81	82	0.34	Subclonal	LOH	FALSE
MBC108T	23	99852621	A	G	TNMD	Missense_Mutation	p.T182A	0.07	0.00	67	59	0.41	Subclonal	LOH	FALSE
MBC114T	1	3329302	GC	G	PRDM16	Frame_Shift_Del	p.Q849Sfs*22	0.39	0.00	64	38	1	Clonal	.	FALSE
MBC114T	1	155340347	C	A	ASH1L	Nonsense_Mutation	p.E2212*	0.20	0.00	90	89	0.96	Clonal	.	FALSE
MBC114T	1	197112125	A	T	ASPM	Missense_Mutation	p.N419K	0.32	0.00	90	48	1	Clonal	.	FALSE
MBC114T	1	205273059	C	T	NUAK2	Missense_Mutation	p.G469E	0.50	0.01	119	154	1	Clonal	.	FALSE
MBC114T	1	236987448	C	A	MTR	Missense_Mutation	p.A265D	0.07	0.00	87	60	0.4	Subclonal	.	FALSE
MBC114T	10	75234701	C	T	PPP3CB	Missense_Mutation	p.E167K	0.12	0.00	58	30	0.58	Subclonal	.	FALSE
MBC114T	10	88233646	G	A	WAPAL	Nonsense_Mutation	p.Q577*	0.14	0.00	49	31	0.69	Subclonal	.	FALSE
MBC114T	10	91198779	G	A	SLC16A12	Missense_Mutation	p.R204W	0.55	0.00	119	125	1	Clonal	.	FALSE
MBC114T	11	14839889	C	A	PDE3B	Missense_Mutation	p.L555M	0.52	0.00	42	31	1	Clonal	LOH	FALSE
MBC114T	11	57235423	G	A	RTN4RL2	Missense_Mutation	p.D125N	0.05	0.00	535	135	1	Clonal	.	FALSE
MBC114T	11	75139544	C	T	KLHL35	Missense_Mutation	p.G337S	0.06	0.01	127	131	0.21	Subclonal	LOH	FALSE
MBC114T	12	117465850	G	A	FBXW8	Missense_Mutation	p.R557H	0.14	0.00	111	142	0.52	Subclonal	LOH	FALSE
MBC114T	12	119988783	G	A	CCDC60	Missense_Mutation	p.R489Q	0.65	0.00	57	60	1	Clonal	LOH	FALSE
MBC114T	13	47263332	G	A	LRCH1	Splice_Site	p.X338_splice	0.26	0.00	62	34	0.99	Clonal	.	FALSE
MBC114T	14	70924812	C	T	ADAM21	Missense_Mutation	p.S199F	0.07	0.00	87	83	0.33	Subclonal	.	FALSE
MBC114T	17	7578274	TGAGGAGGGGCCAGACCTAA	T	TP53	Splice_Site	p.X187_splice	0.28	0.00	74	130	1	Clonal	LOH	FALSE
MBC114T	17	31618521	C	A	ASIC2	Missense_Mutation	p.A205S	0.37	0.00	93	119	1	Clonal	.	FALSE
MBC114T	17	33769041	G	T	SLFN13	Missense_Mutation	p.T488N	0.11	0.00	365	555	0.42	Subclonal	.	FALSE
MBC114T	17	38975250	A	G	KRT10	Missense_Mutation	p.Y513H	0.18	0.01	516	434	0.85	Subclonal	.	FALSE
MBC114T	17	38975259	C	T	KRT10	Missense_Mutation	p.G510S	0.12	0.01	529	453	0.56	Subclonal	.	FALSE
MBC114T	17	48186714	C	T	PDK2	Nonsense_Mutation	p.Q345*	0.07	0.00	68	68	0.35	Subclonal	.	FALSE
MBC114T	18	74083505	C	T	ZNF516	Missense_Mutation	p.R1094Q	0.13	0.00	106	130	0.64	Subclonal	.	FALSE
MBC114T	19	362415	G	C	THEG	Missense_Mutation	p.Q309E	0.76	0.00	46	47	1	Subclonal	LOH	FALSE
MBC114T	19	10403740	C	T	ICAM5	Missense_Mutation	p.T428M	0.42	0.00	113	108	1	Clonal	.	FALSE
MBC114T	19	16614120	C	A	C19orf44	Missense_Mutation	p.S335Y	0.11	0.00	227	122	0.67	Subclonal	.	FALSE
MBC114T	19	16614123	C	T	C19orf44	Missense_Mutation	p.S336F	0.11	0.00	218	116	0.61	Subclonal	.	FALSE
MBC114T	19	17514547	TG	T	BST2	Frame_Shift_Del	p.Q167Sfs*3	0.50	0.00	187	169	1	Clonal	.	FALSE
MBC114T	19	37618268	C	G	ZNF420	Nonsense_Mutation	p.Y125*	0.41	0.00	108	98	1	Clonal	.	FALSE
MBC114T	19	47290985	C	G	SLC1A5	Missense_Mutation	p.L86F	0.39	0.00	89	56	1	Clonal	.	FALSE
MBC114T	2	27466140	G	T	CAD	Missense_Mutation	p.M2185I	0.29	0.01	87	79	1	Clonal	.	FALSE
MBC114T	2	47702224	G	A	MSH2	Missense_Mutation	p.S607N	0.08	0.00	65	55	0.37	Subclonal	.	FALSE
MBC114T	2	63169854	C	G	EHBP1	Missense_Mutation	p.P431R	0.21	0.00	68	46	0.99	Clonal	.	FALSE
MBC114T	2	121746170	G	T	GLI2	Missense_Mutation	p.A894S	0.40	0.00	53	49	1	Clonal	.	FALSE
MBC114T	2	121746171	C	G	GLI2	Missense_Mutation	p.A894G	0.38	0.00	52	49	1	Clonal	.	FALSE
MBC114T	2	228881801	C	G	SPHKAP	Missense_Mutation	p.A1257P	0.20	0.00	161	145	0.96	Clonal	.	FALSE
MBC114T	2	234545235	G	C	UGT1A10	Missense_Mutation	p.A23P	0.34	0.00	435	551	1	Clonal	.	FALSE
MBC114T	20	32210967	C	T	CBFA2T2	Missense_Mutation	p.A195V	0.43	0.01	156	154	1	Clonal	.	FALSE
MBC114T	20	43850374	G	A	SEMG2	Missense_Mutation	p.S34N	0.33	0.00	67	69	1	Clonal	.	FALSE
MBC114T	21	43531369	CCA	C	UMODL1	Frame_Shift_Del	p.Q680Gfs*46	0.42	0.00	482	501	1	Clonal	.	FALSE
MBC114T	21	44182290	G	A	PDE9A	Missense_Mutation	p.E395K	0.33	0.00	106	108	1	Clonal	.	FALSE
MBC114T	3	51746528	A	G	GRM2	Missense_Mutation	p.S164G	0.07	0.00	57	56	0.34	Subclonal	.	FALSE
MBC114T	4	73169659	G	A	ADAMTS3	Missense_Mutation	p.P800L	0.36	0.00	87	79	1	Clonal	.	FALSE
MBC114T	5	6605434	AACC	A	NSUN2	In_Frame_Del	p.M562_V563delinsI	0.37	0.00	126	146	1	Clonal	.	FALSE
MBC114T	5	38876329	C	T	OSMR	Missense_Mutation	p.P34S	0.12	0.00	43	32	0.44	Subclonal	.	FALSE
MBC114T	5	148897438	A	C	CSNK1A1	Missense_Mutation	p.C153W	0.60	0.00	47	42	1	Clonal	.	FALSE
MBC114T	5	150704970	G	T	SLC36A2	Missense_Mutation	p.P296Q	0.61	0.02	111	133	1	Clonal	.	FALSE
MBC114T	5	161300254	G	A	GABRA1	Missense_Mutation	p.H129Q	0.28	0.00	68	83	1	Clonal	.	FALSE
MBC114T	6	32157652	C	G	PBX2	Missense_Mutation	p.G14A	0.06	0.01	81	98	0.3	Subclonal	.	FALSE
MBC114T	6	33243782	A	G	RPS18	Missense_Mutation	p.Y77C	0.06	0.01	117	110	0.29	Subclonal	.	FALSE
MBC114T	6	46424599	G	A	RCAN2	Missense_Mutation	p.R39C	0.17	0.01	103	70	0.8	Clonal	.	FALSE
MBC114T	6	90661168	G	C	BACH2	Missense_Mutation	p.S219R	0.05	0.00	152	231	0.18	Subclonal	.	FALSE
MBC114T	7	112102163	C	G	IFRD1	Missense_Mutation	p.I242M	0.18	0.00	102	41	1	Clonal	.	FALSE
MBC114T	7	126883238	TC	T	GRM8	Frame_Shift_Del	p.R7Hfs*11	0.36	0.00	70	68	1	Clonal	.	FALSE
MBC114T	8	1949365	C	T	KBTBD11	Missense_Mutation	p.H3Y	0.62	0.00	52	74	1	Clonal	LOH	FALSE
MBC114T	8	95835706	C	T	INTS8	Missense_Mutation	p.P16L	0.08	0.00	344	134	0.91	Clonal	.	FALSE
MBC114T	8	144801238	C	T	MAPK15	Missense_Mutation	p.L165F	0.08	0.00	294	71	1	Clonal	.	FALSE
MBC117T	1	38397579	ACCTGCCTCCGCCAATTGTCG	A	INPP5B	Splice_Site	p.X173_splice	0.25	0.00	89	93	0.63	Subclonal	.	FALSE
MBC117T	1	151259282	T	G	ZNF687	Missense_Mutation	p.H165Q	0.19	0.01	331	161	0.87	Clonal	.	FALSE
MBC117T	1	154525263	C	A	UBE2Q1	Missense_Mutation	p.G245V	0.18	0.00	237	123	0.81	Subclonal	.	FALSE
MBC117T	1	155148118	A	G	TRIM46	Missense_Mutation	p.K107R	0.05	0.00	306	170	0.24	Subclonal	.	FALSE
MBC117T	1	176564421	T	G	PAPPA2	Missense_Mutation	p.Y561D	0.23	0.00	282	124	1	Clonal	.	FALSE
MBC117T	1	177225069	G	T	BRINP2	Missense_Mutation	p.W95L	0.20	0.00	157	77	0.93	Clonal	.	FALSE
MBC117T	1	214557444	T	A	PTPN14	Missense_Mutation	p.H585L	0.16	0.00	423	232	0.73	Subclonal	.	FALSE
MBC117T	1	222923290	G	A	FAM177B	Missense_Mutation	p.G123R	0.04	0.00	190	69	0.24	Subclonal	.	FALSE
MBC117T	1	235667524	G	C	B3GALNT2	Missense_Mutation	p.P10R	0.08	0.00	173	102	0.48	Subclonal	.	FALSE
MBC117T	1	237947659	C	T	RYR2	Missense_Mutation	p.A4216V	0.05	0.00	389	129	0.34	Subclonal	.	FALSE
MBC117T	1	240370934	G	C	FMN2	Missense_Mutation	p.G941A	0.17	0.02	502	147	1	Clonal	.	FALSE
MBC117T	1	247978959	G	A	OR14A16	Missense_Mutation	p.H25Y	0.60	0.02	95	44	1	Clonal	.	FALSE
MBC117T	10	43882956	T	G	HNRNPF	Missense_Mutation	p.E126A	0.09	0.00	302	156	0.32	Subclonal	LOH	FALSE
MBC117T	10	93008297	T	G	PCGF5	Missense_Mutation	p.L82R	0.11	0.00	96	63	0.41	Subclonal	LOH	FALSE
MBC117T	10	115406735	T	A	NRAP	Missense_Mutation	p.M314L	0.24	0.00	89	38	0.84	Clonal	LOH	FALSE
MBC117T	10	117049937	T	G	ATRNL1	Missense_Mutation	p.C725G	0.87	0.00	87	58	1	Clonal	LOH	FALSE
MBC117T	10	121411350	C	T	BAG3	Missense_Mutation	p.P55S	0.06	0.00	171	143	0.23	Subclonal	LOH	FALSE
MBC117T	11	18485554	T	C	LDHAL6A	Missense_Mutation	p.L58P	0.33	0.00	46	69	0.83	Clonal	LOH	FALSE
MBC117T	11	44957169	CCTT	C	TP53I11	In_Frame_Del	p.K126del	0.72	0.00	64	83	1	Clonal	LOH	FALSE
MBC117T	11	65810615	T	C	GAL3ST3	Missense_Mutation	p.D220G	0.83	0.00	102	125	1	Clonal	LOH	FALSE
MBC117T	11	94277903	G	A	FUT4	Missense_Mutation	p.D202N	0.16	0.00	70	74	0.4	Subclonal	LOH	FALSE
MBC117T	11	118415701	T	G	IFT46	Missense_Mutation	p.K333R	0.10	0.00	67	74	0.27	Subclonal	LOH	FALSE
MBC117T	12	6933930	T	C	GPR162	Missense_Mutation	p.L286W	0.83	0.00	76	64	1	Clonal	LOH	FALSE
MBC117T	12	9161632	G	T	KLRG1	Missense_Mutation	p.G140V	0.14	0.00	65	66	0.35	Subclonal	LOH	FALSE
MBC117T	12	23687427	G	A	SOX5	Missense_Mutation	p.A673V	0.08	0.01	155	92	0.3	Subclonal	.	FALSE
MBC117T	12	53012053	A	T	KRT73	Missense_Mutation	p.F86I	0.11	0.00	260	211	0.29	Subclonal	LOH	FALSE
MBC117T	12	53205596	G	T	KRT4	Missense_Mutation	p.Q210K	0.84	0.03	91	79	1	Clonal	LOH	FALSE
MBC117T	12	53709513	T	C	AAAS	Missense_Mutation	p.E102G	0.10	0.00	105	110	0.27	Subclonal	LOH	FALSE
MBC117T	12	56660951	G	A	COQ10A	Missense_Mutation	p.A17T	0.44	0.00	52	81	1	Clonal	LOH	FALSE
MBC117T	12	57573195	TCCTTCACGGTGC	T	LRP1	In_Frame_Del	p.F1609_P1612del	0.34	0.00	113	97	0.86	Clonal	LOH	FALSE
MBC117T	12	89745735	C	A	DUSP6	Nonsense_Mutation	p.E28*	0.82	0.00	234	238	1	Clonal	LOH	FALSE
MBC117T	12	111066649	G	C	TCTN1	Missense_Mutation	p.G184R	0.83	0.00	65	78	1	Clonal	LOH	FALSE
MBC117T	12	124882727	G	C	NCOR2	Missense_Mutation	p.A605G	0.07	0.00	97	89	0.18	Subclonal	LOH	FALSE
MBC117T	13	21264901	G	A	IFT88	Missense_Mutation	p.R776K	0.47	0.00	62	50	1	Clonal	.	FALSE
MBC117T	13	46840930	A	T	LRRC63	Missense_Mutation	p.K458M	0.08	0.00	83	61	0.3	Subclonal	.	FALSE
MBC117T	13	72440892	C	T	DACH1	Missense_Mutation	p.A6T	0.10	0.00	249	128	0.36	Subclonal	.	FALSE
MBC117T	13	99947349	T	C	GPR183	Missense_Mutation	p.M351V	0.06	0.00	129	90	0.22	Subclonal	.	FALSE
MBC117T	14	24527266	G	T	LRRC16B	Missense_Mutation	p.A439S	0.07	0.00	233	103	0.38	Subclonal	.	FALSE
MBC117T	14	68114890	A	G	ARG2	Missense_Mutation	p.I283M	0.13	0.00	72	94	0.32	Subclonal	LOH	FALSE
MBC117T	14	75747288	G	A	FOS	Missense_Mutation	p.R140K	0.08	0.00	95	117	0.22	Subclonal	LOH	FALSE
MBC117T	14	101200929	G	A	DLK1	Missense_Mutation	p.R283H	0.06	0.00	86	98	0.15	Subclonal	LOH	FALSE
MBC117T	14	104639497	C	T	KIF26A	Missense_Mutation	p.A335V	0.49	0.00	76	87	1	Clonal	LOH	FALSE
MBC117T	15	65890756	C	G	VWA9	Missense_Mutation	p.K160N	0.44	0.00	84	97	1	Clonal	LOH	FALSE
MBC117T	15	75632346	A	G	COMMD4	Nonstop_Mutation	p.*200Wext*52	0.10	0.00	89	117	0.26	Subclonal	LOH	FALSE
MBC117T	16	737052	C	T	WDR24	Missense_Mutation	p.G342S	0.90	0.01	315	195	1	Clonal	LOH	FALSE
MBC117T	16	4802448	T	C	ZNF500	Missense_Mutation	p.T458A	0.40	0.00	290	127	0.96	Clonal	LOH	FALSE
MBC117T	16	11370214	C	T	PRM2	Missense_Mutation	p.R5H	0.07	0.00	238	117	0.31	Subclonal	LOH	FALSE
MBC117T	16	31123302	G	A	BCKDK	Missense_Mutation	p.G350S	0.19	0.00	400	187	1	Clonal	LOH	FALSE
MBC117T	16	69368478	G	T	COG8	Nonsense_Mutation	p.C453*	0.08	0.00	130	104	0.22	Subclonal	LOH	FALSE
MBC117T	16	71692170	G	T	PHLPP2	Missense_Mutation	p.L749M	0.09	0.00	45	67	0.23	Subclonal	LOH	FALSE
MBC117T	16	89785495	A	T	VPS9D1	Missense_Mutation	p.Y39N	0.28	0.00	176	117	1	Clonal	LOH	FALSE
MBC117T	17	2908678	CGGGAA	C	RAP1GAP2	Frame_Shift_Del	p.E407Cfs*59	0.27	0.02	123	133	0.69	Subclonal	LOH	FALSE
MBC117T	17	6980062	C	G	CLEC10A	Missense_Mutation	p.V108L	0.22	0.00	143	127	0.55	Subclonal	LOH	FALSE
MBC117T	17	7577557	A	C	TP53	Missense_Mutation	p.C242G	0.91	0.00	87	73	1	Clonal	LOH	TRUE
MBC117T	17	8647456	G	C	CCDC42	Missense_Mutation	p.W43S	0.25	0.00	89	82	0.63	Subclonal	LOH	FALSE
MBC117T	17	27940406	C	T	ANKRD13B	Missense_Mutation	p.P563S	0.31	0.00	103	120	0.79	Subclonal	LOH	FALSE
MBC117T	17	27958521	C	G	SSH2	Missense_Mutation	p.D1204H	0.05	0.00	110	79	0.12	Subclonal	LOH	FALSE
MBC117T	17	39394506	C	T	KRTAP9-8	Missense_Mutation	p.P68L	0.03	0.00	581	499	0.12	Subclonal	LOH	FALSE
MBC117T	17	58987945	G	A	BCAS3	Missense_Mutation	p.G292D	0.31	0.00	155	76	1	Clonal	LOH	FALSE
MBC117T	17	74077379	G	A	ZACN	Missense_Mutation	p.A189T	0.08	0.00	241	118	0.44	Subclonal	LOH	FALSE
MBC117T	17	74287421	C	G	QRICH2	Missense_Mutation	p.L963F	0.06	0.00	218	94	0.33	Subclonal	LOH	FALSE
MBC117T	17	80195740	T	A	SLC16A3	Missense_Mutation	p.V365E	0.15	0.00	226	119	0.86	Clonal	LOH	FALSE
MBC117T	18	7014028	C	A	LAMA1	Missense_Mutation	p.G1050V	0.26	0.00	136	74	1	Clonal	.	FALSE
MBC117T	18	29049070	G	A	DSG3	Missense_Mutation	p.R552K	0.05	0.00	319	106	0.31	Subclonal	LOH	FALSE
MBC117T	19	12036086	A	G	ZNF700	Missense_Mutation	p.M21V	0.81	0.00	257	263	1	Clonal	LOH	FALSE
MBC117T	19	30934990	A	T	ZNF536	Missense_Mutation	p.K174M	0.08	0.00	106	140	0.19	Subclonal	LOH	FALSE
MBC117T	19	38379702	G	A	WDR87	Nonsense_Mutation	p.Q1498*	0.83	0.01	121	112	1	Clonal	LOH	FALSE
MBC117T	19	43430074	G	A	PSG7	Missense_Mutation	p.S365F	0.11	0.01	335	353	0.29	Subclonal	LOH	FALSE
MBC117T	19	50017367	C	G	FCGRT	Missense_Mutation	p.A101G	0.49	0.00	95	95	1	Clonal	LOH	FALSE
MBC117T	19	50212070	C	T	CPT1C	Missense_Mutation	p.R514W	0.79	0.00	70	68	1	Clonal	LOH	FALSE
MBC117T	19	50412409	G	C	NUP62	Missense_Mutation	p.P219R	0.36	0.00	159	172	0.92	Clonal	LOH	FALSE
MBC117T	2	32756601	C	G	BIRC6	Missense_Mutation	p.P4092A	0.13	0.00	126	76	0.62	Subclonal	.	FALSE
MBC117T	2	33482518	G	T	LTBP1	Nonsense_Mutation	p.E779*	0.57	0.00	251	86	1	Clonal	.	FALSE
MBC117T	2	37398729	A	C	SULT6B1	Missense_Mutation	p.L172R	0.37	0.00	182	74	1	Clonal	.	FALSE
MBC117T	2	54871668	CTGA	C	SPTBN1	In_Frame_Del	p.D1407del	0.39	0.02	253	129	1	Clonal	.	FALSE
MBC117T	2	71592630	C	T	ZNF638	Missense_Mutation	p.L597F	0.29	0.00	95	87	1	Clonal	.	FALSE
MBC117T	2	79348765	T	A	REG1A	Missense_Mutation	p.Y48N	0.31	0.00	187	96	1	Clonal	.	FALSE
MBC117T	2	102959578	G	C	IL1RL1	Missense_Mutation	p.G255R	0.33	0.01	83	74	1	Clonal	.	FALSE
MBC117T	2	136379131	AAAAAG	A	R3HDM1	Frame_Shift_Del	p.E126Gfs*3	0.64	0.00	107	72	1	Clonal	LOH	FALSE
MBC117T	2	179487459	G	T	TTN	Missense_Mutation	p.L14951I	0.09	0.00	58	57	0.31	Subclonal	LOH	FALSE
MBC117T	2	229890384	T	A	PID1	Missense_Mutation	p.E237D	0.14	0.00	132	84	0.49	Subclonal	LOH	FALSE
MBC117T	2	241537814	G	T	CAPN10	Missense_Mutation	p.E663D	0.10	0.00	72	49	0.35	Subclonal	LOH	FALSE
MBC117T	20	33057854	G	C	ITCH	Missense_Mutation	p.D517H	0.34	0.00	65	51	1	Clonal	.	FALSE
MBC117T	21	46932218	T	TG	COL18A1	Frame_Shift_Ins	p.R1492Qfs*20	0.13	0.00	118	88	0.45	Subclonal	.	FALSE
MBC117T	22	37524884	C	A	IL2RB	Missense_Mutation	p.W303L	0.17	0.01	198	91	0.78	Subclonal	.	FALSE
MBC117T	22	42271384	A	G	SREBF2	Missense_Mutation	p.K378R	0.85	0.00	136	85	1	Clonal	LOH	FALSE
MBC117T	22	50897947	C	A	SBF1	Missense_Mutation	p.G1214C	0.09	0.00	109	71	0.33	Subclonal	LOH	FALSE
MBC117T	3	9948451	C	A	IL17RE	Missense_Mutation	p.P143Q	0.25	0.00	198	100	0.88	Clonal	LOH	FALSE
MBC117T	3	18436180	A	T	SATB1	Missense_Mutation	p.L327Q	0.10	0.00	179	123	0.36	Subclonal	LOH	FALSE
MBC117T	3	47032790	G	C	NBEAL2	Missense_Mutation	p.G213A	0.48	0.00	128	128	1	Clonal	LOH	FALSE
MBC117T	3	52544212	C	G	STAB1	Missense_Mutation	p.N863K	0.10	0.00	117	130	0.26	Subclonal	LOH	FALSE
MBC117T	3	142841161	G	T	CHST2	Missense_Mutation	p.Q501H	0.04	0.00	168	83	0.15	Subclonal	.	FALSE
MBC117T	3	161214933	G	A	OTOL1	Missense_Mutation	p.G113E	0.43	0.00	197	123	1	Clonal	.	FALSE
MBC117T	4	39448196	G	T	KLB	Missense_Mutation	p.R617L	0.31	0.00	211	135	1	Clonal	.	FALSE
MBC117T	4	46967027	G	A	GABRA4	Missense_Mutation	p.A365V	0.50	0.00	117	85	1	Clonal	.	FALSE
MBC117T	4	57189675	G	A	KIAA1211	Missense_Mutation	p.R1107Q	0.53	0.00	220	94	1	Clonal	.	FALSE
MBC117T	4	144620393	C	T	FREM3	Missense_Mutation	p.G479E	0.89	0.00	160	106	1	Clonal	LOH	FALSE
MBC117T	4	152679995	G	A	PET112	Nonsense_Mutation	p.Q86*	0.34	0.00	56	68	0.87	Clonal	LOH	FALSE
MBC117T	5	95729037	TG	T	PCSK1	Frame_Shift_Del	p.S645Afs*7	0.18	0.00	117	118	0.46	Subclonal	LOH	FALSE
MBC117T	5	140209126	G	A	PCDHA6	Missense_Mutation	p.A464T	0.07	0.00	760	483	0.31	Subclonal	LOH	FALSE
MBC117T	5	180338395	C	G	BTNL8	Missense_Mutation	p.L152V	0.09	0.00	233	147	0.31	Subclonal	LOH	FALSE
MBC117T	6	30619204	T	A	C6orf136	Missense_Mutation	p.F423Y	0.25	0.00	272	139	1	Clonal	.	FALSE
MBC117T	6	30993623	A	G	MUC22	Missense_Mutation	p.T139A	0.66	0.00	328	159	1	Clonal	.	FALSE
MBC117T	6	32810012	T	TAAGTTCTCC	PSMB8	In_Frame_Ins	p.R145_I146insGEL	0.33	0.00	219	110	1	Clonal	.	FALSE
MBC117T	6	37186507	C	G	TMEM217	Missense_Mutation	p.W105C	0.21	0.00	189	114	0.96	Clonal	.	FALSE
MBC117T	6	41909281	T	A	CCND3	Missense_Mutation	p.E36V	0.08	0.00	138	96	0.36	Subclonal	.	FALSE
MBC117T	6	151152125	C	G	PLEKHG1	Missense_Mutation	p.D626E	0.44	0.00	116	67	1	Clonal	.	FALSE
MBC117T	7	45125157	G	T	NACAD	Missense_Mutation	p.L208M	0.08	0.00	61	98	0.13	Subclonal	LOH	FALSE
MBC117T	7	107748104	G	A	LAMB4	Missense_Mutation	p.S188L	0.44	0.00	78	57	1	Clonal	LOH	FALSE
MBC117T	7	143048969	C	A	CLCN1	Missense_Mutation	p.P960T	0.22	0.00	214	105	0.8	Subclonal	LOH	FALSE
MBC117T	8	48921031	G	T	UBE2V2	Splice_Site	p.X6_splice	0.06	0.01	354	155	0.37	Subclonal	.	FALSE
MBC117T	8	77619863	G	T	ZFHX4	Missense_Mutation	p.E891D	0.13	0.00	467	89	1	Clonal	.	FALSE
MBC117T	8	125464390	C	A	TRMT12	Missense_Mutation	p.Q408K	0.20	0.00	272	118	1	Clonal	.	FALSE
MBC117T	8	144461591	ATG	A	RHPN1	Frame_Shift_Del	p.F289*	0.28	0.00	231	99	1	Clonal	.	FALSE
MBC117T	8	144680553	G	GC	TIGD5	Frame_Shift_Ins	p.E162Rfs*173	0.13	0.00	149	81	1	Clonal	.	FALSE
MBC117T	9	386359	C	A	DOCK8	Missense_Mutation	p.S936Y	0.14	0.00	150	75	0.64	Subclonal	.	FALSE
MBC117T	9	8518054	C	T	PTPRD	Missense_Mutation	p.G446E	0.22	0.00	194	103	1	Clonal	.	FALSE
MBC117T	9	21333735	C	T	KLHL9	Missense_Mutation	p.R375Q	0.04	0.00	141	97	0.19	Subclonal	.	FALSE
MBC117T	9	33030543	A	G	DNAJA1	Missense_Mutation	p.Q174R	0.10	0.00	216	150	0.44	Subclonal	.	FALSE
MBC117T	9	77422976	A	T	TRPM6	Missense_Mutation	p.Y538N	0.09	0.00	75	68	0.24	Subclonal	LOH	FALSE
MBC117T	9	113149635	A	T	SVEP1	Missense_Mutation	p.S3330R	0.35	0.00	106	103	0.89	Clonal	LOH	FALSE
MBC117T	23	35820316	G	A	MAGEB16	Translation_Start_Site	p.M1?	0.18	0.00	89	97	0.28	Subclonal	LOH	FALSE
MBC117T	23	63412054	C	G	AMER1	Missense_Mutation	p.E371D	0.10	0.00	82	112	0.15	Subclonal	LOH	FALSE
MBC117T	23	70468535	C	A	ZMYM3	Splice_Site	p.X579_splice	0.33	0.00	64	67	0.51	Subclonal	LOH	FALSE
MBC117T	23	100745678	G	A	ARMCX4	Missense_Mutation	p.G701E	0.76	0.00	63	98	1	Clonal	LOH	FALSE
MBC117T	23	152686464	G	A	ZFP92	Missense_Mutation	p.C210Y	0.83	0.00	246	306	1	Clonal	LOH	FALSE
MBC117T	23	153770537	CTA	C	IKBKG	Frame_Shift_Del	p.I210fs*50	0.78	0.00	110	114	1	Clonal	LOH	FALSE

Supplementary Table S3: Comparison of dominant mutational signatures inferred by DeconstructSigs and SigMA in 22 metaplastic breast cancers subjected to whole-exome sequencing.

Sample ID*	Diagnosis	Total snvs	Signatures (SigMA)	Exposures (SigMA)	Category (SigMA)	Category by exposure (SigMA)	Dominant mutational signature (DeconstructSigs)
MP1	Metaplastic carcinoma with mesenchymal differentiation	154	Signature_3.Signature_1.Signature_2.Signature_13.Signature_18	88.6941058429972_18.6159835713464_15.6994916322547_17.2312732782683_15.1010254704223	HRD_hc	HRD	HRD
MP11	Carcinoma with squamous metaplasia	298	Signature_3.Signature_1.Signature_5.Signature_13.Signature_17	125.526356108847_21.9235289690788_107.735841811667_30.7298031429706_2.17075661416503	HRD_hc	Aging	HRD
MP15	Metaplastic carcinoma with mesenchymal differentiation	151	Signature_3.Signature_1.Signature_5.Signature_2.Signature_13	75.3223150931959_2.07741072965743_60.6277813799548_11.7560430439244_5.30141933075836	HRD_hc	HRD	HRD
MP17	Metaplastic spindle cell carcinoma	52	Signature_8.Signature_1.Signature_5.Signature_2.Signature_13	8.5027931104283_14.2845791053063_27.0999153880578_2.5398544419136_1.27162671223264	HRD_lc	Aging	Aging
MP18	Metaplastic carcinoma with mesenchymal differentiation	113	Signature_3.Signature_1.Signature_5.Signature_13.Signature_18	62.3933967502917_3.87448036003269_31.7156376682712_11.7490235676498_6.67175374391688	HRD_hc	HRD	HRD
MP19	Metaplastic carcinoma with mesenchymal differentiation	163	Signature_3.Signature_8.Signature_1.Signature_2.Signature_13	90.0573111850996_50.6832839197493_1.98423520267041_22.051924888077_6.77423309800758	HRD_hc	HRD	HRD
MP21	Metaplastic spindle cell carcinoma	89	Signature_3.Signature_1.Signature_5.Signature_2.Signature_13	35.6091363670376_7.25014962670663_45.7330612512224_1.35135265872495_0.922636844635545	HRD_hc	Aging	HRD
MP27	Metaplastic carcinoma with mesenchymal differentiation	135	Signature_3.Signature_1.Signature_5.Signature_2.Signature_13	44.2834422489728_10.6940566093257_70.2873961752368_5.47771901034223_2.33526511495734	HRD_hc	Aging	HRD
MP7	Metaplastic spindle cell carcinoma	108	Signature_8.Signature_1.Signature_5.Signature_13.Signature_18	19.2056813774794_20.6331118470695_37.6163716060211_6.56032841854187_22.5552169353329	HRD_lc	Aging	Aging
MP8	Metaplastic spindle cell carcinoma	156	Signature_3.Signature_1.Signature_5.Signature_2.Signature_13	105.943918886703_19.5516364932942_25.7673576870912_2.95711355968434_11.8956577525147	HRD_hc	HRD	HRD
MTC01	Metaplastic spindle cell carcinoma	32	Signature_8.Signature_1.Signature_5.Signature_2.Signature_13	6.81417136856702_7.04025672348374_18.8105255580141_0.861982696319017_1.40093645540727	HRD_lc	Aging	Aging
MTC03	Metaplastic spindle cell carcinoma	337	Signature_3.Signature_1.Signature_2.Signature_13	40.7022695986668_29.0727608079726_132.055355754668_125.999815761497	HRD_lc	APOBEC	APOBEC
MTC06	Metaplastic carcinoma with mesenchymal differentiation	250	Signature_3.Signature_1.Signature_5.Signature_2.Signature_13	86.0719360134096_31.7929598651662_62.4493725597839_21.4276607267504_41.2377836477811	HRD_hc	Aging	HRD
MTC07	Metaplastic carcinoma with mesenchymal differentiation	292	Signature_1.Signature_5.Signature_2.Signature_13.Signature_17	82.7835873393402_184.358023922446_10.8290664895516_4.90734009442309_1.59177031179373	Aging	Aging	Aging
MTC11	Metaplastic carcinoma with mesenchymal differentiation	125	Signature_8.Signature_1.Signature_5.Signature_2.Signature_13	23.7104167841709_15.6097635162883_69.2011408616789_10.2188100214276_1.43004865617695	HRD_lc	Aging	Aging
MTC12	Metaplastic squamous cell carcinoma	82	Signature_8.Signature_1.Signature_5.Signature_2.Signature_13	11.4845527229871_17.4063962805687_44.0384394741949_4.6201022489803_4.80609213092527	HRD_lc	Aging	Aging
MTC13	Metaplastic carcinoma with mesenchymal differentiation	54	Signature_1.Signature_5.Signature_2.Signature_18	25.1245424757485_25.6109497729029_6.55229283848049_0.778684605950905	Aging	Aging	Aging
MTC14	Metaplastic carcinoma with mesenchymal differentiation	159	Signature_3.Signature_1.Signature_5.Signature_2.Signature_13	94.1338609273824_18.8624211087819_34.5247951565215_5.69571557435469_9.64438803794576	HRD_hc	HRD	HRD
MTC16	Metaplastic squamous cell carcinoma	32	Signature_8.Signature_1.Signature_5.Signature_2	4.38018040346797_9.74450888788618_9.17646805833864_5.24344315374308	Aging	Aging	Aging
MTC19	Metaplastic carcinoma with mesenchymal differentiation	48	Signature_3.Signature_8.Signature_1.Signature_13.Signature_17	20.9154982620611_2.85193039254579_20.2370045412469_0.186810833295693_3.31605194813935	Aging	HRD	Aging
MTC20	Metaplastic squamous cell carcinoma	110	Signature_8.Signature_1.Signature_5.Signature_2.Signature_13	4.63136579798159_33.6394777936755_57.792019814215_4.06985987568192_10.346933218506	Aging	Aging	Aging
MTC23	Metaplastic squamous cell carcinoma	70	Signature_3.Signature_8.Signature_1.Signature_2.Signature_13	12.9799859250373_5.59883383918014_26.3053329195012_21.6540891496313_2.11778516882859	HRD_lc	Aging	Aging

* cases reported by Ng et al., 2017

HRD, homologous recombination DNA repair defect; SNV, single nucleotide variants; lc, low confidence; hc, high confidence

Supplementary Table S4: Primers used for Sanger sequencing of *TERT* promoter hotspot loci, *TP53* and selected exons of *PIK3CA*, *HRAS* and *BRAF*.

Gene	Forward	Reverse
TERT promoter	CCAGGGCTTCCCACGTGC	ACTGGGGACCCGGGCACC
TP53 exon 2	ATCCCCACTTTTCCTCTTGC	TCCCACAGGTCTCTGCTAGG
TP53 exon 3	CCATGGGACTGACTTTCTGC	GGGACTGTAGATGGGTGAA
TP53 exon 4	CCTGGTCCTCTGACTGCTCT	GCCAGGCATTGAAGTCTCAT
TP53 exon 5	GACTTTCAACTCTGTCTCCT	CTGGGGACCCTGGGCAACCA
TP53 exon 6	GTCCCCAGGCCTCTGATTC	CTTAACCCCTCCTCCCAGAG
TP53 exon 7	TTATCTCCTAGGTTGGCTCT	CAAGTGGCTCCTGACCTGGA
TP53 exon 8	CCTTACTGCCTCTTGCTTC	TGAATCTGAGGCATAACTGC
TP53 exon 9	TTGCCTCTTTCCTAGCACTG	CCCAAGACTTAGTACCTGAA
TP53 exon 10	CTCTGTTGCTGCAGATC	GCTGAGGTCACTCACCT
TP53 exon 11	TGTCATCTCTCCTCCCTGCT	CAAGGGTTCAAAGACCCAAA
PIK3CA exon 9	CTGTGAATCCAGAGGGGAAA	GCACTTACCTGTGACTCCATAGAA
PIK3CA exon 20	TGGAATCCAGAGTGAGCTTTC	CTCAATGATGCTTGGCTCTG
HRAS exon 3	TGTCCTCCTGCAGGATTC	GTACTGGTGGATGTCCTC
BRAF exon 11	TCCCTCTCAGGCATAAGGTAA	CGAACAGTGAATATTTCCTTTGAT
BRAF exon 15	CATAATGCTTGCTCTGATAGGAA	AGTAACTCAGCAGCATCTCAG
